# Pancancer modelling predicts the context-specific impact of somatic mutations on transcriptional programs

**DOI:** 10.1038/ncomms14249

**Published:** 2017-01-31

**Authors:** Hatice U. Osmanbeyoglu, Eneda Toska, Carmen Chan, José Baselga, Christina S. Leslie

**Affiliations:** 1 Computational and Systems Biology Program, Memorial Sloan Kettering Cancer Center, 1275 York Avenue, Box No. 460, New York, New York 10065, USA; 2 Human Oncogenesis and Pathogenesis Program, Memorial Sloan Kettering Cancer Center, New York, New York 10065, USA

## Abstract

Pancancer studies have identified many genes that are frequently somatically altered across multiple tumour types, suggesting that pathway-targeted therapies can be deployed across diverse cancers. However, the same ‘actionable mutation' impacts distinct context-specific gene regulatory programs and signalling networks—and interacts with different genetic backgrounds of co-occurring alterations—in different cancers. Here we apply a computational strategy for integrating parallel (phospho)proteomic and mRNA sequencing data across 12 TCGA tumour data sets to interpret the context-specific impact of somatic alterations in terms of functional signatures such as (phospho)protein and transcription factor (TF) activities. Our analysis predicts distinct dysregulated transcriptional regulators downstream of somatic alterations in different cancers, and we validate the context-specific differential activity of TFs associated to mutant *PIK3CA* in isogenic cancer cell line models. These results have implications for the pancancer use of targeted drugs and potentially for the design of combination therapies.

## Abstract (web summary)

Cancer genomic data sets contain a wealth of data that can be used to predict prognosis and further understand disease. Here, the authors integrate multiple genomics data types to identify transcriptional dysregulation in response to somatic mutations.

Cancer cells evolve to acquire hallmark capabilities to sustain chronic proliferation, evade growth suppressors and avoid cell death^1^ largely through the accumulation of somatic alterations that disrupt key signalling pathways. Large-scale cancer genomics projects such as The Cancer Genome Atlas (TCGA) have generated a comprehensive catalogue of somatic mutations and copy number aberrations across many tumour types. These alterations have been mapped to known pathways with the hope of deploying pathway-targeted therapeutics—drugs targeting mutant oncoproteins or highly overexpressed wild-type (WT) receptors or signal-transduction proteins—for personalized medicine^2^,^3^,^4^,^5^,^6^,^7^,^8^,^9^,^10^,^11^. However, while the same ‘actionable mutation' may occur in multiple cancers, it interacts with context-specific regulatory and signalling networks as well as the genetic background of other somatic alterations, suggesting that its impact—and the effectiveness of the targeted therapy—may strongly depend both on the cancer type and additional molecular features of the individual tumour. Moreover, the role of many frequent somatic alterations remains obscure, and it is unclear whether and how they interact with targetable pathways. In fact, computational studies of drug sensitivity across cancer cell lines have found that gene expression features are more informative than mutations for predicting response to targeted therapies^12^. Meanwhile, early ‘basket' clinical trials that enroll patients for targeted therapies based on mutation status alone—regardless of cancer type—have demonstrated efficacy only in a subset of cancers^13^,^14^. These findings point to the need for better integrative computational methods that leverage additional molecular readouts to model the context-specific impact of somatic alterations on gene expression programs.

To this end, we applied a computational strategy for exploiting parallel (phospho)proteomic and mRNA sequencing data for large tumour sets by linking the dysregulation of upstream signalling pathways with altered transcriptional response through the transcriptional circuitry^15^,^16^. We then developed a statistical framework to interpret the impact of mutations and copy number events in terms of altered (phospho)protein and transcription factor (TF) activity. We used this strategy to train (phospho)protein–TF interaction models across 12 human cancers in TCGA. First, we identified shared and cancer-specific roles of TF/signalling regulators across cancer types. In bladder urothelial carcinoma, renal cell clear carcinoma and uterus endometrial carcinoma, many of the identified TF regulators were significantly associated with survival outcome. By stratifying tumours by inferred TF activities rather than gene expression patterns, we identified known and previously unlinked TFs that are differentially active in HPV(+) versus HPV(−) head and neck squamous cancer, and we uncovered a subtype of endometrioid uterine cancer harbouring mutant β-catenin with altered TF activities.

We next performed a systematic regularized regression analysis to associate frequent somatic aberrations with changes in inferred TF and (phospho)protein activities in each cancer type. This analysis identified key regulators associated with the major mutations in renal clear-cell carcinoma. More generally, we observed that specific molecular aberrations have cancer-specific functional consequences. In particular, we associated *PIK3CA* activating mutations with altered activities of distinct sets of TFs in different cancers. Notably, in isogenic cell line models of breast cancer and head and neck cancer, we validated the altered activity of several TFs in the presence of mutant *PIK3CA* by measuring promoter occupancy and expression of target genes, confirming the context-specific predictions of our model. These proof-of-principle results suggest a computational strategy for personalized deployment of targeted therapeutics—and potentially for the development of context-specific combination therapies—in a pancancer setting.

## Results

### Pancancer analysis models dysregulated TFs and signalling

**Figure 1:**
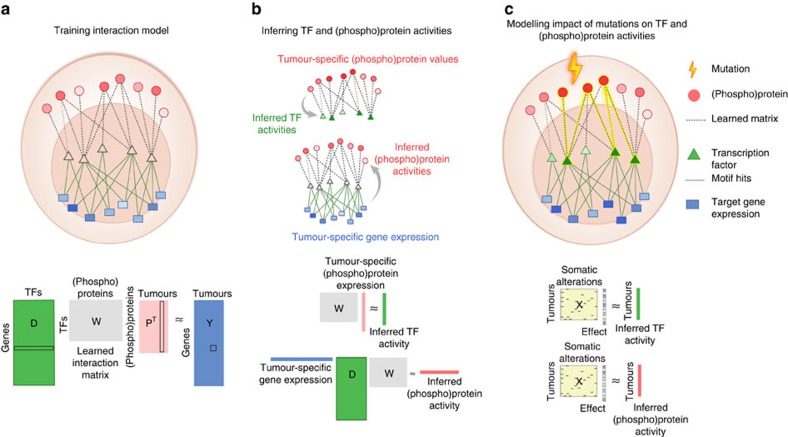
Integrative computational model links signalling to downstream transcriptional programs. (**a**) Formally, an interaction matrix **W** between TFs and upstream signalling proteins is trained using a bilinear regression algorithm, called affinity regression, on RPPA and mRNA expression (RNA-seq) data from a set of tumours, together with TF binding motif information from gene promoters. The model learns to predict target gene expression from tumour-specific (phospho)protein expression levels and gene-specific TF binding sites. The model can be viewed as learning weighted edges (shown as dashed lines) between upstream signalling proteins (shown as red circles) and transcription factors (TFs, shown as triangles) to capture the flow of information from signalling pathways to TFs to target genes and to predict target gene expression changes (TF to target genes shown in green). Formally, the learned weighted edges between (phospho)proteins and TFs are represented by an interaction matrix. (**b**) (Phospho)protein–TF interaction models for each cancer are trained independently. The model can be used to infer sample-specific TF activities from measured RPPA profiles or to infer sample-specific (phospho)protein activities from measured mRNA expression values by use of matrix mappings. (**c**) To model the impact of somatic aberrations on transcriptional response and signalling events, we use regularized regression to predict inferred TF activities (resp. inferred (phospho)protein activities) from somatic alterations. The significance of the effect size (regression coefficient) for each somatic alteration on TF/(phospho)protein activity is estimated by a permutation approach. The eventual goal of the modelling is to understand the cancer-specific downstream effects of targeted therapies and to potentially discover secondary targets for combination drug strategies.

We used a computational strategy for exploiting parallel (phospho)proteomic and transcriptomic data to learn a model that links alterations in signalling (from RPPA data) with downstream changes transcriptional response (measured by mRNA data) via predicted TF binding sites^15^ (Fig. 1a–c). We used a regularized bilinear regression algorithm called affinity regression (AR)^16^ to learn an *interaction matrix* between upstream signal-transduction proteins and downstream TFs that predicts target gene expression (Fig. 1a, bottom). More intuitively, the model learns weighted edges between signalling proteins and TFs to describe the flow of information from change in (phospho)protein level to altered activity of TF to transcriptional changes in target genes (Fig. 1a, top). In a pancancer context, an AR model is trained independently for each cancer type and explains the variation in gene expression across tumours in terms of (phospho)protein variation and presence of TF binding sites (see Methods section).

We can further use the trained AR interaction matrix for each cancer type to obtain different views of each tumour data set via *mappings* (Fig. 1b): given a tumour sample's (phospho)protein expression levels, we can multiply through the model to infer sample-specific TF activities; conversely, given the gene expression profile, we can multiply through the motif hit matrix and the model to infer ‘(phospho)protein activities' that are more informative than the original noisy RPPA data (Fig. 1b, bottom). Intuitively, information flows down from observed RPPA levels through the learned interaction matrix to infer TF activities, and observed mRNA expression levels propagate up through the TF-target edges and interaction network to infer (phospho)protein activities (Fig. 1b, top).

Importantly, by associating the presence of somatic alterations with altered regulator activities, we can gain mechanistic insight into the role of specific mutations or copy number events (Fig. 1c). We perform the association analysis by using regularized regression to predict each inferred TF activity (resp. (phospho)protein activity) individually from the full set of frequent mutation and copy number features (Fig. 1c, bottom; see Methods section). We then evaluate the significance of the effect size (coefficient) for each alteration in the regression model by a permutation approach (see Methods section). After false discovery rate (FDR) correction across TFs/(phospho)proteins, we can identify a significant set of regulators whose altered activities are associated with each mutation/copy number event while controlling for the genetic background of other alterations.

We trained AR models on tumours from 12 different TCGA cancer studies using samples for which mRNA, RPPA, somatic mutation and copy number variation data were available: bladder urothelial carcinoma (BLCA, *n*=115), breast cancer (BRCA, *n*=368), colorectal adenocarcinoma (COADREAD, *n*=150), glioblastoma multiforme (GBM, *n*=58), head and neck squamous carcinoma (HNSC, *n*=194), kidney renal cell-clear carcinoma (KIRC, *n*=376), lung adenocarcinoma (LUAD, *n*=216), lung squamous cell carcinoma (LUSC, *n*=106), ovarian carcinoma (OV, *n*=164), prostate cancer (PRAD, *n*=159), uterine corpus endometrial carcinoma (UCEC, *n*=183), and uterine carcinosarcoma (UCS, *n*=47).

For statistical evaluation, we computed the mean Spearman correlation between predicted and measured gene expression profiles on held-out samples using 10-fold cross-validation for each cancer model. We obtained significantly better performance than a nearest-neighbour approach based on Euclidean distance in the RPPA space (*P*<0.00025, one-sided Wilcoxon's signed-rank test; Supplementary Table 1). Similarly, AR models with true motif and RPPA data outperformed models where motif hits for each gene and RPPA profiles for each tumour were randomized (*P*<0.00025, one-sided Wilcoxon's signed-rank test). When only the motif hits were randomized, the performance improvement of the true model was modest but still significant (*P*<0.00074, one-sided Wilcoxon's signed-rank test), suggesting that the motif data, while noisy, contributes to predictive performance. AR obtained similar performance advantages when assessed using a single held-out test set or when evaluating Pearson correlation or root mean-squared error (Supplementary Figs 1–5).

### Pancancer AR identifies signatures of survival

**Figure 2:**
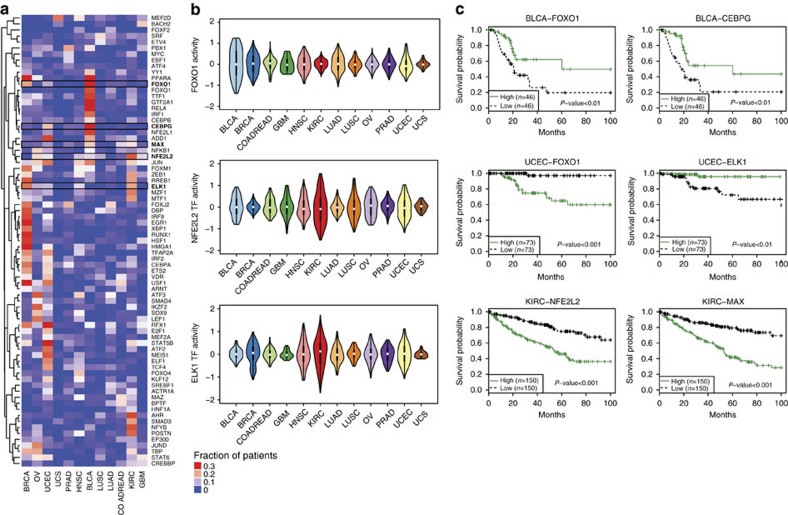
Activities of significant regulatory TFs correlate with patient survival. (**a**) Significant TF regulators for each cancer type were identified using an empirical null model based on training affinity regression models randomized gene expression data (see Methods section). A FDR of 10% was used for studies with >300 samples (BRCA, KIRC), 15% FDR for mid-size studies (BLCA, COADREAD, HNSC, LUAD, LUSC, UCEC, OV, PRAD), 25% FDR for studies with <50 samples (GBM, UCS). The heat map shows the fraction of samples where each TF is identified as a significant regulator within each cancer. For clarity, the union of top 10 most prevalent significant TFs in each cancer-specific model is shown. (**b**) Violin plots indicate the distribution of inferred FOXO1, NFE2L2 and ELK1 TF activities across cancer types. For example, FOXO1 TF activity is highly variable across tumours in BLCA, BRCA and UCEC. (**c**) Inferred TF activity predicts survival in patients with BLCA, UCEC and KIRC cancers. Kaplan–Meier survival curves for TCGA BLCA samples, stratified by TF activity of FOXO1 (top left), CEBPG (top right); TCGA UCEC samples stratified by TF activity of FOXO1 (middle left), ELK1 (middle right); TCGA KIRC samples stratified by TF activity of NFE2L2 (bottom left), MAX (bottom right).

To assess the statistical significance of AR-inferred regulator activities, we developed an empirical null model based on training AR models on randomly permuted gene expression profiles for each tumour type (see Methods section). Then, we asked whether the value of individual TF/(phospho)protein activities for each sample were significantly low or high relative to the corresponding distribution over permuted data. We corrected for FDR across TFs/(phospho)proteins (see Methods section) and identified significant shared and cancer-specific TF/(phospho)protein regulators (Fig. 2a and Supplementary Data 1).

Figure 2a shows the fraction of samples per cancer type where each TF was identified as a significant regulator; for clarity, only the union of top 10 most prevalent significant TFs per cancer are shown. Certain TFs display a large variation in inferred activity in specific cancer types, suggesting a key role in regulating target gene expression in these cases, while having more modest variation in other cancers. Figure 2b shows the inferred activity distribution of three TFs identified from our analysis: FOXO1 (Forkhead box O), NFE2L2 and ELK1. FOXO1, a key regulator of cell-cycle progression and apoptosis, was identified as a significant regulator for more than 10% of tumours in BLCA, BRCA and UCEC; its activity showed high variation among tumours for these particular cancers (Fig. 2b, top panel).

A number of TFs were significantly altered in two or more tumour types, including ZEB1, JUN, ELK1, FOXM1, while others were limited to a single type, such as FOXD1 in HNSC and FOXL1 in KIRC. We identified TFs that are known cancer drivers such as STAT5 (endometrioid carcinoma^17^), AHR^18^, HMGA^19^ (KIRC), PBX1 (OV^20^, prostate cancer^21^, BRCA^22^,^23^) and NFE2L2 (squamous cell lung cancer). Other predicted TF–cancer relationships are unknown and may provide new mechanistic insights.

To investigate the clinical relevance of these findings, we examined whether the inferred activity of significant TFs was linked to patient survival. We fit Cox proportional hazards regression models for each TF activity using clinical stage (or histological subtype for UCEC) as an additional covariate. Indeed, many identified TF regulators had highly significant associations with survival outcome in BLCA, KIRC and UCEC (Fig. 2c and Supplementary Tables 2–4). For instance, FOXO1 was associated with survival in BLCA and UCEC, and its inferred activity separated patients into high- and low-risk groups. Previous immunohistochemical analyses of FOXO1 in bladder cancer showed that increased mRNA expression is associated with reduced disease progression^24^, consistent with our result. Inferred NFE2L2 and MAX activity were associated with patient survival in KIRC, as was ELK1 activity in the UCEC study. Importantly, Cox models built from inferred TF activities achieved more significant patient stratification than models built from the gene expression of significant TFs (BLCA: *P*<10^−4^; UCEC: *P*<10^−10^, one-sided paired Wilcoxon's signed-rank test) (see Methods section and Supplementary Tables 2–4). We further confirmed that most of our UCEC survival results generalized to two other independent cohorts, MDACC (MD Anderson Cancer Center) and Bergen^25^ (see Methods section and Supplementary Table 5), supporting the use of inferred TF activity for patient stratification.

### TF activities distinguish HPV(+) and HPV(−) HNSC patients

**Figure 3:**
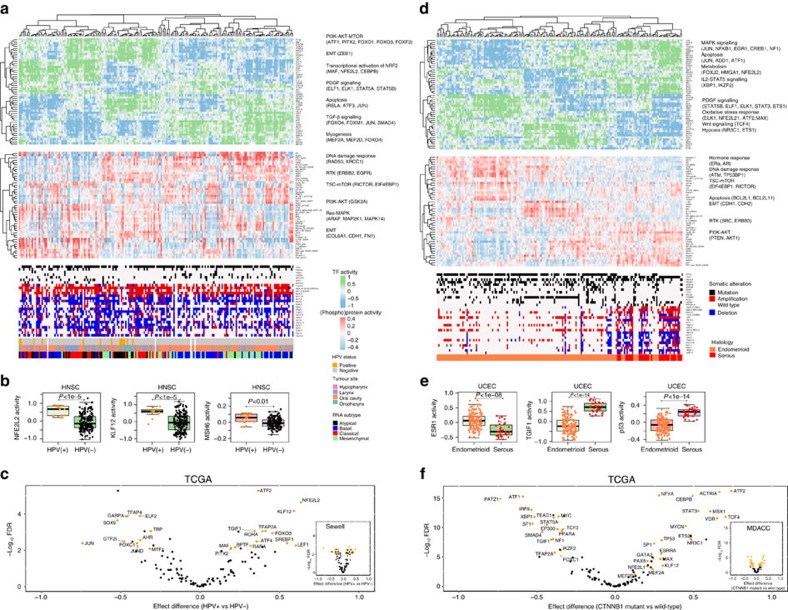
Pancancer affinity regression modelling identifies regulatory features of tumour subtypes. (**a**) We trained an affinity regression model on 194 tumours from the TCGA HNSC study. The top heat map shows tumours clustered by the inferred TF activities with highest variance. The middle panel shows top most variable (∼50) inferred (phospho)protein activities for each tumour based on clustering by sample-specific TF activities. The bottom panel shows genomic aberration profiles of each tumour as well as HPV status, tumour site and mRNA expression subtypes derived from the corresponding TCGA HNSC study. (**b**) Examples of three of the significantly differential inferred TF/protein activities in HPV(+) versus HPV(−) tumours were NFE2L2, KLF12 and MSH6. HPV(+) tumours have significantly higher NFE2L2 TF activity (*P*<10^−5^, Wilcoxon's rank-sum test), higher KLF12 TF activity (*P*<10^−5^, Wilcoxon's-rank sum test) and higher MSH6 protein activity (*P*<10^−2^, Wilcoxon's rank-sum test) than HPV(−) tumours. (**c**) The mean inferred TF activity difference in HPV(+) and HPV(−) patients is plotted on the *x* axis, and FDR-adjusted significance from *t*-test is plotted on the *y* axis (–log10 scale) for TCGA and Sewell *et al*.^28^ head and neck cancer cohorts. TFs significantly associated with HPV status (FDR<0.01) in both cohorts are coloured in orange. (**d**) We trained an affinity regression model on 183 tumours from the TCGA endometrial carcinoma (UCEC) study. The top heat map shows a clustering of tumours by inferred TF activities. The middle panel shows inferred (phospho)protein activities of each tumour based on clustering of tumour TF activities. The bottom panel shows genomic aberration profiles of each tumour as well as histological subtypes derived from the corresponding TCGA UCEC study. Patterns of TF activities across tumours often correlated with patterns of (phospho)protein activities. (**e**) Examples of three of the significantly differential inferred TF/(phospho)protein activities in *serous* versus *endometrioid* tumours were ESR1, TGIF1 and p53. Tumours with endometrioid histology have significantly higher ESR1 TF activity (*P*<10^−8^, Wilcoxon rank sum test), lower TGIF1 TF activity (*P*<10^−14^, Wilcoxon rank-sum test), and lower p53 protein activity (*P*<10^−14^, Wilcoxon rank sum test) than serous tumours. (**f**) The mean inferred TF activity difference in *CTNNB1* mutant and *CTNNB1* wild-type patients is plotted on the *x* axis, and false discovery rate (FDR)-adjusted significance from *t*-test is plotted on the *y* axis (−log 10 scale) for TCGA and MDACC endometrial cancer cohorts. TFs significantly associated with *CTNNB1* status (FDR<0.01) in both cohorts are coloured in orange. Box edges represent the upper and lower quantile with median value shown as bold line in the middle of the box. Whiskers represent 1.5 times the quantile of the data.

Next, we asked whether our method could identify known and novel TFs that are differentially active in cancer subtypes. Figure 3a shows the clustering of tumours by inferred TF activities, together with inferred (phospho)protein activities for the same tumour ordering, as derived from the HNSC model (showing TF/(phospho)protein activities with largest standard deviation across samples). Notably, patterns of TF activities across tumours generally correlated with (phospho)protein activities.

Head and neck squamous cancer is frequently associated with human papillomavirus (HPV) infection and mutations in *TP53*. AR analysis suggests that the molecular pathogenesis of HPV(+) head and neck cancer is distinct from HPV(−) tumours. Inferred TF activities of 33 TFs were significantly associated with HPV status (*t*-test, FDR-corrected *P*<0.01, Fig. 3b,c); by contrast, the gene expression values of only two TFs were associated with HPV status (Supplementary Table 6). Altered TF activities were involved in cell-cycle, apoptosis, oxidative stress, WNT signalling and transforming growth factor-β) signalling and may have roles in the initiation and maintenance of HPV(+) head and neck cancer^26^. For example, KLF12 and NFE2L2 were significantly associated with HPV(+) tumours (Fig. 3b). Interestingly, the *KLF12* locus is a frequent integration site for the HPV virus^27^ in cervical cancer, and the TCGA HNSC study also identified *KLF5*, the locus of a related KLF factor, as an HPV integration site^5^. To confirm our results, we used the HNSC TCGA-trained AR model to infer TF activities in an independent set of 42 head and neck cancer RPPA profiles with HPV status^28^. We again identified TF–HPV status associations by *t*-test and found a similar set of TFs (23 out of 33) whose activities significantly differed between HPV(+) and HPV(−) tumours; the 33 identified TFs were also enriched among the top-ranked TFs in the new cohort (*P*<10^−5^, Mann–Whitney test) (Fig. 3c and Supplementary Table 6).

As described previously^29^, mutant *TP53* tends to be mutually exclusive with HPV(+) status, but inferred TP53 TF activity and inferred p53 protein activity were not significantly different between HPV(+) and HPV(−) patients (*t*-test *P*=0.477 and *P*=0.741, respectively). However, it is known that the viral E6 oncoproteins in HPV(+) head and neck cancer form a complex with WT p53 and lead to its degradation^30^, pointing to an alternative mechanism for p53 inactivation in HPV(+) patients.

We performed similar analyses for other TCGA cancer studies and in each cancer type could stratify patients by regulator activity profiles (Supplementary Figs 6–15). For example, the inferred TF activity of CEBPA^31^ was significantly higher in the mesenchymal subtype compared to other subtypes of GBM (*P*<10^−5^, Wilcoxon's rank-sum test used for all tests); ESR1 (estrogen receptor 1) activity was higher in luminal BRCA compared to other BRCA subtypes (*P*<10^−42^), consistent with oestrogen receptor serving as a luminal marker^32^; and activity of TTF-1 (thyroid transcription factor-1) thyroid transcription factor-1, a known biomarker of LUAD^33^, was higher in the squamoid (*P*<10^−10^) and bronchioid subtypes (*P*<10^−5^) compared to the magnoid subtype in LUAD.

### TF signature defines mutant *CTNNB1* endometrioid subtype

We then asked if we could associate inferred TF activities with mutational signatures as a first step towards developing a more general statistical strategy. Figure 3d shows a clustering of tumours by inferred TF activities from the UCEC model, together with inferred (phospho)protein activities and recurrent somatic mutations and copy number events. Serous-like endometrial tumours are hormone receptor negative, mostly copy number high, and harbour mutations in *TP53*, whereas endometrioid tumours are hormone receptor positive, copy number low, and have a high frequency of PI3K-AKT (phosphatidylinositol 3-kinase-AKT) pathway alterations^5^,^6^. Consistent with their distinct molecular and genomic features, we found significant differences in inferred regulator activities in serous-like and endometrioid tumours (Supplementary Tables 7 and 8), including increased ESR1 activity in endometrioid tumours (*P*<10^−8^, Wilcoxon's rank-sum test used for all tests) and increased TGIF1 TF activity and inferred p53 protein activity serous-like tumours (*P*<10^−14^ for both tests; Fig. 3e).

Importantly, clustering by TF activities revealed subclasses of tumours within each histological subtype that sometimes correlated with mutation status. In particular, endometrioid tumours with a *CTNNB1* mutation form a distinct cluster based on inferred TF activity profiles that was not observed by clustering TF mRNA expression levels directly (Supplementary Fig. 16). Moreover, clustering based on inferred TF activity was better able to stratify patients by *CTNNB1* mutation status (*P*<10^−17^, two-sided *χ*^2^ test for all tests) compared to reported TCGA mRNA clusters (*P*<0.01) and TCGA integrated clusters (*P*<10^−6^) (Supplementary Tables 9 and 10). Significant inferred TF activity differences between *CTNNB1* mutant and WT patients (satisfying FDR-corrected *P*<0.01, *t*-test) associated *CTNNB1* mutant status with altered activity of TFs involved in WNT signalling, epithelial–mesenchymal transition and cancer stem cell transition including TCF4 (transcriptional factor 4), NFATC4, JUN, TP53, MAX, MYC, STAT3 and KLF12 (Fig. 3f). We confirmed these results in an independent data set of 203 endometrial RPPA profiles along with mutation and clinical data compiled by MDACC^25^, using the UCEC TCGA-trained AR model to infer TF activities, and replicated many of the TFs associated with mutant *CTNBB1* (*P*<10^−5^, Mann–Whitney test; Fig. 3f and Supplementary Table 11). Interestingly, another study performed customized consensus clustering on TCGA UCEC expression data and did identify a cluster enriched with β-catenin mutations, and GSEA (gene set enrichment analysis) suggested an association with WNT signalling, consistent with our analysis^34^.

### Modelling reveals impact of mutations in kidney cancer

Encouraged by our findings for mutant *CTNBB1* endometrioid tumours, we developed a systematic statistical approach for modelling the impact of somatic alterations on regulator activity in each tumour type, with the eventual goal of deciphering cancer-specific downstream effects of targeted therapies and potentially discovering secondary targets for combination drug strategies. First, we implemented a regularized regression approach that uses somatic alterations to explain inferred TF/(phospho)protein activity across tumour samples on a regulator-by-regulator basis. For a complex genotype, the model explains TF/(phospho)protein regulator activity across tumours as the sum of effects of individual somatic alterations (that is, coefficients in the regression model), and the effect size of each alteration is assigned a nominal *P* value by a permutation approach (see Methods section). We then corrected for multiple hypotheses across regulator models, treating inferred TF activities and inferred (phospho)protein activities as separate groups of tests (see Methods section). Combining these results identified a set of regulators predicted to be significantly dysregulated by each somatic alteration in each TCGA cancer study.

**Figure 4:**
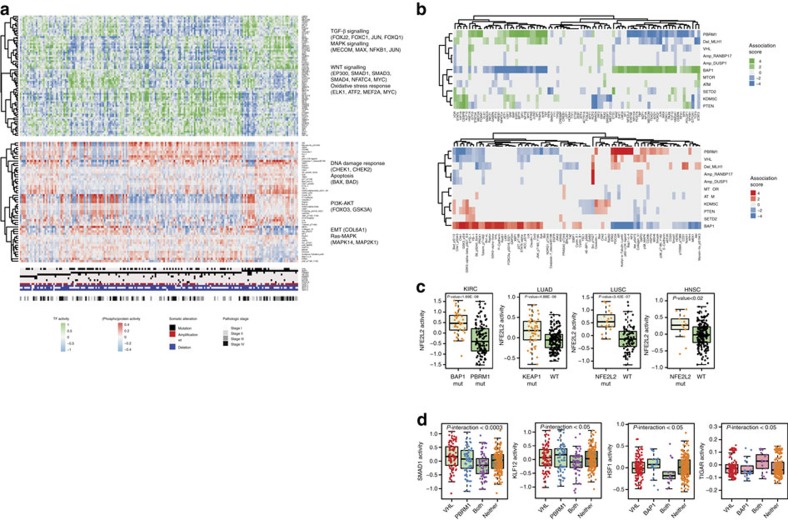
Genomic aberrations are associated with dysregulated TF and (phospho)protein activity in the TCGA KIRC study. (**a**) We trained an affinity regression model on 376 tumours from the TCGA KIRC study. The top panel shows inferred TF activity for each tumour associated with *BAP1*, *PBMR1*, *SETD2* and *KDM5C* mutations, ordered according to the mutation profile. The middle panel shows the corresponding (phospho)protein activity for each tumour associated with *BAP1*, *PBMR1*, *SETD2* and *KDM5C* mutations. The bottom panel shows genomic aberration profiles of each tumour as well as the pathological stage as derived from the corresponding TCGA KIRC study. (**b**) Impact of genomic aberrations on individual TF/(phospho)protein activities in TCGA KIRC, based on a regularized regression analysis. A permutation test approach was used to assign significance to ridge regression coefficients (see Methods section). The heat map shows –log 10 FDR-adjusted *P* values derived by permutation test, multiplied by the sign of the coefficient. The top panel shows inferred TF activity associations. The bottom panel shows inferred (phospho)protein activity coefficients. (**c**) Inferred NFE2L2 activity in TCGA KIRC, LUAD, LUSC and HNSC studies and impact of mutations using integrative modelling. In the KIRC study, tumours with mutant *BAP1* have significantly higher NFE2L2 activity than mutant *PBRM1* tumours (*P*<1.68 × 10^−8^, Wilcoxon's rank-sum test). Tumours with mutant *KEAP1*/*NFE2L2* have also significantly higher inferred TF activity of NFE2L2 (a substrate targeted by KEAP1) than WT tumours in the LUAD study (*P*<4.88 × 10^−6^, Wilcoxon's rank-sum test), LUSC study (*P*<3.42 × 10^−6^, Wilcoxon's rank-sum test) and HNSC study (*P*<0.02, Wilcoxon's rank-sum test). This association is not significant using the original measured gene expression values of NFE2L2 (Supplementary Fig. 17). (**d**) Inferred SMAD1 and KLF12 activity is significantly and synergistically decreased in tumours with both *VHL* and *PBMR1* mutations (SMAD1: interaction, *P*<0.0003; KLF12: interaction, *P*<0.05). In tumours with both *VHL* and *BAP1* mutations, HSF1 activity is significantly decreased (interaction *P*<0.05) and TIGAR protein activity is significantly increased (interaction *P*<0.05). Box edges represent the upper and lower quantile with median value shown as bold line in the middle of the box. Whiskers represent 1.5 times the quantile of the data.

Figure 4a,b shows the regulator activities associated with somatic aberrations in KIRC. Our model identified mutations in *VHL* (von Hippel-Lindau), *PBMR1*, *BAP1*, *MTOR*, *ATM*, *SETD2*, *KDM5C* and *PTEN* (phosphatase and tensin homolog), as well as copy number changes in *MLH1*, *DUSP1* and *RANDBP17* as significantly associated with various TF activity changes across tumours. KIRC is characterized by a high-frequency inactivating mutation in the *VHL* gene found in ∼54% of tumours in TCGA and likely more prevalent^35^. Mutually exclusive mutations in *PBRM1*, a subunit of the PBAF SWI/SNF chromatin remodelling complex, and in histone deubiquitinase *BAP1* define two genetic subtypes of KIRC, while recurrent mutations in the histone methyltransferase *SETD2* also occur.

KIRC samples with *PBMR1* and *BAP1* mutations showed distinct patterns of TF and protein activities (Fig. 4a), and regression analysis associated different regulators with these mutations (Fig. 4b). *PBMR1* mutant tumours are associated with increased activity of TFs/(phospho)proteins that have roles in interleukin signalling and MYC, while regulators with increased activity in *BAP1* mutant tumours are involved in DNA damage response, apoptosis, insulin signalling and mTOR signalling.

Notably, NFE2L2 TF activity was significantly higher in *BAP1* mutant tumours than *PBMR1* mutant tumours. Dysregulation of the KEAP1-NFE2L2 pathway occurs through both genetic and epigenetic mechanisms and induces prosurvival genes promoting proliferation and chemoresistance^36^. Mutations in *KEAP1*, *NFE2L2 (Nrf2)*, *CUL3* or *RBX1* are the most common mechanisms that impair KEAP1-mediated degradation of NFE2L2 and thereby activate the transcriptional effects of NFE2L2. Somatic aberrations in these genes have been described in LUSC, LUAD and HNSC, and indeed we confirmed this activating effect (Fig. 4c). Inferred TF activity of NFE2L2 was increased in mutant versus WT KEAP1 or NFE2L2 lung cancers; these differences are not observed at the gene expression level (Supplementary Fig. 17).

We also assessed synergistic effects by building linear models with interaction terms for each pair of somatic alterations (see Methods section). In samples where *VHL* was comutated with *PBMR1*, SMAD1 (interaction *P*<0.0003) and KLF12 (interaction *P*<0.05) activity were significantly decreased. Meanwhile, when *VHL* was comutated with *BAP1*, HSF1 activity was significantly decreased (interaction *P*<0.05), while TIGAR protein activity was significantly increased (interaction *P*<0.05) (Fig. 4d).

### PI3K pathway mutations dysregulate cancer-specific TFs

**Figure 5:**
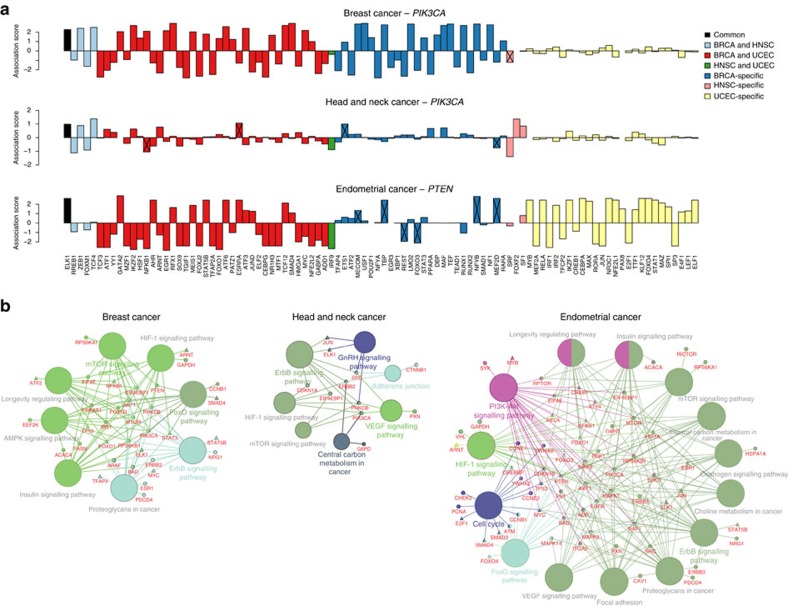
Somatic aberrations in the PI3K pathway dysregulate cancer-specific TFs. (**a**) Bar plots show 134 TFs associated with somatic aberrations in *PTEN* or *PIK3CA* discovered from regularized regression analysis in breast cancer (BRCA), head and neck squamous carcinoma (HNSC) or UCEC and their association scores. TFs that are significantly associated with the somatic aberration but not identified as significant regulators for explaining gene expression in at least 1% of samples are indicated with crossed lines. (**b**) A functionally grouped network of enriched categories was generated for TFs associated with *PIK3CA*/*PTEN* mutations using KEGG pathway terms related to cancer as nodes and linked using ClueGO^79^ analysis. Only the most significant terms in the group are labelled. Functionally related groups partially overlap.

The PI3K pathway controls proliferation, metabolism, survival and motility and is frequently activated in many cancers, often via mutations in *PIK3CA*, which encodes the α-isoform of the p110 catalytic subunit of PI3K (PI3Kα); loss of PTEN, which antagonizes PI3K function; and overexpression of membrane-bound receptor tyrosine kinase^37^,^38^. As PI3K inhibitors are currently in early-phase or phase III trials for use across multiple cancers^14^,^39^, we asked whether PI3K pathway alterations dysregulate the same or differing TFs and (phospho)proteins across tumour types. Figure 5a and Supplementary Fig. 18 show the regulators associated with somatic aberrations in *PTEN* and *PIK3CA* by our analysis in BRCA, HNSC, UCEC, KIRC, LUAD and PRAD tumours (see Methods section).

Activating mutations in *PIK3CA* were present in ∼31% of BRCA tumours and 20% of HNSC tumours^5^. Mutations often occur in one of three hotspot locations (E545K, E542K and H1047) and promote constitutive signalling though the pathway. In UCEC, ∼66% of tumours have *PTEN* inactivating mutations, ∼50% have *PIK3CA* activating mutations and ∼35% have a comutation of *PTEN* and *PIK3CA*. Figure 5a shows 134 TFs associated with somatic aberrations in *PTEN* or *PIK3CA* in BRCA, HNSC or UCEC. Notably, the number of TFs dysregulated by PI3K pathway alterations varied widely across different cancers (9 in HNSC, 65 in BRCA and 63 in UCEC), with striking changes in ErbB/MAPK, mTOR, HIF-1, VEGF and PI3K-Akt pathways. Only ELK1, a TF downstream of the MAPK/ERK pathway, is dysregulated by *PIK3CA* or *PTEN* mutations in all three cancers. Four TF associations were shared in BRCA and HNSC: ZEB1 (EMT activator), TCF4 (Wnt signalling), RREB1 (Ras signalling) and FOXM1 (cell proliferation, cell cycle progression, cell differentiation, DNA damage repair, tissue homeostasis, angiogenesis and apoptosis); 35 TFs were common to BRCA and UCEC; and just IRF9 was shared between UCEC and HNSC. There were also associations unique to each cancer type, including USF1 and SMAD1 for BRCA, FOXF2 for HNSC, and NFE2L2 and NR3C1 for UCEC.

Interestingly, many TF activities were associated with mutant *PTEN* irrespective of *PIK3CA* status in endometrial cancer (Supplementary Fig. 19), consistent with a recent preclinical study^40^, while *PIK3CA* mutations were only significantly associated with a single TF, CREB1. Therefore, *PTEN* and *PIK3CA* appear to have distinct consequences for PI3K activation in UCEC.

PI3K pathway inhibition is known to alter STAT5 (ref. 41), FOXO, RUNX2 (ref. 42), ERG1 (ref. 43) and ETS1 (ref. 44) activities, consistent with our results. We also examined protein microarray-based AKT1 kinase assay and SILAC-based phosphoproteomic data from isogenic knock-in breast cell lines harbouring mutations of PIK3CA^45^ (see Methods section). Of 11 TFs represented in the phosphoproteomic data and associated with mutant *PIK3CA* in BRCA, eight of them—ADD1, FOXO3, HMGA1, HSF1, JUND, NF1, POU2F1, STAT3—showed protein abundance change in isogenic cell line systems (see Methods section and Supplementary Table 12). Moreover, of the six TFs represented in the AKT1 kinase assay^45^ and associated with mutant *PIK3CA* in BRCA, four of them—ETS1, ATF6, SOX9 and TEAD1—were identified as AKT substrates (Supplementary Table 13).

### Predicted TFs for mutant *PIK3CA* validate experimentally

**Figure 6:**
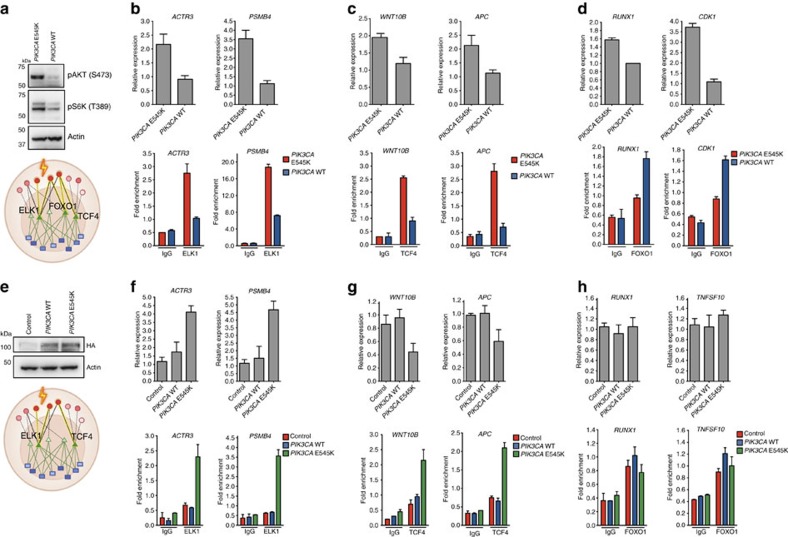
Predicted transcriptional regulatory impacts of activating *PIK3CA* **mutations validated experimentally**. (**a**) Western blot analysis of pAKT (S473), pS6K (T389) and actin in parental MCF7 cells that carry the *PIK3CA* E545K mutation and in ‘corrected' WT PIK3CA cells. ELK1 activity in PI3Kα mutant cells. (**b**) *ACTR3* and *PSMB4* mRNA expression in parental *PIK3CA* mutant and *PIK3CA* WT cells. ChIP assays with control IgG or ELK1 antibodies for *ACTR3* and *PSMB4* in parental *PIK3CA* mutant and *PIK3CA* WT MCF7 cells. The data are presented as fold-enrichment relative to the actin control gene region. TCF4 activity in PI3Kα mutant cells (mean±s.d., n=3 independent experiments). (**c**) *WNT10B* and *APC* mRNA expression in parental *PIK3CA* mutant and *PIK3CA* WT cells. ChIP assays with control IgG or TCF4 antibodies for *WNT10B* and *APC* in parental *PIK3CA* mutant or *PIK3CA* WT MCF7 cells. The data are presented as fold-enrichment relative to the actin control gene region (mean±s.d., n=3 independent experiments). FOXO1 activity in PI3Kα mutant cells. (**d**) *RUNX1* and *CDK1* mRNA expression in parental *PIK3CA* mutant and *PIK3CA* WT MCF7 cells. Parental and WT MCF7 cells were subjected to ChIP assays with control IgG or FOXO1 antibodies. The data are presented as fold-enrichment relative to the actin control gene region (mean±s.d., n=3 independent experiments). (**e**) Transfected vector control, WT *PIK3CA* or *PIK3CA* E545K Cal27 cells were subjected to western blots with haemagglutinin (HA) and actin antibodies after 48 h of transfection. (**f**) *ACTR3* and *PSMB4* mRNA expression in control, *PIK3CA* WT and *PIK3CA* E545K cells. ChIP assays with control IgG or ELK1 antibodies in control, WT and *PIK3CA* E545K cells (mean±s.d., n=3 independent experiments). (**g**) *WNT10B* and *APC* mRNA expression in control, *PIK3CA* WT- and *PIK3CA* E545K-transfected Cal27 cells. ChIP assays with control IgG or TCF4 antibodies in control-, *PIK3CA* WT- and *PIK3CA* E545K-transfected Cal27 cells (bottom panel). (**h**) *RUNX1* and *TNSF10A* mRNA expression in control, *PIK3CA* WT- and *PIK3CA* E545K-transfected Cal27 cells. ChIP assays with control IgG or FOXO1 antibodies in control-, WT- and *PIK3CA* E545K-transfected Cal27 cells. For other TF targets see Supplementary Fig. 21 and Supplementary Fig. 23.

Our analysis associated mutant *PIK3CA* with ELK1 and TCF4 activity in both breast and head and neck cancer, and with FOXO1 activity in BRCA but *not* in head and neck cancer. We validated these predictions in isogenic BRCA and head and neck cancer cell lines by measuring promoter occupancy via chromatin immunoprecipitation-quantitative PCR (ChIP-qPCR) and expression change via quantitative reverse transcription-qPCR (RT-qPCR) of canonical target genes of these TFs (Fig. 6 and Methods section).

First, we used the parental MCF7 cell line carrying the *PIK3CA* E545K mutation and an MCF7 *PIK3CA* WT cell line in which the mutation was corrected using gene targeting^46^. Western blotting confirmed that WT *PIK3CA* cells have very low PI3K pathway activation compared to mutant parental cells, with strongly reduced levels of phospho (p)-AKT and p-S6K (Fig. 6a and Supplementary Fig. 20).

Quantitative RT-qPCR analysis of the well-described ELK1 target genes *ACTR3*, *PSMB4 (*ref. 47), *WNK1*, *PAPLN*, *FOXP4* and *DDX27* confirmed significant increases in mRNA levels in the parental *PIK3CA* mutant cells compared to *PIK3CA* WT cells, with the exception of *PAPLN*, where we observed a significant decrease (Fig. 6b, top panel; Supplementary Fig. 21a). Moreover, ChIP-qPCR experiments confirmed that ELK1 binding to all five target gene promoters was significantly increased in the *PIK3CA* mutant MCF7 compared to WT cells, showing that mutant *PIK3CA* enhances ELK1 transcriptional activity in BRCA cells (Fig. 6b, bottom panel).

Well-known TCF4 target genes such as *WNT10B*, *APC*, *FBXW11* and *PPP2R5E* were differentially regulated by the *PIK3CA* E545K mutation in MCF7 cells (Fig. 6c, top panel and Supplementary Fig. 21b), and ChIP-qPCR analysis confirmed enhanced binding of TCF4 to their promoters in mutant *PIK3CA* cells (Fig. 6c, bottom panel and Supplementary Fig. 20b). Similarly, PCR with reverse transcription (RT–qPCR) analysis of FOXO1 target genes *RUNX1*, *CDK1*, *CAMKK1* and *TNFSF10* (ref. 48) mRNA confirmed that their mRNA levels were differentially regulated by the *PIK3CA* E545K mutation (Fig. 6d, top panel and Supplementary Fig. 21c), and ChIP-qPCR analysis confirmed increased binding of FOXO1 to their promoters in the wild-type *PIK3CA* MCF7 cells relative to parental PIK3CA E545K cells (Fig. 6d, bottom panel and Supplementary Fig. 21c).

We also performed validation experiments in the head and neck cancer cell line Cal27, which is WT for the *PIK3CA* gene. Control, *PIK3CA* WT or *PIK3CA* E545K vectors were overexpressed in Cal27, and ChIP-qPCR and RT-qPCR expression experiments were performed to investigate the activity of ELK1, TCF4 and FOXO1. Western blotting confirmed successful expression of these vectors in the Cal27 cell line (Fig. 6e and Supplementary Fig. 22).

Like in the MCF7 BRCA model, RT-qPCR analysis demonstrated an increase in the mRNA levels of four known ELK1 target genes, *ACTR3*, *PSMB4*, *WNK1* and *DDX27*, in Cal27 cells transfected with PIK3CA E545K compared to Cal27 cells transfected with WT *PIK3CA* and control cells (Fig. 6f, top panel and Supplementary Fig. 23a). Increased occupancy of ELK1 at target promoters was confirmed by ChIP-qPCR assays only when cells were transfected with the *PIK3CA* E545K vector (Fig. 6f, bottom panel and Supplementary Fig. 23a). Thus, ELK1 transcriptional activity is enhanced by mutant *PIK3CA* in both head and neck and BRCA models. RT-qPCR analysis demonstrated an increase in the mRNA levels of four TCF4 target genes in Cal27 cells with PIK3CA E545K compared to WT Cal27 and control cells (Fig. 6g, top panel and Supplementary Fig. 23b). Increased occupancy of TCF4 at the promoters of these genes was confirmed by ChIP assays only when cells were transfected with the *PIK3CA* E545K vector (Fig. 6g, bottom panel and Supplementary Fig. 23b). Thus, mutant *PIK3CA* enhances TCF4 transcriptional activity in head and neck as well as BRCA models.

FOXO1 activity was not associated with mutant *PIK3CA* in our HNSC model. Indeed, RT-qPCR analysis demonstrated no significant change in the mRNA levels of the FOXO1 target genes in PIK3CA E545K Cal27 cells compared to WT Cal27 cells and control cells (Fig. 6h, top panel and Supplementary Fig. 23c). Further, no change in occupancy of FOXO1 at the promoters of *TNFSF10* and *RUNX1* was shown by ChIP assays when cells were transfected with the *PIK3CA* E545K vector (Fig. 6h, bottom panel and Supplementary Fig. 23c).

ELK1 is phosphorylated through activation of the MAPK/ERK pathways and translocates to the nucleus, resulting in activation/repression of downstream targets^47^,^49^,^50^,^51^,^52^ that are important in cell proliferation, apoptosis, cell migration and invasion, and inflammatory response^53^,^54^. Immunohistochemistry in breast tumour specimens has shown that the levels of p-ELK1 expression are significantly elevated in luminal and Her-2-negative BRCA subtypes^55^, but how Elk1 is activated in BRCA is not known. Our computational and experimental results suggest that a potential mechanism for ELK1 activation is through an activating *PIK3CA* mutation.

TCF4 interacts with β-catenin to mediate Wnt signalling and has been implicated in colorectal tumorigenesis^56^,^57^. Our analyses confirm that TCF4 activation may result from an activating *PIK3CA* mutation. Recently, a small-molecule inhibitor of the β-catenin/TCF4 interaction called LF3 has been shown to diminish Wnt-dependent biologic characteristics of colon cancer cells, inhibit their self-renewal capacity and induce their differentiation^58^. Since we demonstrated altered transcriptional activity of TCF4 downstream of mutant *PIK3CA* in breast and head and neck cancer cells, targeting TCF4 might be new therapeutic strategy in *PIK3CA* mutant patients.

FOXO TFs, including FOXO1, are implicated in the regulation of stress resistance, metabolism, cell cycle, apoptosis and DNA repair. It is well known that constitutive PI3K-AKT pathway activation causes downregulation of FOXO tumour suppressor functions in BRCA^59^. However, regulation of FOXO target genes is multifactorial, and based on our findings, context-dependent. Specifically, we showed that an activating *PIK3CA* mutation altered FOXO1 activity in the BRCA model but not in the head and neck cancer model, consistent with the context-specific predictions of our algorithm. This shows one example of how a clinically relevant ‘actionable mutation' impacts regulatory programs in a cancer-specific manner, giving clues about druggability across tumour types.

## Discussion

Many targetable alterations are present across multiple tumour types. For example, activating mutations and amplifications of PIK3CA are targetable by PI3K inhibitors, which are in active clinical assessment in combination therapies with RTK inhibitors and antioestrogen therapies in BRCA, antiandrogen therapy in PRAD and MEK inhibitors in many solid tumours^60^,^61^,^62^,^63^,^64^,^65^. However, the cancer-specific context can impact how patients respond to targeted therapies, since the targeted protein resides in a network of interacting proteins and is subject to extensive feedback and crosstalk between signalling pathways. In recent clinical trials of targeted therapies (for example, Gleevec in chronic myelogenous leukaemia, herceptin in BRCA, BRAF inhibitors in melanoma), patients who share the targeted mutation and tumour type displayed highly variable responses to the drugs^66^. Therefore, a systematic stratification of tumours that goes beyond therapeutically actionable alterations and incorporates other functional readouts—for example, dysregulated TF and (phospho)protein signatures derived from our model—may better predict which patients will benefit from targeted and combination therapies.

Using inferred TF/protein activities in tumours may also reveal clinically relevant patient subgroups. Patients with endometrioid carcinomas display heterogeneous clinical courses and response to therapy, despite similar tumour histopathology. Clustering UCEC tumours by TF activities revealed a subclass of endometrioid tumours that correlated with β-catenin mutation status and had poorer survival (Supplementary Fig. 24, *P*<0.001). Linking mutant β-catenin to putative downstream TF effectors could inform future mechanistic studies—for example, short hairpin RNA or CRISPR/Cas screening to identify TFs whose deletion/knockdown leads to changes in proliferation—to develop new therapeutic strategies.

Previous algorithms to interpret the role of somatic alterations have examined enrichment of mutations in known pathways^3^,^4^,^6^ or searched for alterations that represent mutual exclusive patterns^2^, subnetworks^10^,^67^,^68^,^69^ or modules^70^. These approaches examine co-occurrences of somatic alterations in a known protein interaction network without explicitly modelling their impact on transcriptional programs or signalling. Several recent studies have integrated TF binding site or occupancy data to identify cancer-associated TFs, for example, combining tumour-specific DNA methylation changes in distal enhancers, mRNA sequencing and *cis*-regulatory sequences mediating effects on target genes^71^ or integrating ENCODE TF ChIP-seq profiles with the pancancer TCGA expression data^72^. However, these approaches do not model the relationship between perturbed pathways (for example, from proteomic data) and TF activity, nor do they consider the impact of somatic alterations on gene regulatory models.

Overall, current methods cannot translate the mutational landscape of a tumour into a usable model of affected pathways nor use mutational status to predict accurately response to targeted therapies. Our model is designed to capture the causal flow of information from signalling to TFs to target genes; the association analysis is likely to identify causal impacts of mutations and copy number events, since somatic alterations usually alter TF activity/signalling rather than vice versa (with exception of TFs/signalling pathways involved in DNA repair). Our analysis revealed both known and putative interactions of frequently altered genes with signalling and transcriptional programs in a pancancer context and provides a general strategy for future studies. In cases where a mutation is associated with the altered activity of a targetable TF or (phospho)protein, our analysis may suggest combination therapies.

The method we have presented has several limitations. First, our analysis uses predicted TF binding sites based on existing TF motif databases and restricted to promoter sequences; therefore, the TF motif hit matrix is noisy, incomplete and not context-specific. Indeed, due to the strong correlation structure between RPPA and mRNA expression data, AR models trained with the true motif hit matrix achieve only a modest—albeit significant—improvement in prediction performance over models trained on randomized motif data (Supplementary Table 1). Additionally, since many inferred TF and (phospho)protein activities are correlated, individual genomic aberrations may be associated with many regulators. This multiplicity of inferred effects may be biologically reasonable but complicates interpretation. Another methodological challenge is the need to control for the complex background of genomic aberrations. To do this, we used regularized regression with permutation testing to identify a smaller set of somatic alterations with confident associations. Still, the problem of selecting a few significant covariates from a long candidate list given limited sample size is inherently difficult with no fail-safe solution.

Despite these limitations, we have presented a principled integrative strategy for predicting the context-specific impact of somatic alterations on transcriptional programs and signalling pathways. Moreover, our predictions generalize to independent patient cohorts and validate experimentally in isogenic cancer cell line models. We anticipate that such integrative statistical modelling strategies will be crucial for personalizing cancer therapies.

## Methods

### Data and preprocessing

We downloaded RPPA protein expression data from TCPA (http://bioinformatics.mdanderson.org/main/TCPA:Overview). RPPA protein expression data for the UCS study, RNA-seq gene expression data, somatic mutation data and clinical data were downloaded from TCGA's Firehose data run (https://confluence.broadinstitute.org/display/GDAC/Dashboard-Stddata). GISTIC copy number data was downloaded from TCGA's Firehose analyses run (https://confluence.broadinstitute.org/display/GDAC/Dashboard-Analyses). Only the samples ‘whitelisted' by TCGA for the Pan-Cancer Analysis Working Group were used in the study. For our analysis, we restricted to samples with parallel RNA-seq, RPPA, somatic mutation and GISTIC copy number data (Supplementary Table 14).

Silent mutations were filtered from somatic mutation data. We removed genes that were not identified as significant (*q*<0.05) by the MutSigCV^73^ as well as not present at least ten samples in each cancer type. To determine copy number alteration events, we used the set of discrete copy number calls provided by GISTIC2 (ref. 74). We considered genes to be altered only in samples where they resided either in regions of homozygous loss (−2) or high-level amplification (2) among the set of recurrent copy number alterations. We removed genes that were not identified as significant (*q*<0.001) by the GISTIC2 (ref. 74) as well as not present at least ten samples in each cancer type. Then, we encoded somatic aberrations as being present/absent. The final selected set of binary calls for genomic alterations provided a simplified but informative description of the somatic alterations observed in individual tumours.

Log 10-transformed RNA-seq RSEM gene expression values for each of the 12 cancer types were processed independently to identify the set of 5,000 genes that varied most across samples. Gene expression and protein expression vectors were both mean-centred.

To construct the motif hit matrix, we downloaded the TF binding site predictions (TRANSFAC v.7.4) for all target genes from MSigDB^34^. We removed motifs with similar sets of targets to reduce redundancy. This matrix defined a candidate set of regulatory relationships between TFs and target genes. Further, for each of the 12 cancer types, we filtered TFs that were not expressed in at least 40% of samples (Supplementary Data 1).

We obtained SILAC-based quantitative phosphoproteomic data set of a spontaneously immortalized non-tumorigenic breast epithelial cell line MCF10A along with two isogenic derivatives generated by knock-in of mutant alleles—one bearing the E545K mutation and the other bearing the H1047R mutation of the *PIK3CA* gene—from the originally published Supplementary Data^45^. We used a 1.5-fold cutoff value to designate peptides as having increased phosphorylation and a 0.67-fold for decreased phosphorylation (same thresholds as original publication)^45^. We also obtained human protein microarray-based AKT1 kinase assays from the originally published Supplementary Data^45^.

We obtained RPPA data for uterine corpus endometrioid carcinoma^25^,^75^ and head and neck cancer patients^28^ from the original publications.

### Training the AR models

AR is an algorithm for efficiently solving a regularized bilinear regression problem^15^,^16^, defined here as follows. For a data set of *M* tumour samples profiled using RNA-seq with *N* genes, we let **Y**
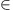
*R*^*N*x*M*^ be the mean-centred log 10 gene expression profiles of tumour samples. Each column of **Y** corresponds to an RNA-seq experiment. We define each gene's TF attributes in a matrix **D**
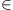
*R*^*N*x*Q*^, where each row represents a gene and each column represent the hit vector for a TF, that is, the bit vector indicating whether there is binding site for the TF in the promoter region of each gene. We define the RPPA attributes of tumour samples as a matrix **P**
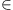
*R*^*M*x*S*^ where each row represents a tumour sample and each column represents the (mean-centred) log RPPA protein expression profile for the tumour sample. We set up a bilinear regression problem to learn the weight matrix **W**
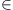
*R*^*Q*x*S*^ on paired of TF signalling protein features:


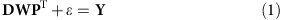


We can transform the system to an equivalent system of equations by reformulating the matrix products as Kronecker products


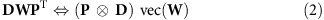


where ⊗ is a Kronecker product and vec(.) is a vectorizing operator that stacks a matrix and produces a vector, yielding a standard (if large-scale) regression problem. Full details and a derivation of the reduced optimization problem are provided elsewhere^16^. We fit the ridge regression model using the SLEP MATLAB package and evaluate performance with 10-fold cross-validation.

Given the (phospho)protein profile of a test tumour sample (centred relative to the mean of the training set), we can right multiply the (phospho)protein expression vector through the trained model to predict the similarity of its expression profile to those of the training tumour samples. To recover a reconstruction of the test gene expression profile from the predicted similarities, we assume that the test expression profile is in the linear span of the training profiles. Then, a simple transformation converts the vector of computed similarities into a predicted gene expression variation profiles^16^. Finally, to infer the (phospho)protein activity in a new sample from the (centred) gene expression profile, we can left multiply through the model via **Y^T^DW** and to infer the TF activities in each sample, we can right-multiply the protein expression profiles through the model by **WP**^T^.

### Significance analysis for TF and (phospho)protein activities

To assess the statistical significance of the inferred (phospho)protein and TF activities obtained from the model via the **Y**^T^**DW** and **WP**^T^ mappings, respectively, we developed an empirical null model as follows. First, we generated random permutations of the gene expression profiles **Y** for each tumour type. For each permuted **Y** response matrix, we trained an AR model using true **D** and **P** input matrices and computed the corresponding inferred TF and (phospho)protein activities via the **Y**^T^**DW** and *WP*^T^ mappings. Using this permutation and model fitting procedure 5000 times, we generated an empirical null model for TF and (phospho)protein activity distribution for each sample. To identify significant regulator activities (**R**), we assessed the nominal *P* value for each sample relative to the empirical null model for the particular regulator (TF/(phospho)protein), and we corrected for multiple hypothesis testing of non-independent hypotheses using the Benjamini–Hochberg–Yekutieli procedure. Then, we reported the significant regulators using an FDR of 0.1 for the largest TCGA studies (BRCA, KIRC; >300 samples), an FDR of 0.15 for mid-size studies (BLCA, COADREAD, HNSC, LUAD, LUSC, UCEC, OV, PRAD; sample size <300 and >100), and an FDR of 0.25 for small studies (GBM, UCS; <100 samples) as our thresholds for significance. Then, we calculated, for each TF/signalling regulator, the frequency over samples where the regulator passed its significant threshold for a given cancer. We used this approach to identify significant regulators in each cancer type to identify the shared and cancer-specific roles TF/(phospho)protein regulators.

### Model impact of genomic aberrations in terms of TF/(phospho)protein activity

We used ridge regression to predict each TF/(phospho)protein regulator's activity (**R**) from genomic aberration profiles and used a permutation test approach to assign significance to ridge regression coefficients. Somatic alterations were simply encoded as present/absent. In the permutation test, the elements of the outcome vector of regulator activities **R** were randomly permuted across samples, and the ridge regression model was fitted using the permuted observations to obtain ridge regression coefficients. By performing 10,000 such permutations, a null distribution of the regression coefficients was generated. The permutation test *P* value was calculated as the proportion of regression coefficients from the null distribution greater than or equal in absolute value to the absolute value of the coefficient fitted to the true (non-permuted) data. Further we corrected for multiple hypothesis testing of non-independent hypotheses using the Benjamini–Hochberg–Yekutieli procedure across all TF/signalling regulators for each cancer, and we multiplied these FDR-adjusted *P* values with the sign of the coefficient from the model to calculate a final regulatory–genomic aberration association score. For downstream analysis, we restricted our analysis to regulators identified as significant in at least 1% of samples in each cancer and genomic aberrations with FDR-corrected *P*<0.15 across regulators from the ridge regression analysis with permutation test.

We also assessed synergistic/antagonistic effects of pairs of genomic aberrations on TF/protein activity by building linear models with interaction terms for each pair of genomic aberrations. We restricted our analysis to pairs of genes that were altered in at least in 20 tumour samples as well as comutated in at least 10 tumour samples. Here the activity of each TF/(phospho)protein regulator (**R**) was a modelled function of somatic aberration pairs *A* and *B*:


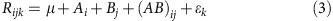


where *A*_*i*_ and *B*_*j*_ represent the main effects of the *i*th and *j*th values of *A* and *B*, respectively, (*AB*)_*ij*_ is the effect of the interaction in that combination, and *ɛ*_*k*_ is the error term of the *k*th observation in that combination. Here the genomic aberrations are binarized (present/absent), so the values are 1 and 0.

We first looked for regulator models for which the coefficient of the interaction term was significant (*P*<0.05). If the interaction term was significant, and if its coefficient was greater than zero and greater than the coefficients of the genomic aberration pair, we assumed this pair of somatic aberrations had a synergistic effect on regulator activity. If the interaction term was significant and its coefficient was less than zero and less than the coefficients of the genomic aberration pair, we assumed this pair of somatic aberrations had an antagonistic effect on regulator activity.

### Survival analyses

We built Cox proportional hazard regression models for regulators that attained significance by our empirical *P* value procedure in at least 5% of samples using (1) their inferred TF activity profiles and (2) their gene expression profiles corresponding to TFs. We used clinical stage as a background factor for BLCA and KIRC and histological subtype as a background factor for UCEC. Overall survival was calculated from the date of initial diagnosis of cancer- to disease-specific death (patients whose vital status is termed dead) and months to last follow-up (for patients who are alive). Further, we evaluated prognostic accuracy of survival models using a log-rank test. We corrected for multiple hypothesis testing of non-independent hypotheses using the FDR procedure across all models in each cancer type. FDR-corrected *P* values of models built from inferred TF activities and actual TF mRNA expression profiles were compared using one-sided paired Wilcoxon's signed-rank test.

For the validation set, we used the TCGA-trained UCEC model to infer TF activity profiles of MDACC (*n*=178) and Bergen (*n*=209) data sets^75^ from their RPPA profiles. Since these data sets do not include serous endometrial carcinoma samples, we build univariate Cox models with just TCGA endometrioid endometrial carcinoma patients. We first identified TFs with univariate Cox *P*<0.05 on the TCGA patients. Then, we predicted the risk for each patient in the validation set and calculating concordance index. We reported the *P* value for the statistical test if the concordance index estimate was different from 0.5.

For visualization, Kaplan–Meier survival analysis was used to show the association of the inferred TF activity with patient survival. For each selected TF and cancer-type combination, each patient's risk was calculated, and patients were ranked in descending order. We designated the top 40% of the patients as the high-risk group and the bottom 40% as the low-risk group.

### Statistical analysis

Statistical tests were performed with the R statistical environment. For population comparisons of inferred TF and (phospho)protein activities, we performed two-tailed Wilcoxon's signed-rank tests and determined the direction of shifts by comparing the mean of two populations.

### Cell lines and transfection

The BRCA cell line, MCF7, which has a PIK3CA E545K mutation, and the targeted correction of the E545K mutation to WT PIK3CA were obtained from the Lauring Lab^46^. The head and neck cancer cell line, Cal27, was obtained from the American Type Culture Collection. The cell lines have been tested negative for mycoplasma contamination. Parental and WT PIK3CA MCF7 and Cal27 were maintained in Dulbecco's modified Eagle's medium (DMEM/DF12) supplemented with 5% foetal bovine serum and 100 U ml^−1^ penicillin and 100 μg ml^−1^ streptomycin. Cal27 cell lines were transfected with pbabe control vector, pbabe WT PIK3CA and pbabe E545K PIK3CA vectors (Addgene) using Lipofectamine 3000 according to the manufacturer's instructions.

### RNA extraction and quantitative real-time PCR

Total RNA was extracted from MCF7 and Cal27 cell lines using an RNA Extraction Kit from Qiagen. cDNA synthesis was performed using iScript from Bio-Rad, according to the manufacturer's instructions. The Applied Biosystems SYBR green mix (Life Technologies) was used to amplify specific genes listed in Supplementary Table 15.

Primers used for mRNA expression were: *ACTR3*, 5′-CATTCCTGTGGCTGAAGGGT-3′ and 5′-ATCGCTGCATGTGGTGTGTA-3′; *FOXP4*, 5′-GACCCTGTGTGAAGACCTGG-3′ and 5′-GTCAGGGGTTTCCAGGATGG-3′; *DDX27*, 5′-TTGGGGAAGGACATCTGTGC-3′ and 5′-CGGATCCGGATGAACTCCTG-3′ *PAPLN*, 5′-AGGTCATCTGTGCCATTGGG-3′ and 5′-TGTAGAAGCCACTGCCCTTG-3′; *PSMB4*, 5′-GACATGCTGGGATCCTACGG-3′ and 5′-CTTTTTCGGTGACAGTGGCG-3′; *WNK1*, 5′-CTTTTTCGGTGACAGTGGCG-3′ and 5′-CTTGGCTGTTCACTGTTGCC-3′; *CDK1*, 5′-ACAGGTCAAGTGGTAGCCATG-3′ and 5′-GGAGTGCCCAAAGCTCTGAA-3′; *CAMKK1*, 5′-CAGGAAGCTATCTGGAGGCG-3′ and 5′-AAGTACTCGAGGCCCAGGAT-3′; *TNFSF10*, 5-CCTCAGAGAGTAGCAGCTCACA-3′ and 5′-CAGAGCCTTTTCATTCTTGGA-3′; *ACTB*, 5′-CGTCTTCCCCTCCATCGT-3′ and 5′-GAAGGTGTGGTGCCAGATTT-3′; *APC*, 5′-CATTTCCAAGAAGAGGGTTTGT-3′ and 5′-GATCAGCAAGAAGCAATGACC-3′; *FBXW11*, 5′-GGCTGCCGTCAATGTAGTAGA-3′ and 5′-GTGCTCGTGCTCCAGACTT-3′; *PPP2R5E*, 5′-GTGTGTATCTAGCCCCCATTTT-3′ and 5′-AAACTCATGATGTATTCATTATTCCAA-3′; *WNT10B*, 5′-ATGCGAATCCACAACAACAG-3′ and 5′-TCCAGCATGTCTTGAACTGG-3′.

### Chromatin immunoprecipitation

MCF7 and Cal27 cell lines were crosslinked with 1% formaldehyde for 10 min at room temperature and quenched with 125 mM glycine for 5 min at room temperature. Cell were lysed and sheared to obtain chromatin fragments of 200–500 bp. Sheared chromatin was incubated overnight with 2 μg of rabbit monoclonal ChIP grade antibody to ELK1 (E277, ab32106; Abcam) as has been previously used by Zhang *et al*.^76^ 2 μg of a goat polyclonal antibody to TCF4 (N-20, sc-8631; Santa Cruz) as has been previously used by Ding *et al*.^77^ and 2 μg of rabbit polyclonal FOXO1 (H-108, sc-11350; Santa Cruz) as has been previously used by Xiong *et al*.^78^ Protein G magnetic beads were used to capture antibody–chromatin association overnight, followed by sequential washes. The antibody bound beads were then reverse crosslinked for 6 h at 65 °C, followed by proteinase K treatment at 55 °C for 1 h. The ChIP DNA was purified using a DNA Purification Kit from Qiagen. The Applied Biosystems SYBR green mix was used to amplify specific regions (Supplementary Table 16).

### Western blot analysis

Cells were lysed and proteins were extracted in RIPA buffer that was supplemented with phosphatase and protease inhibitors. Proteins were separated by SDS–polyacrylamide gel electrophoresis gels and transferred to a PVDF (polyvinylidene difluoride) membrane. Membranes with blocked with 5% bovine serum albumin and probed using specific antibodies. Actin (1:2,000), pAKT (S473) (1:1,000), pS6K (T389) (1:1,000), HA (1:1,000) were all from Cell Signaling Technology (CST).

### Data availability

RPPA protein expression data is available in a public repository from TCPA (http://bioinformatics.mdanderson.org/main/TCPA:Overview). RPPA protein expression data for the UCS study, RNA-seq gene expression data, somatic mutation data and clinical data are available in a public repository from TCGA's Firehose data run (https://confluence.broadinstitute.org/display/GDAC/Dashboard-Stddata). GISTIC copy number data is available in a public repository from TCGA's Firehose analyses run (https://confluence.broadinstitute.org/display/GDAC/Dashboard-Analyses). Only the samples ‘whitelisted' by TCGA for the Pan-Cancer Analysis Working Group were used in the study. For our analysis, we restricted to samples with parallel RNA-seq, RPPA, somatic mutation and GISTIC copy number data Supplementary Table 14 and Supplementary Data 1.

The authors declare that all data supporting the findings of this study are available within the article and its Supplementary Information files or from the corresponding author on reasonable request.

## Additional information

**How to cite this article:** Osmanbeyoglu, H. U *et al*. Pancancer modeling predicts the context-specific impact of somatic mutations on transcriptional programs. *Nat. Commun.* **8**, 14249 doi: 10.1038/ncomms14249 (2017).

**Publisher's note:** Springer Nature remains neutral with regard to jurisdictional claims in published maps and institutional affiliations.

## Supplementary Material

Supplementary Information Supplementary Figures, Supplementary Tables and Supplementary References

Supplementary Data 1 Significance analysis (permutation test) - left tailed and right tailed p-values for each transcription factor (TF) and (phospho) protein activities for each cancer type.

Peer Review File
