# Supplementary material for: Pancancer modelling predicts the context-specific impact of somatic mutations on transcriptional programs
Source: Nat Commun. 2017 Jan 31;8:14249. doi: 10.1038/ncomms14249 (PMC5290314; doi:10.1038/ncomms14249)
Supplement: Supplementary Information — Supplementary Figures, Supplementary Tables and Supplementary References [file ncomms14249-s1.pdf]

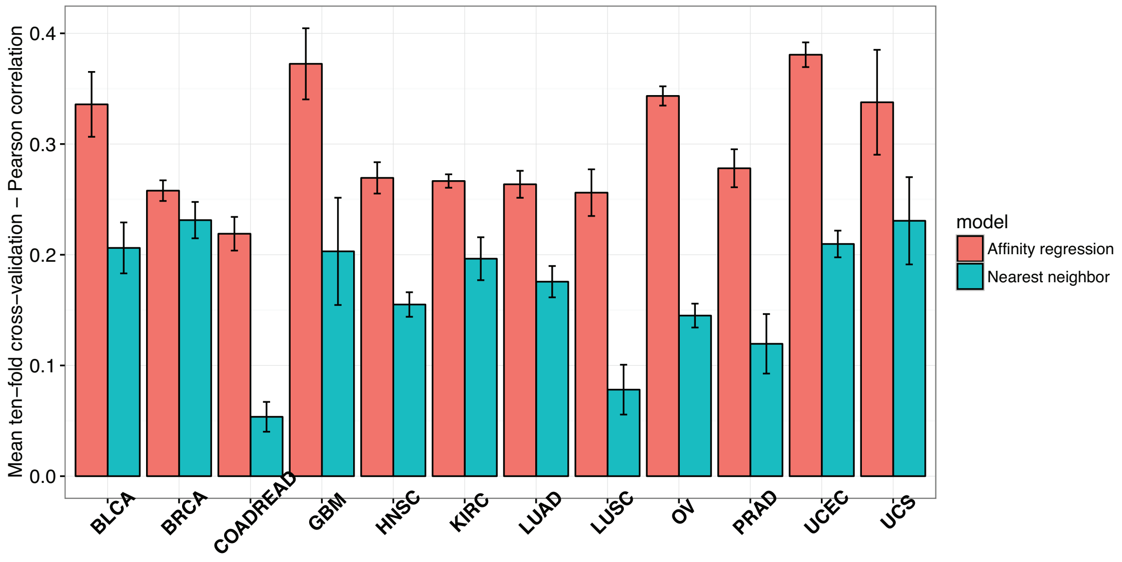

Supplementary Figure 1. Performance of the trained affinity regression models for each TCGA cohort compared to nearest neighbor methods. Mean (± s.d.) ten-fold cross-validation Pearson correlations between predicted and actual gene expression changes relative to a median reference profile using the affinity regression model; nearest neighbor by (phospho)protein expression profile.

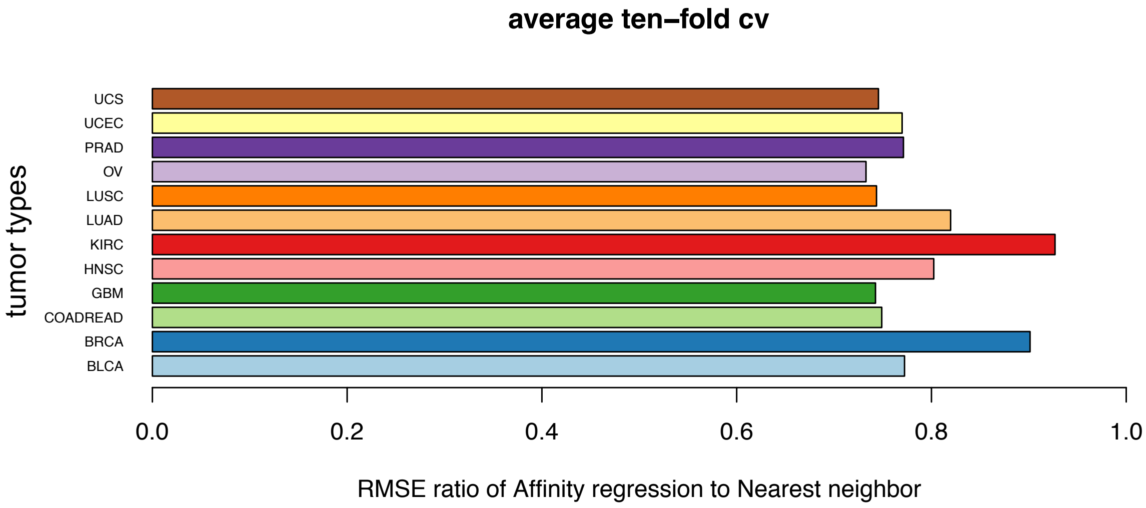

Supplementary Figure 2. Performance of the trained affinity regression models for each TCGA cohort (represented with a different color) compared to nearest neighbor methods. Ratio of mean ten-fold cross-validation root-mean-square error **(**RMSE**)** values between the predicted and actual gene expression changes relative to a median reference profile using the affinity regression model to the nearest neighbor by (phospho)protein expression profile.

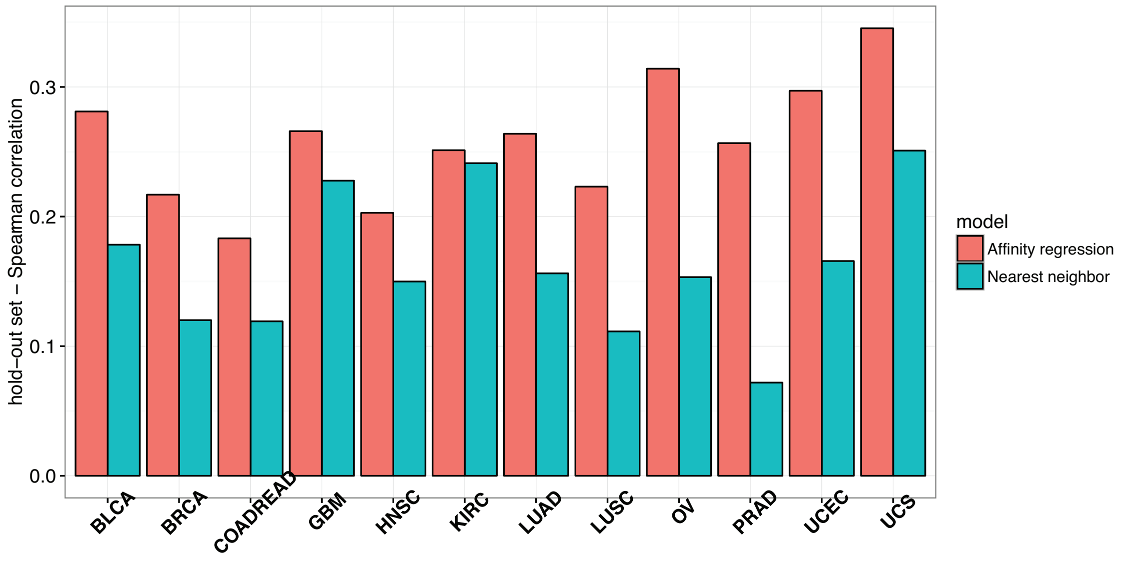

Supplementary Figure 3. Performance of the trained affinity regression models for each TCGA cohort compared to nearest neighbor methods. Mean held-out (test cohort) Spearman correlations between predicted and actual gene expression changes relative to a median reference profile using the affinity regression model; nearest neighbor by (phospho)protein expression profile.

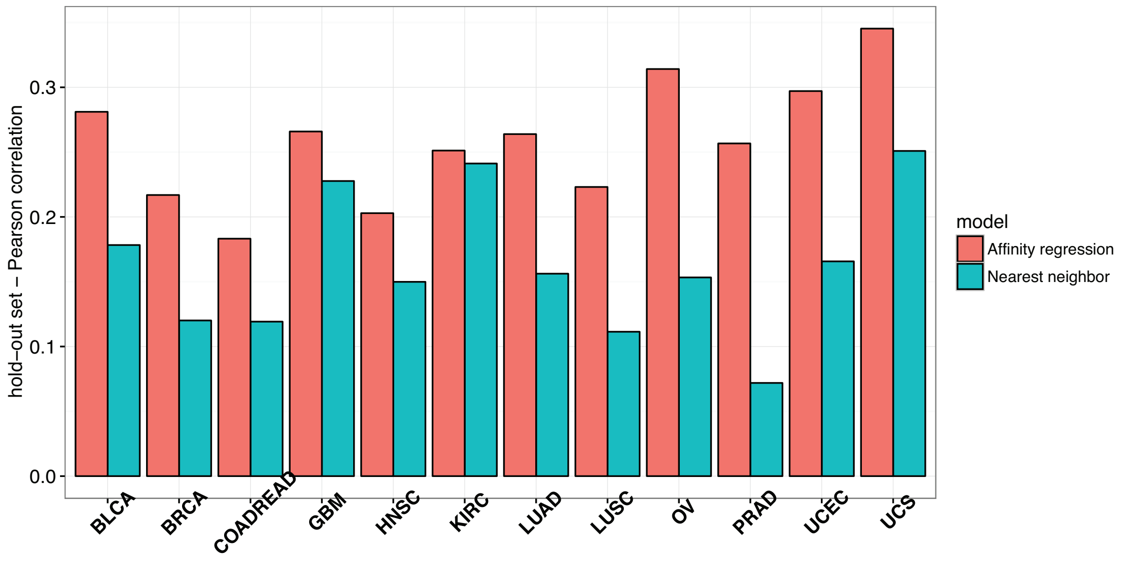

Supplementary Figure 4. Performance of the trained affinity regression models for each TCGA cohort compare to nearest neighbor methods. Mean hold-out (test cohort) Pearson correlations between predicted and actual gene expression changes relative to a median reference profile using the affinity regression model; nearest neighbor by (phospho)protein expression profile.

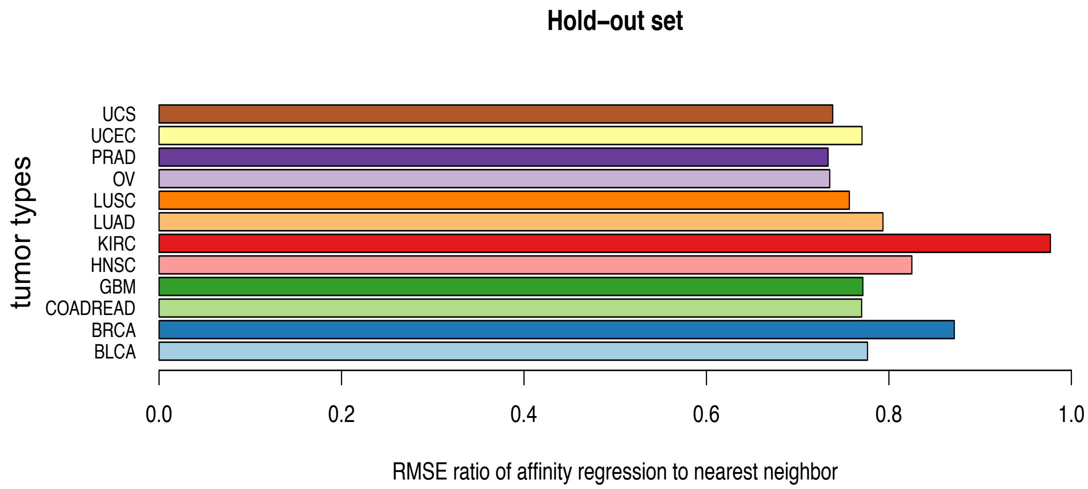

Supplementary Figure 5. Performance of the trained affinity regression models for each TCGA cohort (represented with a different color) compared to nearest neighbor methods. The root-mean-square error (RMSE) ratio between the predicted and actual gene expression changes on held-out test for each TCGA cohort relative to a median reference profile using the affinity regression model to the nearest neighbor by (phospho)protein expression profile.

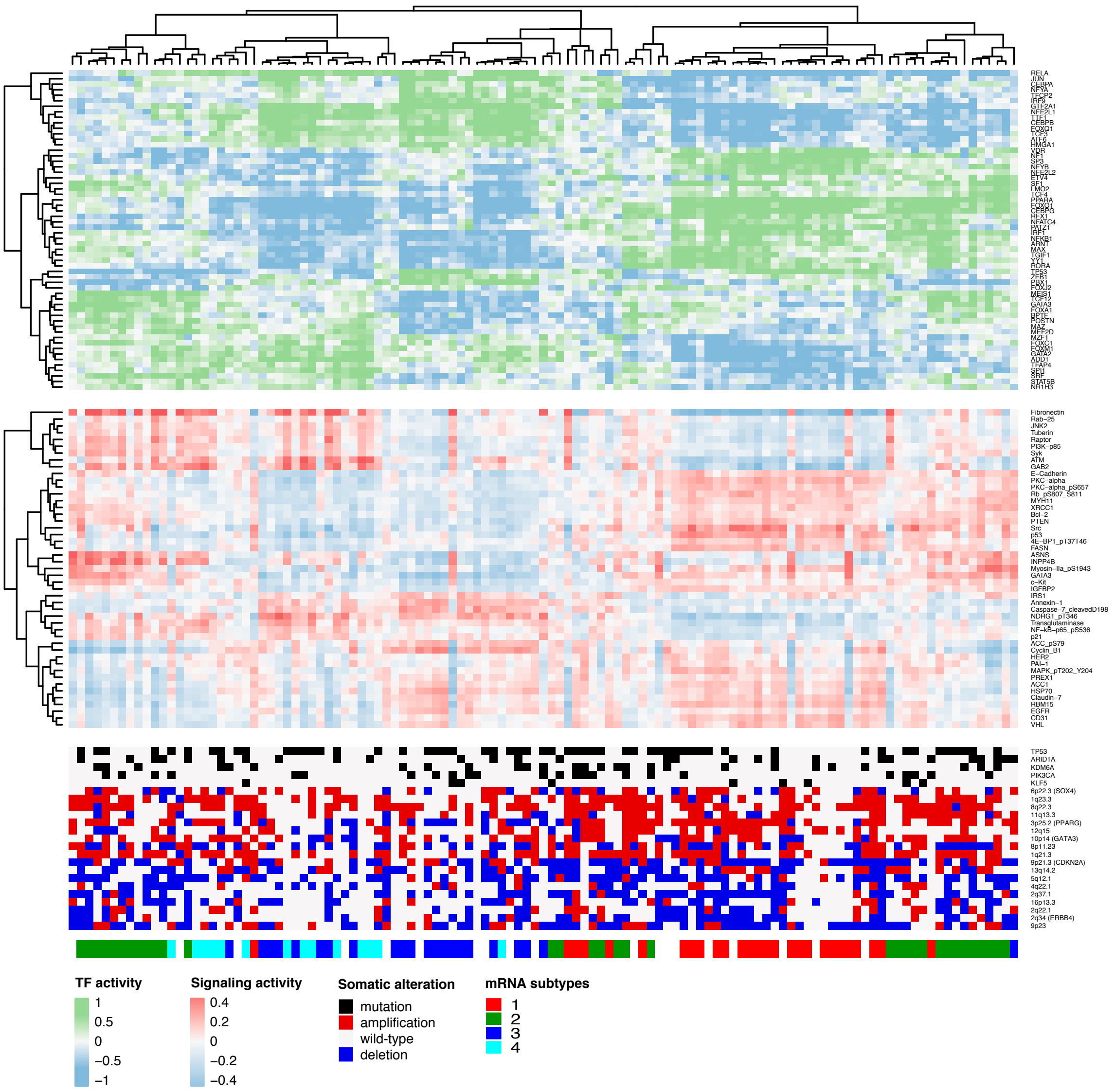

Supplementary Figure 6. TCGA bladder urothelial carcinoma (BLCA) affinity regression model infers sample-specific TF and (phospho)protein activity. The TF/(phospho)protein features with the largest standard deviation across 115 samples are shown in the heat maps. The top panel shows a clustering of tumors by inferred TF activities, together with inferred (phospho)protein activities for the same tumor ordering (middle panel) as derived from the BLCA model. The bottom panel shows the genomic aberration profiles of each tumor as well as gene expression subtypes derived from the corresponding TCGA BLCA study. Patterns of TF activities across tumors are often correlated with patterns of (phospho)protein activities.

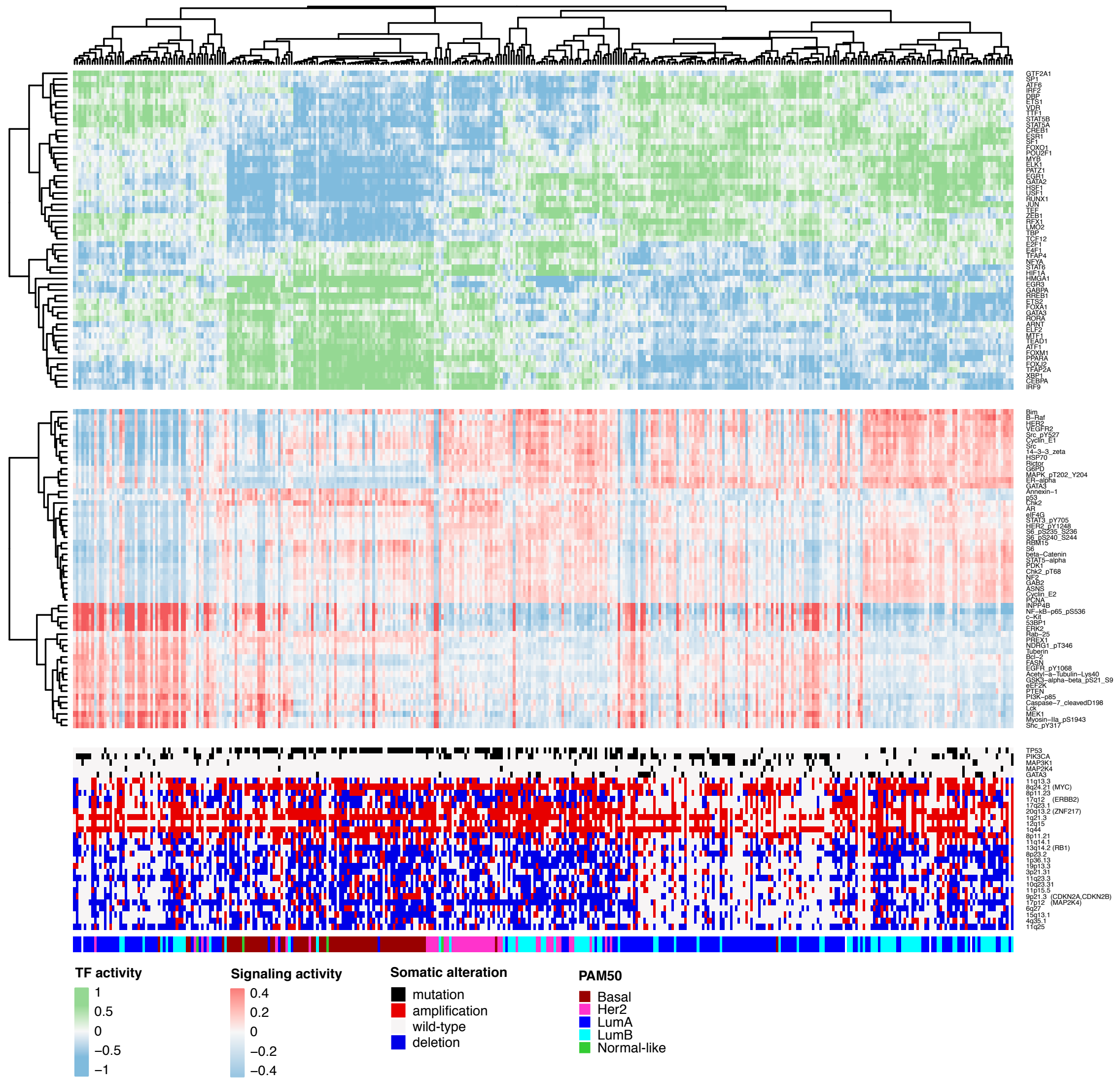

Supplementary Figure 7. TCGA breast cancer (BRCA) affinity regression model infers sample-specific TF and (phospho)protein activity. Unsupervised hierarchical clustering of tumors based on inferred TF activities recovers Basal-like, HER2, and Luminal (LumA and LumB) subtypes. The TF/(phospho)protein features with the largest standard deviation across 368 samples are shown in the heat maps. The top panel shows a clustering of tumors by inferred TF activities, together with inferred (phospho)protein activities for the same tumor ordering (middle panel), as derived from the BRCA model. The bottom panel shows genomic aberration profiles of each tumor as well as Pam50 subtypes derived from the corresponding TCGA BRCA study. Patterns of TF activities across tumors often correlated with patterns of (phospho)protein activities.

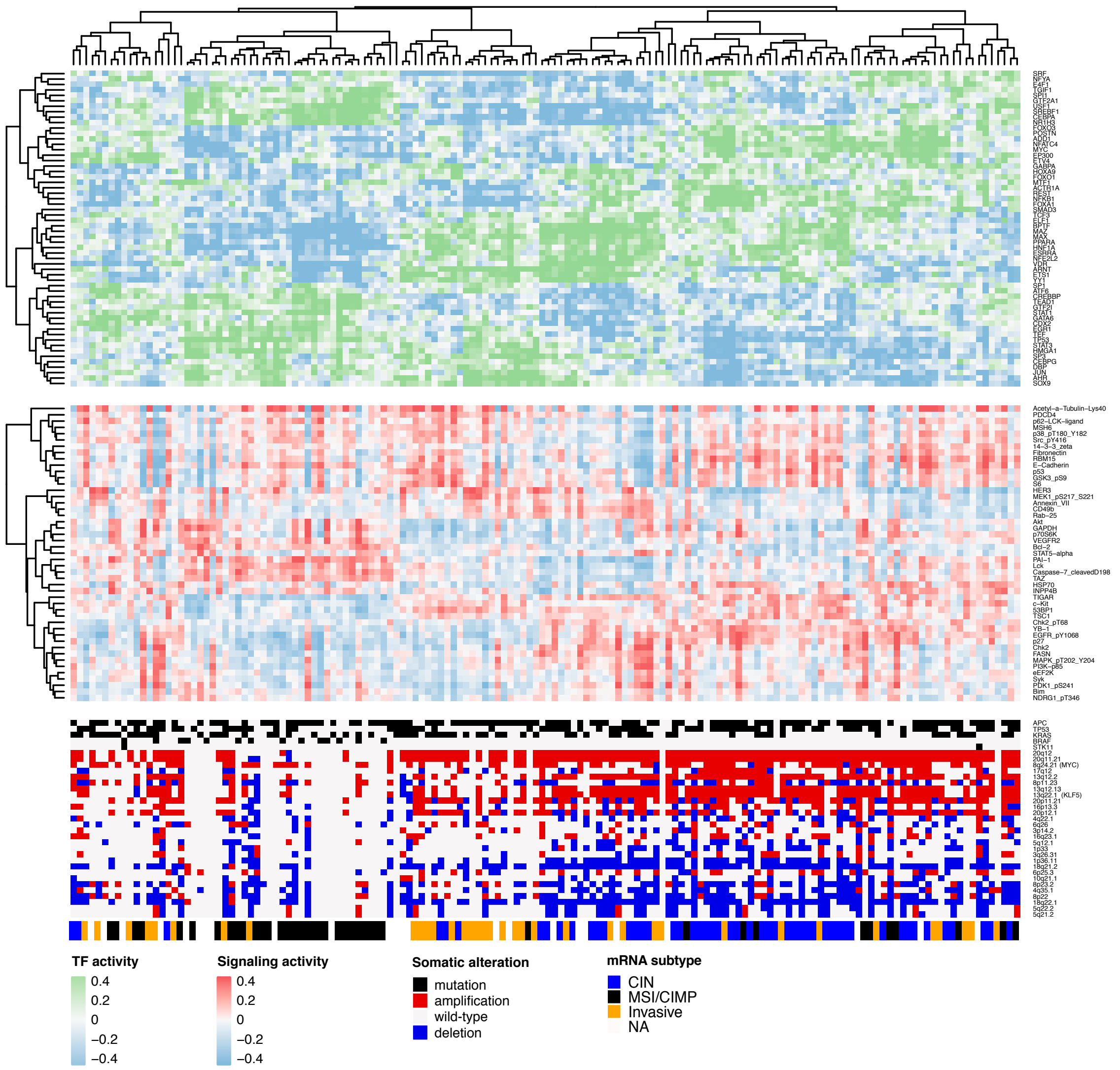

Supplementary Figure 8. TCGA colorectal adenocarcinoma (COADREAD) affinity regression model infers sample-specific TF and (phospho)protein activity. The TF/(phospho)protein features with the largest standard deviation across 150 samples are shown in the heat maps. The top panel shows a clustering of tumors by inferred TF activities, together with inferred (phospho)protein activities for the same tumor ordering (middle panel), as derived from the COADREAD model. The bottom panel shows genomic aberration profiles of each tumor as well as gene expression subtypes derived from the corresponding TCGA COADREAD study. Patterns of TF activities across tumors often correlated with patterns of signaling protein activities, and both TF and (phospho)protein activity profiles also correlated with genomic aberration status.

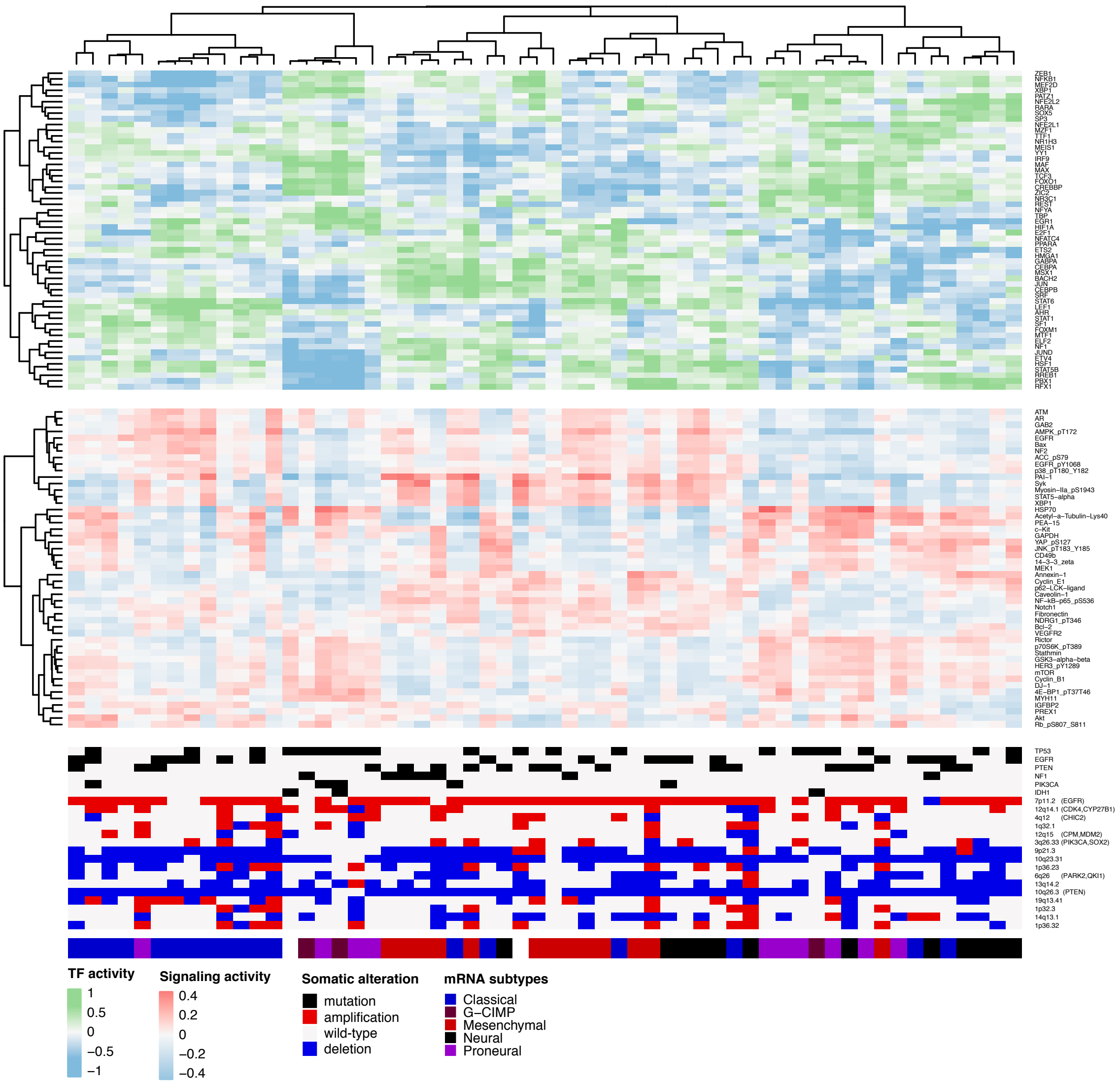

Supplementary Figure 9. TCGA glioblastoma multiforme (GBM) affinity regression model infers sample-specific TF and (phospho)protein activity. The TF/(phospho)protein features with the largest standard deviation across 58 samples are shown in the heat maps. The top panel shows a clustering of tumors by inferred TF activities, together with inferred (phospho)protein activities for the same tumor ordering (middle panel), as derived from the GBM model. The bottom panel shows genomic aberration profiles of each tumor as well as gene expression subtypes derived from the corresponding TCGA GBM study. Patterns of TF activities across tumors often correlated with patterns of (phospho)protein activities.

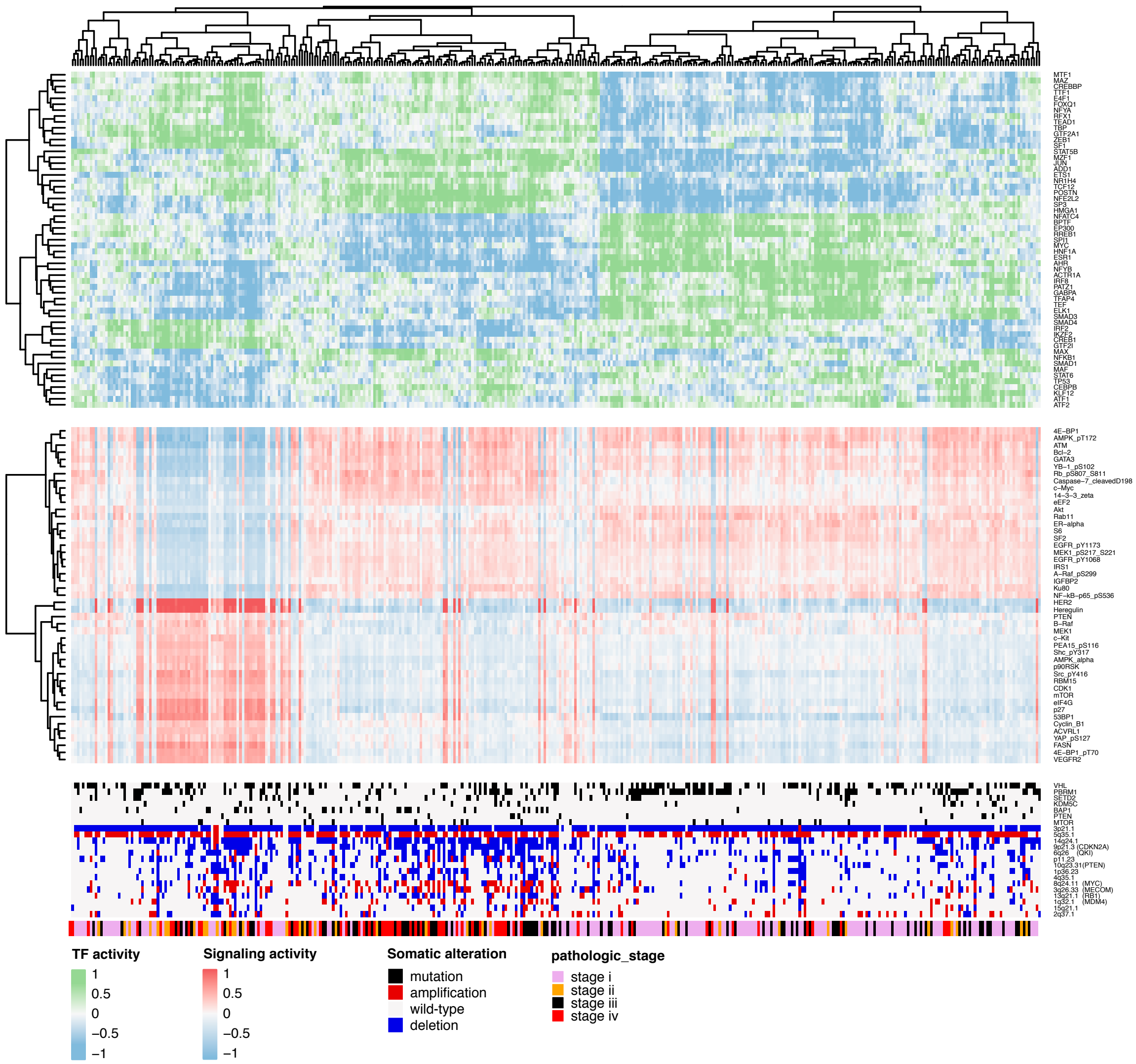

Supplementary Figure 10. TCGA kidney renal cell clear carcinoma (KIRC) affinity regression model infers sample-specific TF and (phospho)protein activity. The TF/(phospho)protein features with the largest standard deviation across 376 samples are shown in the heat maps. The top panel shows a clustering of tumors by inferred TF activities, together with inferred (phospho)protein activities for the same tumor ordering (middle panel), as derived from the KIRC model. The bottom panel shows genomic aberration profiles of each tumor as well as pathological stage from the corresponding TCGA KIRC study. Patterns of TF activities across tumors often correlated with patterns of (phospho)protein activities.

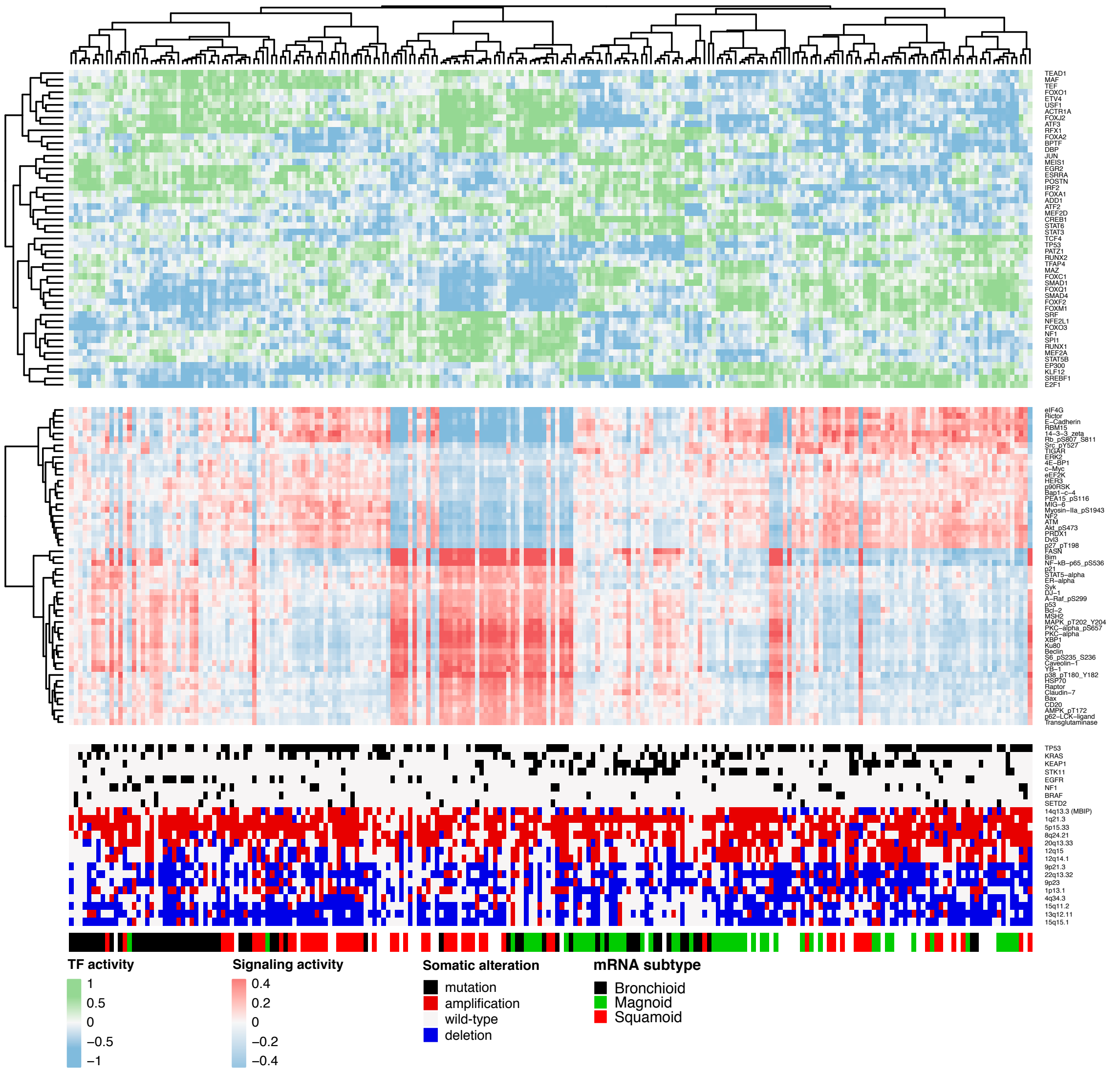

Supplementary Figure 11. TCGA lung adenocarcinoma (LUAD) affinity regression model infers sample-specific TF and (phospho)protein activity. The TF/(phospho)protein features with the largest standard deviation across 216 samples are shown in the heat maps. The top panel shows a clustering of tumors by inferred TF activities, together with inferred (phospho)protein activities for the same tumor ordering (middle panel), as derived from the LUAD model. The bottom panel shows genomic aberration profiles of each tumor as well as gene expression (mRNA) subtypes derived from the corresponding TCGA LUAD study. Patterns of TF activities across tumors often correlated with patterns of (phospho)protein activities.

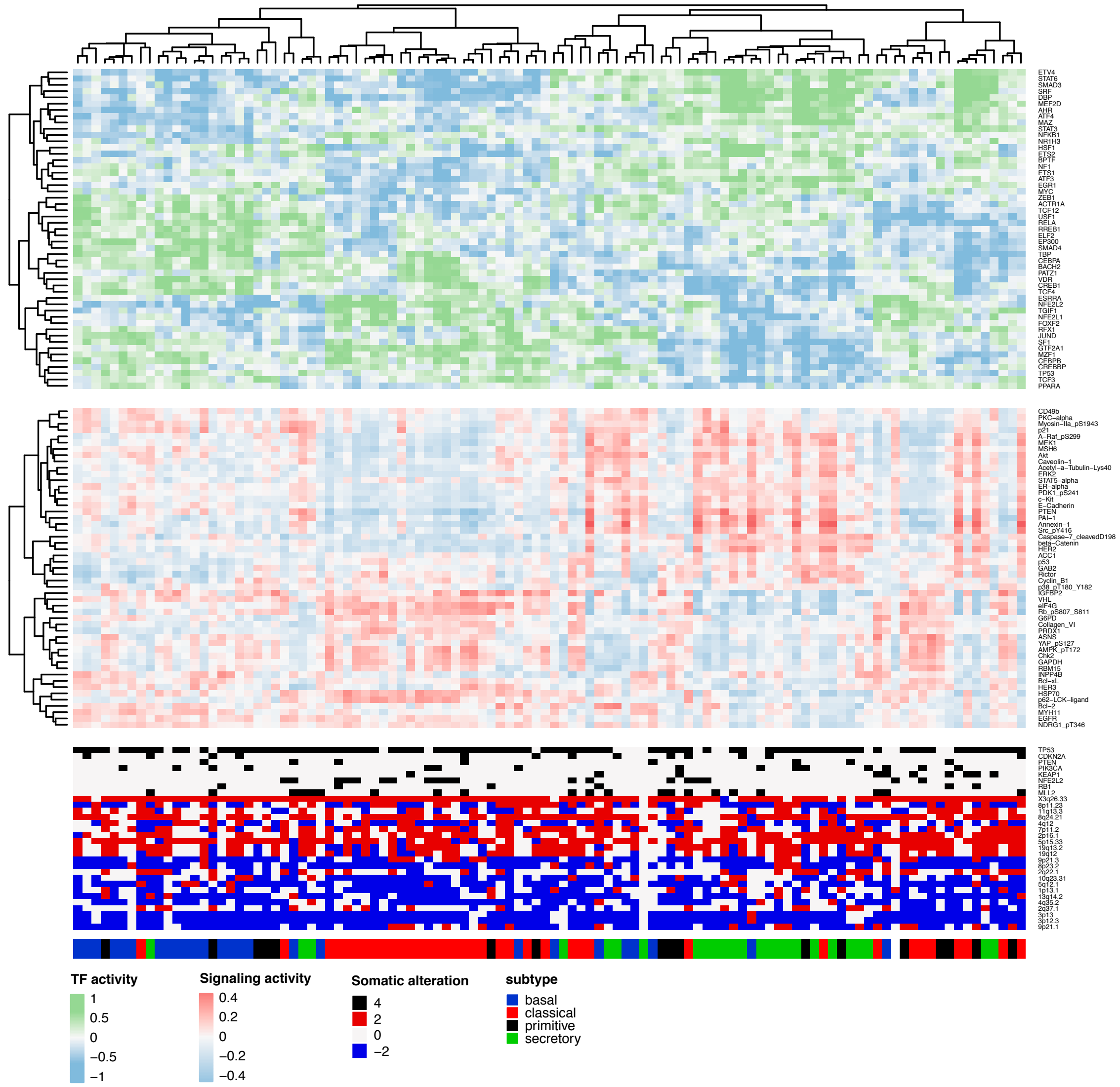

Supplementary Figure 12. TCGA lung squamous cell carcinoma (LUSC) affinity regression model infers sample-specific TF and (phospho)protein activity. The TF/(phospho)protein features with the largest standard deviation across 216 samples are shown in the heat maps. The top panel shows a clustering of tumors by inferred TF activities, together with inferred (phospho)protein activities for the same tumor ordering (middle panel), as derived from the LUSC model. The bottom panel shows genomic aberration profiles of each tumor as well as gene expression subtypes derived from the corresponding TCGA LUSC study. Patterns of TF activities across tumors often correlated with patterns of (phospho)protein activities.

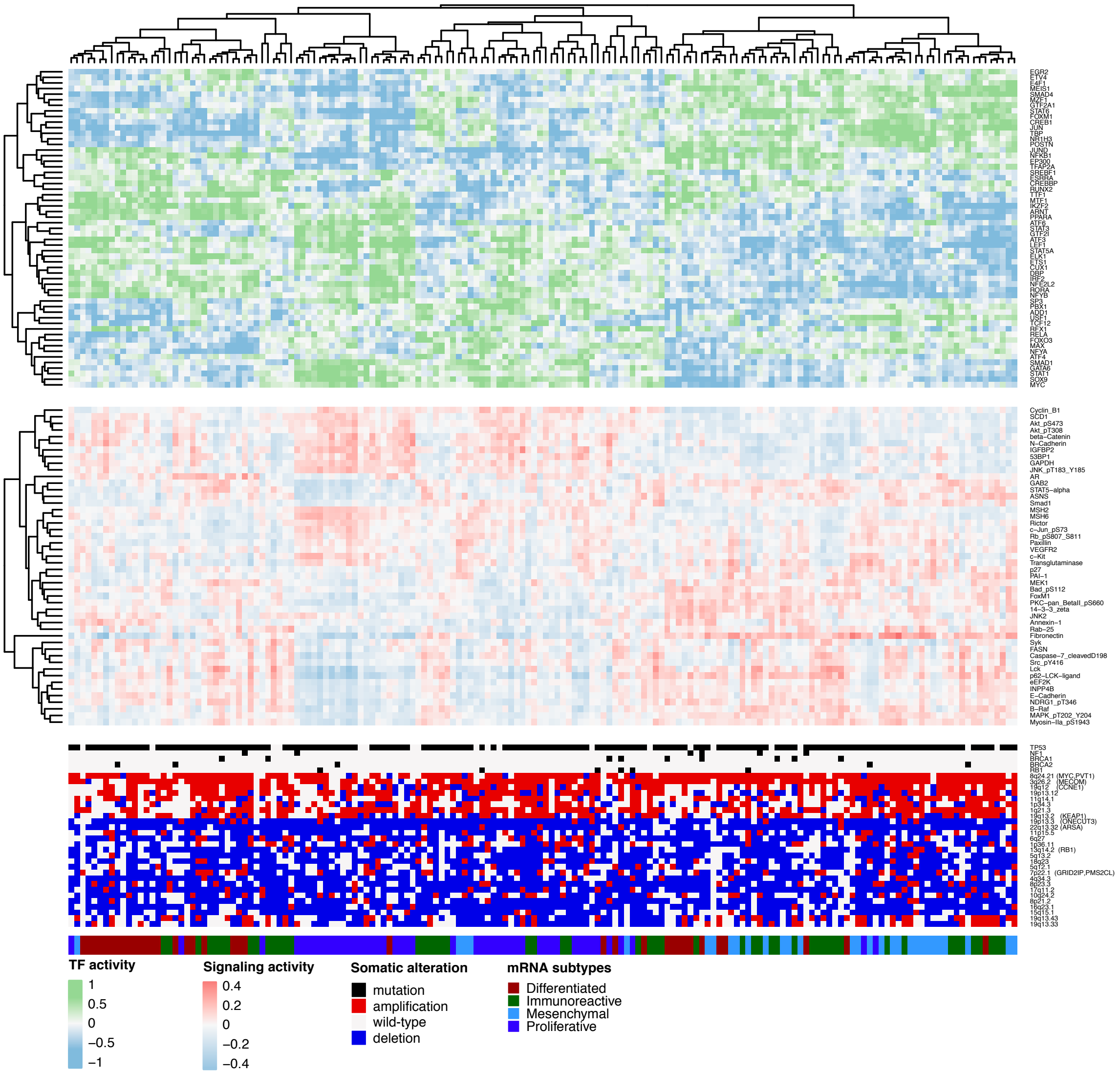

Supplementary Figure 13. TCGA ovarian carcinoma (OV) affinity regression model infers sample-specific TF and (phospho)protein activity. The TF/(phospho)protein features with the largest standard deviation across 164 samples are shown in the heat maps. The top panel shows a clustering of tumors by inferred TF activities, together with inferred (phospho)protein activities for the same tumor ordering (middle panel), as derived from the OV model. The bottom panel shows genomic aberration profiles of each tumor as well as gene expression subtypes derived from the corresponding TCGA OV study. Patterns of TF activities across tumors often correlated with patterns of (phospho)protein activities.

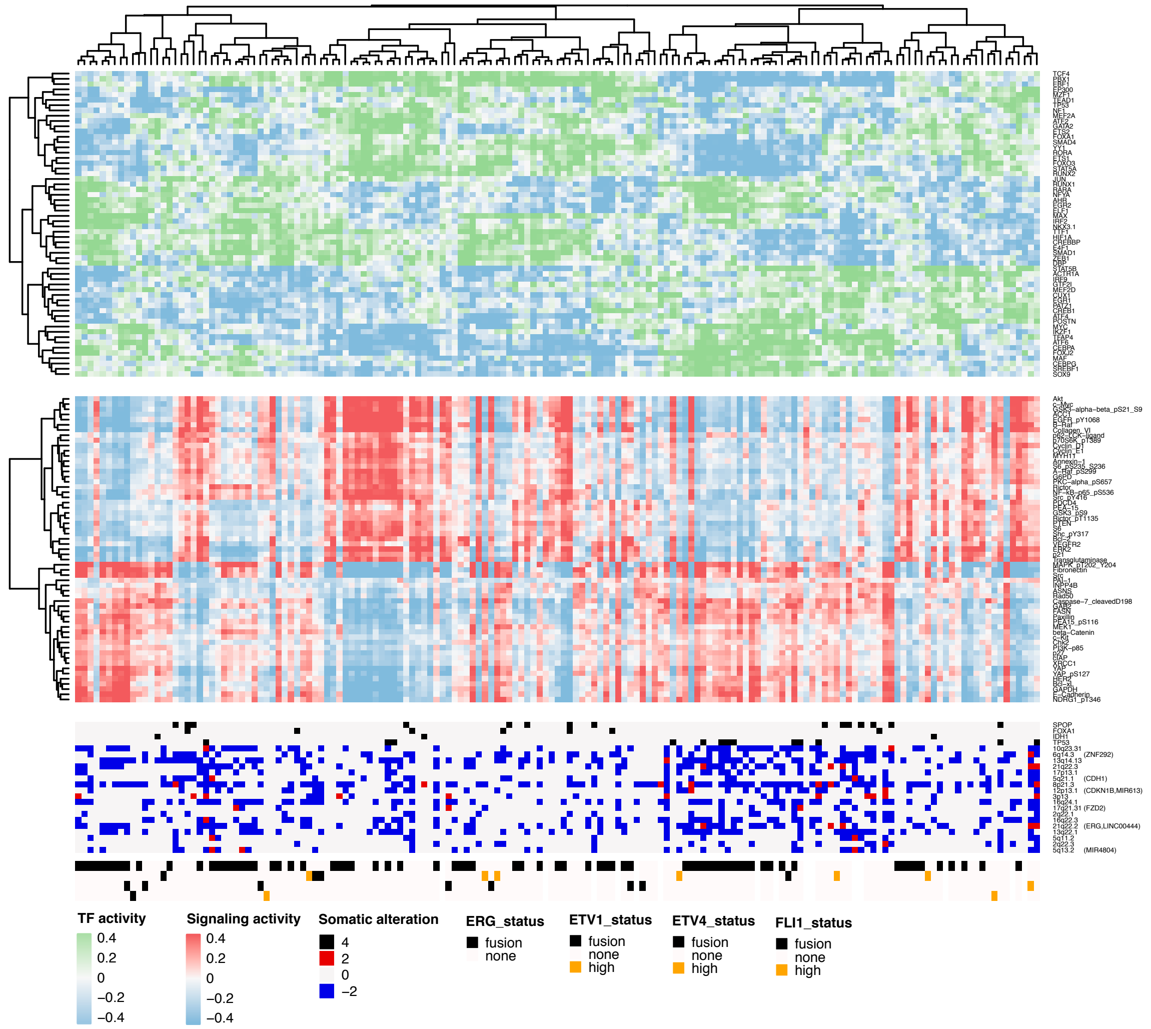

Supplementary Figure 14. TCGA prostate cancer (PRAD) affinity regression model infers sample-specific TF and (phospho)protein activity. The TF/(phospho)protein features with the largest standard deviation across 159 samples are shown in the heat maps. The top panel shows a clustering of tumors by inferred TF activities, together with inferred (phospho)protein activities for the same tumor ordering (middle panel), as derived from the PRAD model. The bottom panel shows genomic aberration profiles of each tumor as well as ERG/ETV1/ETV4/FLI1 fusion status derived from the corresponding TCGA PRAD study. Patterns of TF activities across tumors often correlated with patterns of (phospho)protein activities.

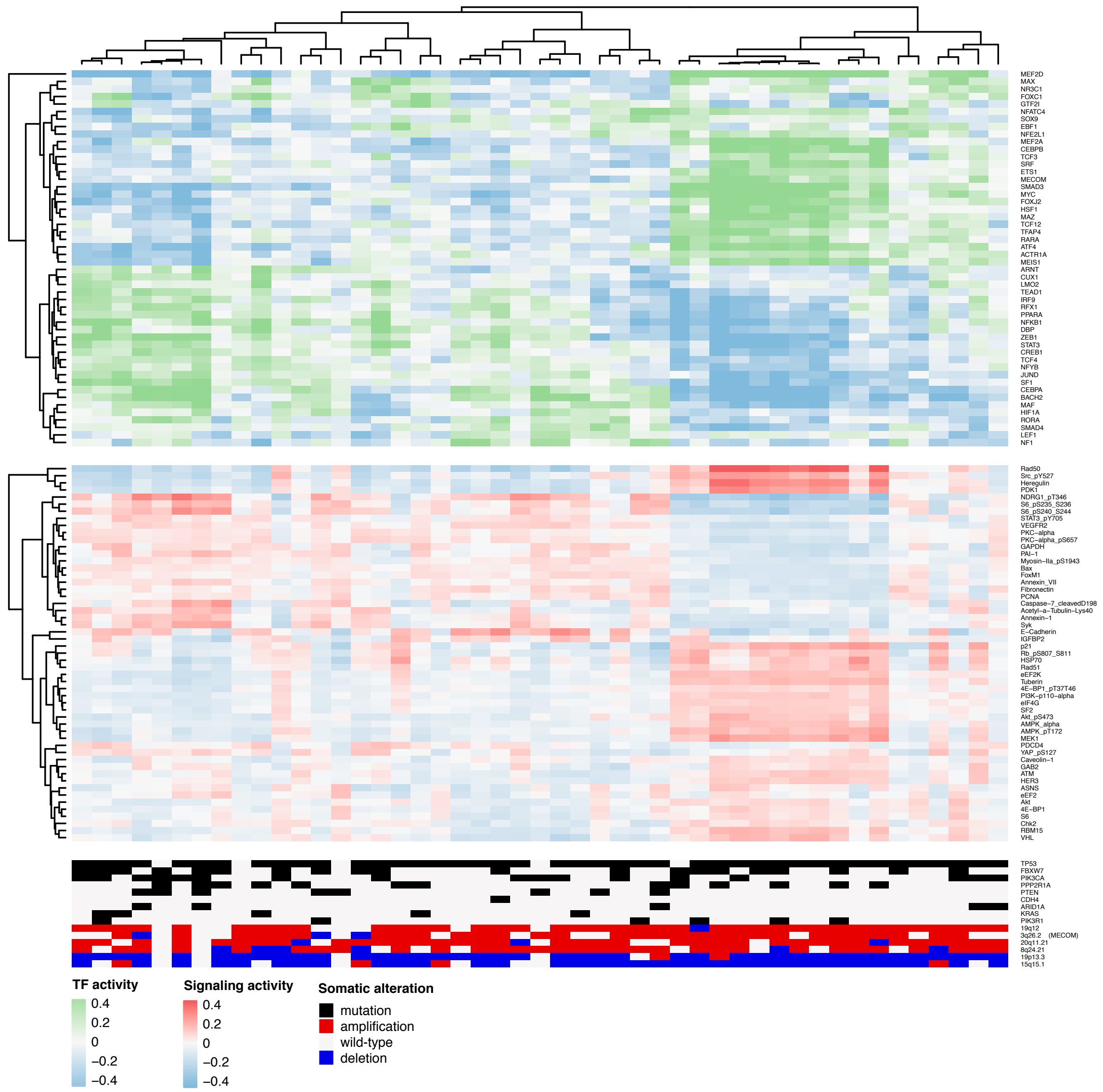

Supplementary Figure 15. TCGA uterine carcinosarcoma (UCS) affinity regression model infers sample-specific TF and (phospho)protein activity. The TF/(phospho)protein features with the largest standard deviation across 47 samples are shown in the heat maps. The top panel shows a clustering of tumors by inferred TF activities, together with inferred (phospho)protein activities for the same tumor ordering (middle panel), as derived from the UCS model. The bottom panel shows genomic aberration profiles of each tumor from the corresponding TCGA UCS study. Patterns of TF activities across tumors often correlated with patterns of (phospho)protein activities.

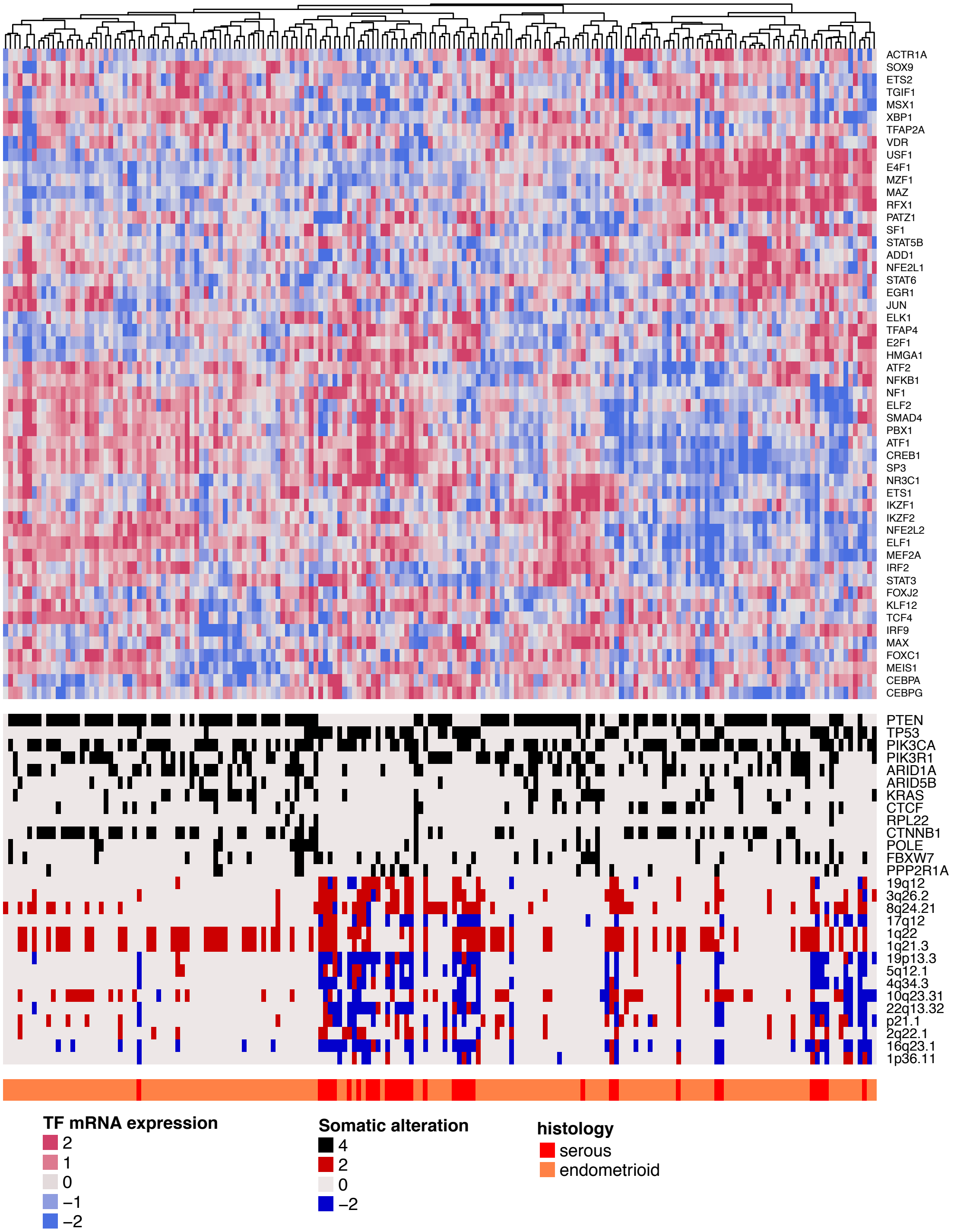

Supplementary Figure 16. Unsupervised hierarchical clustering of tumors based on TCGA endometrial carcinoma (UCEC) TF mRNA expression for 183 samples.

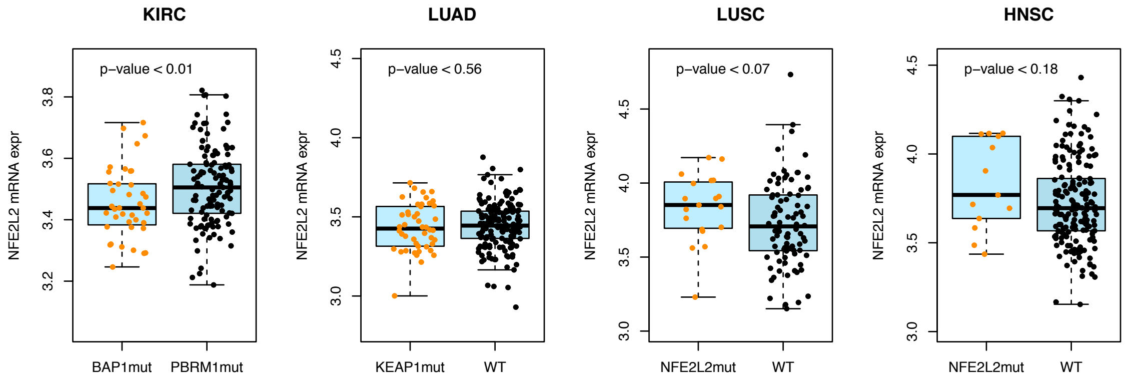

Supplementary Figure 17. NFE2L2 mRNA expression in TCGA KIRC (n=376), LUAD (n=216), LUSC (106), and HNSC (n=194) studies and impact of mutations. In the KIRC study, tumors with mutant *BAP1* have significantly higher NFE2L2 mRNA gene expression than mutant PBRM1 tumors ($P < 0.01$, Wilcoxon rank sum test). The distinction is more significant using inferred NFE2L2 TF activity (**Fig. 4c**). Tumors with mutant *KEAP1*/*NFE2L2* did not have significantly higher mRNA expression of NFE2L2 (a substrate targeted by KEAP1) than wildtype tumors in the LUAD study ($P < 0.56$, Wilcoxon rank sum test), LUSC study ($P < 0.07$, Wilcoxon rank sum test) and HNSC study ($P < 0.18$, Wilcoxon rank sum test). However, the association of mutant *KEAP1*/*NFE2L2* with inferred transcription activity of NFE2L2 is significant in all cases (**Fig. 4c**).

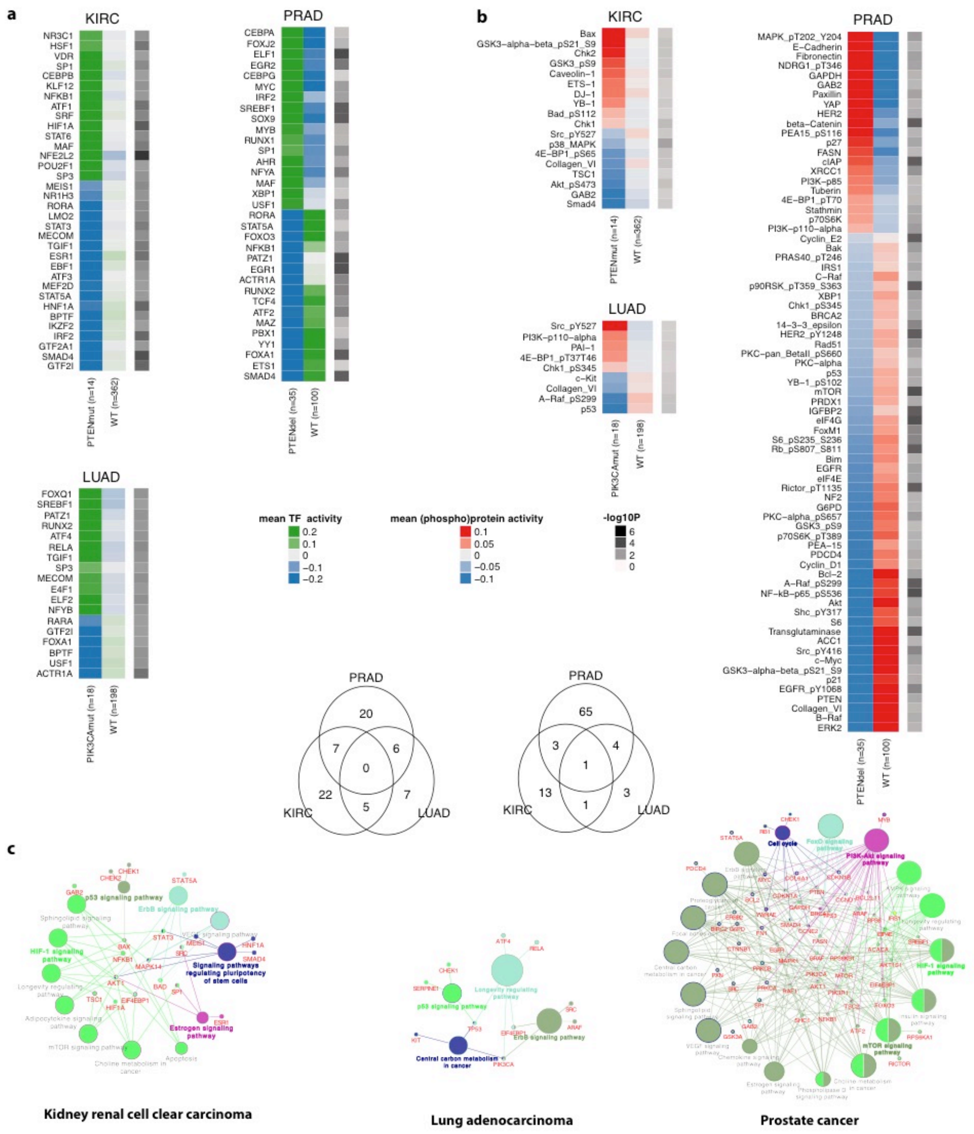

Supplementary Figure 18. Somatic aberrations in the PI3K pathway dysregulate cancer-specific TFs

(a) Heat maps of average TF activity in *PIK3CA* or *PTEN* mutant and wildtype tumors in the TCGA kidney renal cell clear carcinoma (KIRC, n=276), lung adenocarcinoma (LUAD, n=216), and prostate cancer (PRAD, n=159) studies. Boldface TF/protein label indicates association across multiple cancers. **(b)** Heat maps of average protein activity in *PIK3CA* or *PTEN* deletion (PRAD) and wildtype tumors. The significance level of deleted vs. mutant differential TF and protein activity is indicated by the sidebars for each heat map. Venn diagrams showing the number of regulators associated with somatic aberration status across KIRC, LUAD, and PRAD studies. A number of associated signaling regulators are common for KIRC, LUAD and PRAD, while there are no common TF regulators associated with the somatic aberration status across all these cancers. **(c)** A functionally grouped network of enriched categories was generated for TFs associated with *PIK3CA*/*PTEN* mutations using KEGG pathway terms related to cancer as nodes and linked using ClueGO[1] analysis. Only the most significant terms in the group are labeled. Functionally related groups partially overlap.

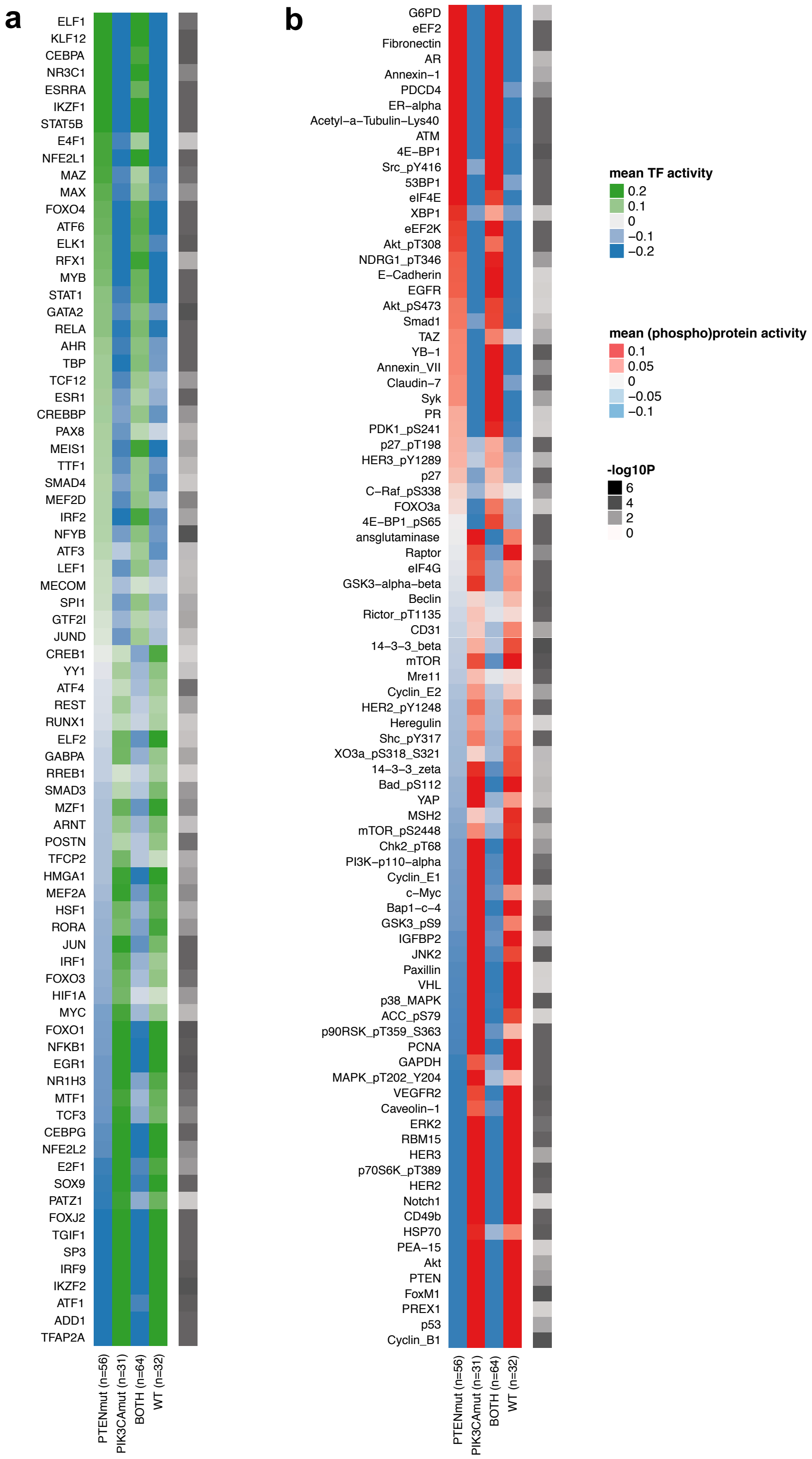

Supplementary Figure 19. (a) Heatmap of average TF activity in *PTEN* mutant, *PIK3CA* mutant, *PTEN-PIK3CA* mutant and wildtype for *PTEN/PIK3CA* tumors in uterine corpus endometrioid carcinoma (UCEC, n=183) studies. **(b)** Heat maps of average (phospho)protein activity in *PTEN* mutant, *PIK3CA* mutant, *PTEN-PIK3CA* mutant and wildtype for *PTEN/PIK3CA*. The significance level of differential TF and protein activity in mutant vs. wildtype tumors is indicated by the sidebars for each heat map.

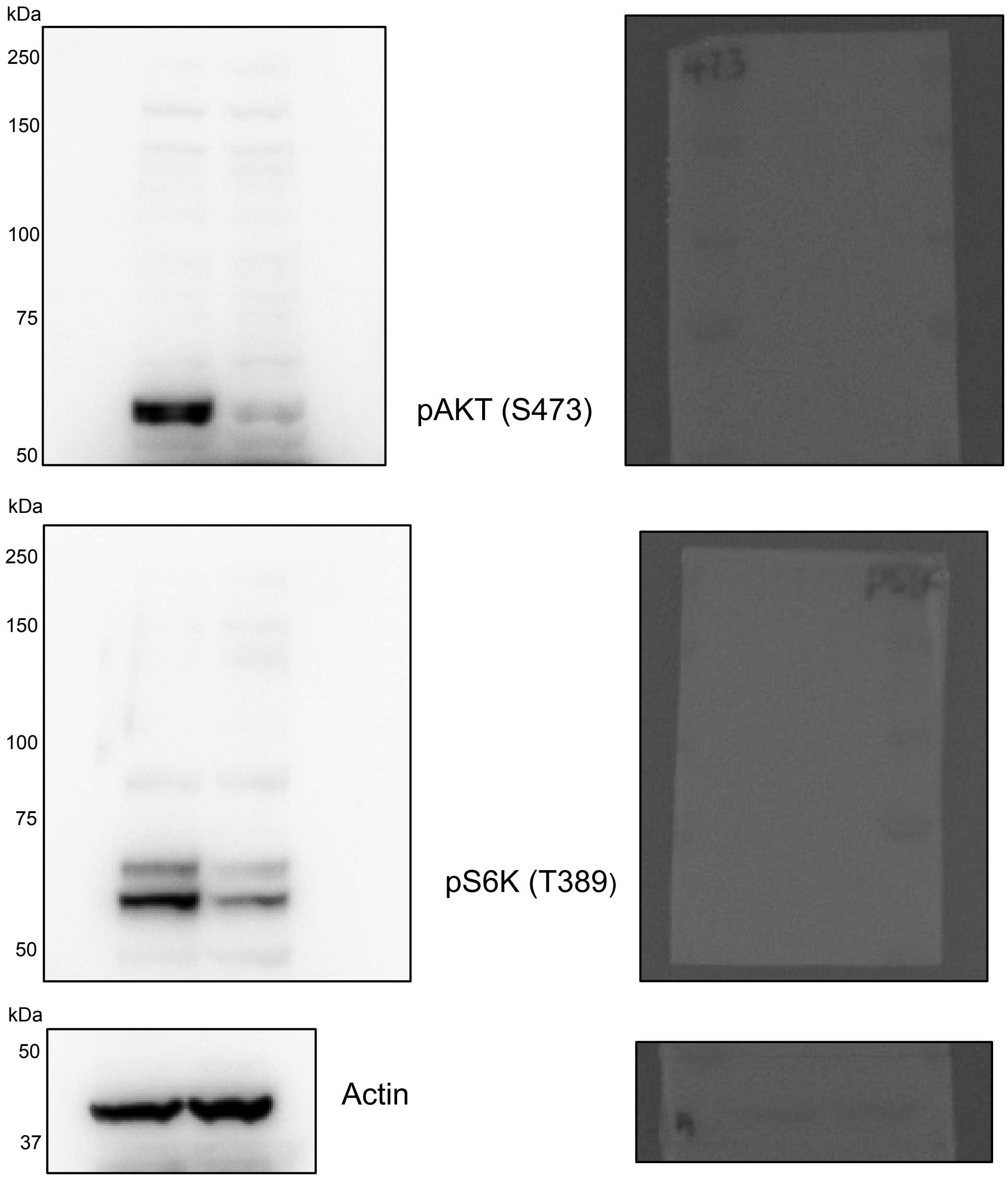

Supplementary Figure 20. Western blot analysis of pAKT (S473), pS6K (T389) and actin in parental MCF7 cells that carry the *PIK3CA* E545K mutation and in 'corrected' wild type (WT) *PIK3CA* cells.

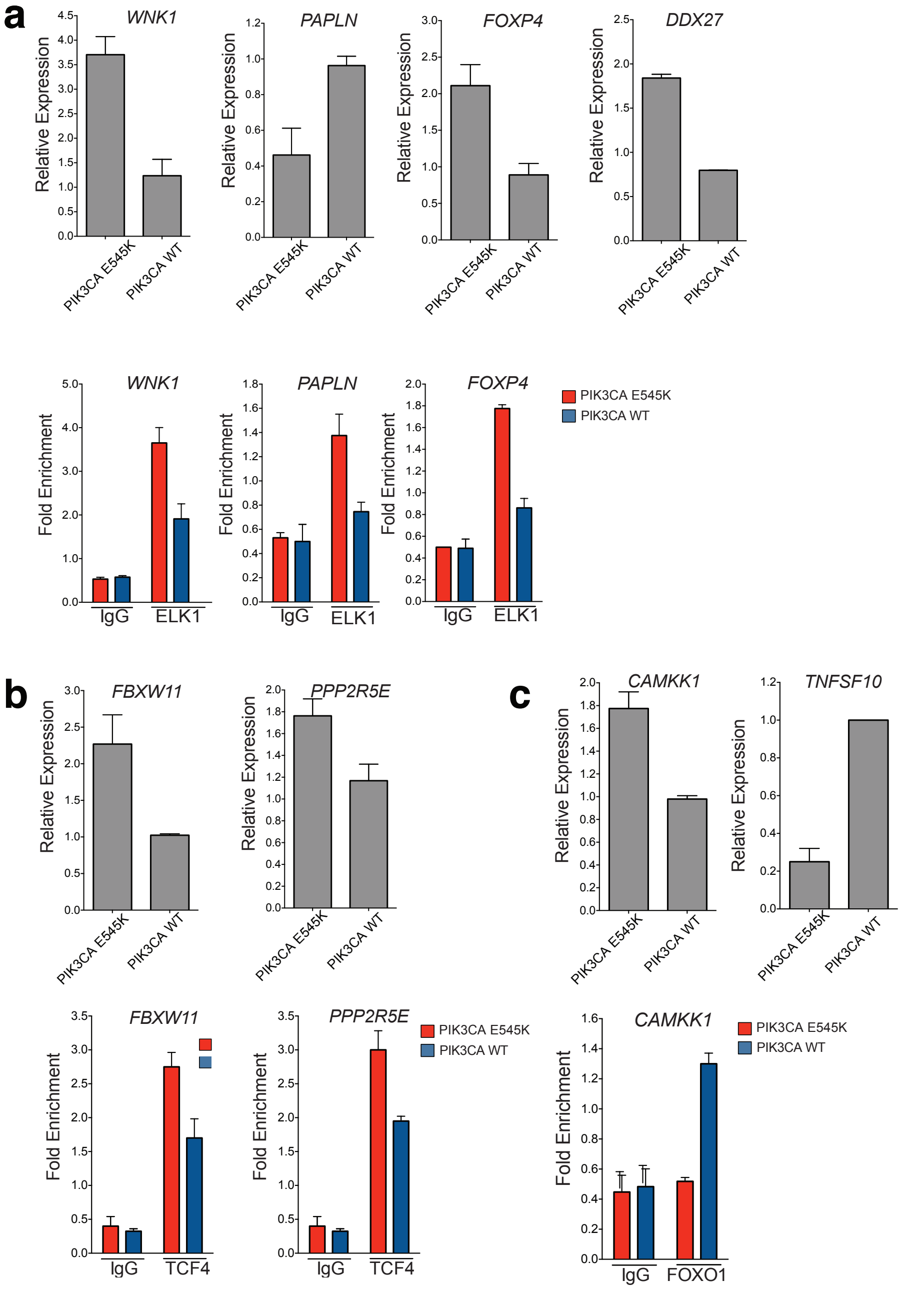

Supplementary Figure 21. (a) ELK1 activity in PI3Kalpha mutant cells: WNK1, PAPLN, FOXP4 and DDX27 mRNA expression in parental *PIK3CA* mutant and *PIK3CA* WT cells. ChIP assays with control IgG or ELK1 antibodies for WNK1, PAPLN, and FOXP4 in parental or WT MCF7 cells. The data are presented as fold-enrichment relative to the actin control gene region (mean ± s.d., n=3 independent experiments). **(b)** TCF4 activity in PI3Kalpha mutant cells: FBXW11, and PPP2R5E mRNA expression in parental and WT cells. ChIP assays with control IgG or TCF4 antibodies for FBXW11, and PPP2R5E in parental or WT MCF7 cells. The data are presented as fold-enrichment relative to the actin control gene region (mean ± s.d., n=3 independent experiments). **(c)** FOXO1 activity in PI3Kalpha mutant cells: CAMKK1, and TNFSF10 mRNA expression in parental and wild type (WT) MCF7 cells. Parental and WT MCF7 cells were subjected to ChIP assays with control IgG or FOXO1 antibodies. The data are presented as fold-enrichment relative to the actin control gene region (mean ± s.d., n=3 independent experiments).

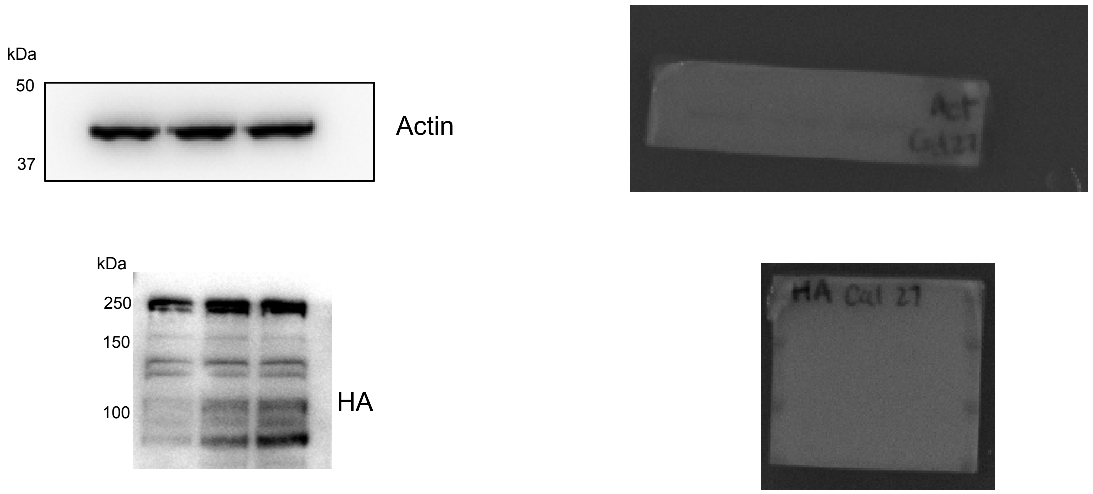

Supplementary Figure 22. Transfected vector control, wild type (WT) *PIK3CA* or *PIK3CA* E545K Cal27 cells were subjected to Western blots with HA and Actin antibodies after 48 hr of transfection.

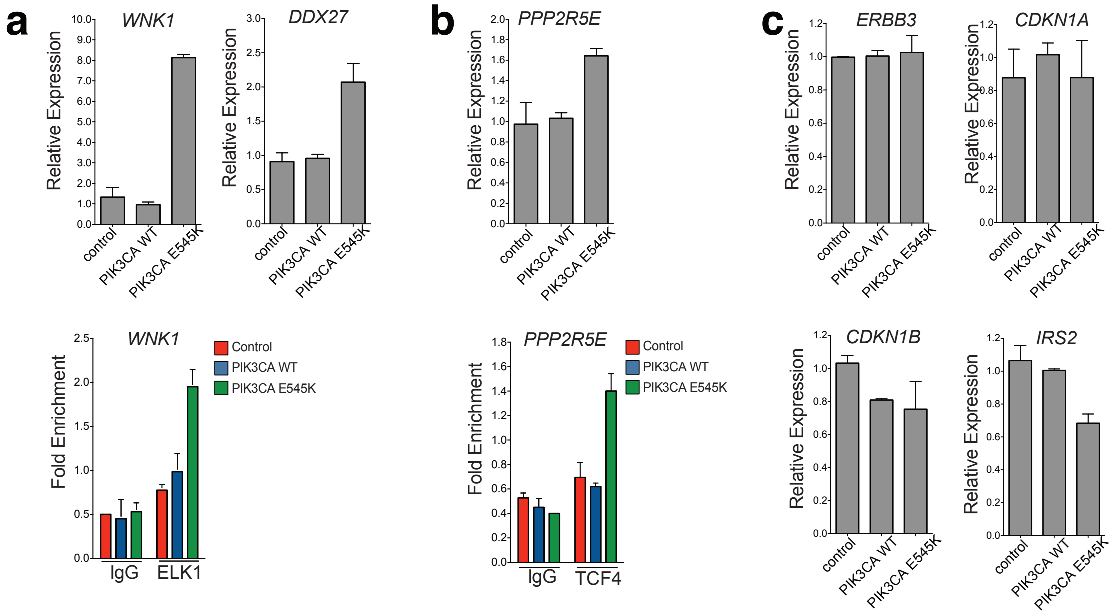

Supplementary Figure 23. Transfected vector control, wild type (WT) *PIK3CA* or *PIK3CA* E545K Cal27 cells. **(a)** WNK1 and DDX27 mRNA expression in control, WT and PIK3CA E545K cells. ChIP assays with control IgG or ELK1 antibodies in control, WT and PIK3CA E545K cells (mean ± s.d., n=3 independent experiments). **(b)** PPP2R5E mRNA expression in control, PIK3CA WT and PIK3CA E545K transfected Cal27 cells. ChIP assays with control IgG or TCF4 antibodies in control, *PIK3CA* WT and *PIK3CA* E545K transfected Cal27 cells (mean ± s.d., n=3 independent experiments). **(c)** ERBB3, CDKN1A, CDKN1B and IRS2 mRNA expression in control, PIK3CA WT and PIK3CA E545K transfected Cal27 cells (mean ± s.d., n=3 independent experiments).

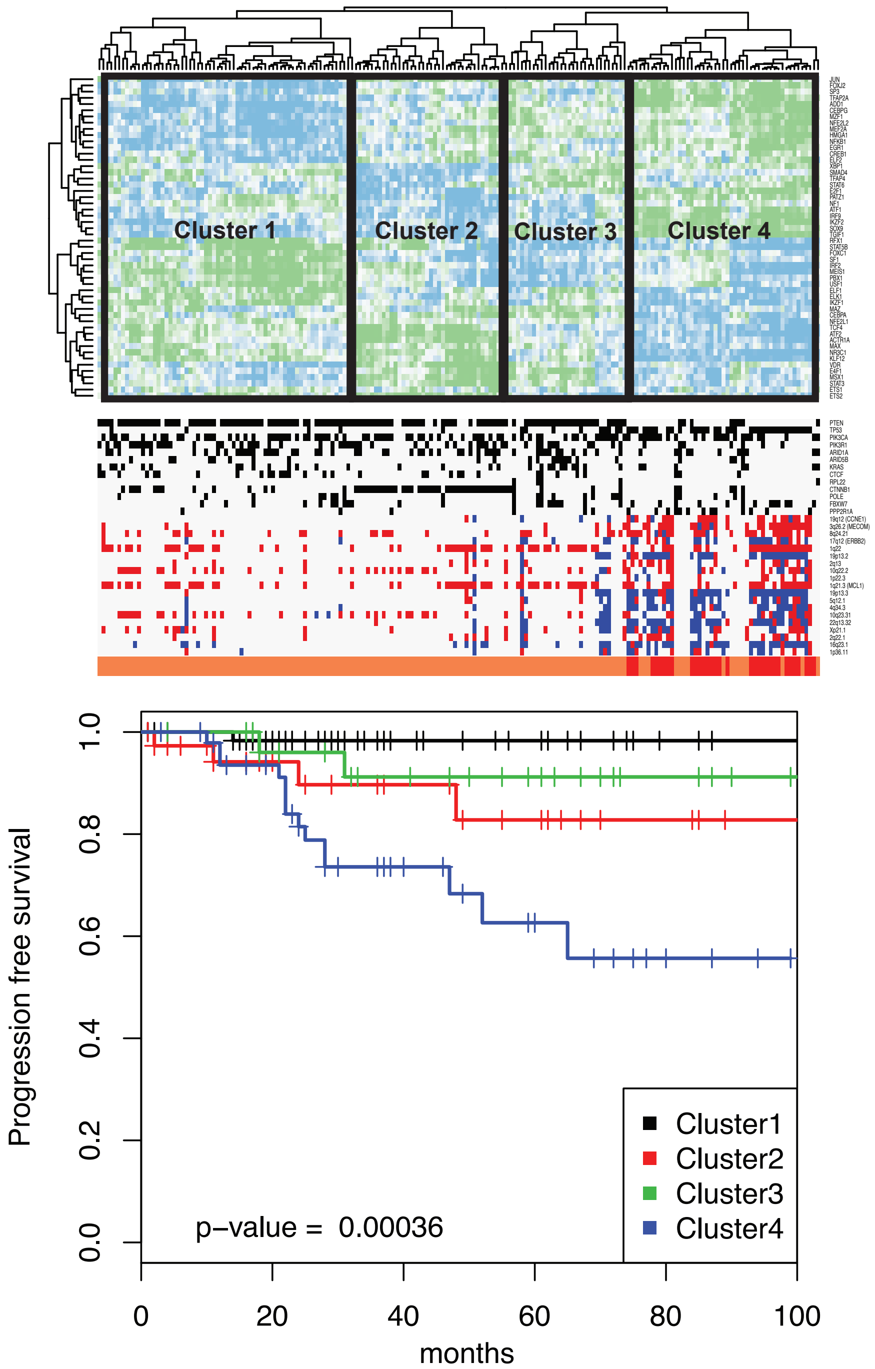

Supplementary Figure 24. We trained an affinity regression model on 183 tumors from the TCGA endometrial carcinoma (UCEC) study. The top heat map shows a clustering of tumors by inferred TF activities. The middle panel shows genomic aberration profiles of each tumor as well as histological subtypes derived from the corresponding TCGA UCEC study. Patterns of TF activities across tumors often correlated with patterns of (phospho)protein activities. Kaplan–Meier plot showing progression-free survival curves of UCEC patients (bottom panel) in the 4 inferred TF activity clusters in UCEC study (top panel).

Supplementary Table 1. Performance of the trained affinity regression models compared to nearest neighbor methods and models with randomized features

	Affinity Regression	Nearest Neighbor	Randomized motif-hit (D)	Randomized RPPA (P)	Randomized RPPA (P) and motif hit D
	**Mean ρ (± s.d.)**	**Mean ρ (± s.d.)**	**Mean ρ (± s.d.)**	**Mean ρ (± s.d.)**	**Mean ρ (± s.d.)**
BLCA	0.32 (± 0.05)	0.20 (± 0.06)	0.33 (± 0.05)	-0.05 (± 0.08)	-0.04 (± 0.08)
BRCA	0.24 (± 0.02)	0.21 (± 0.05)	0.21 (± 0.02)	0.03 (± 0.01)	0.02 (± 0.01)
COADREAD	0.22 (± 0.03)	0.07 (± 0.05)	0.21 (± 0.03)	0.00 (± 0.07)	0.00 (± 0.07)
GBM	0.34 (± 0.07)	0.18 (± 0.07)	0.33 (± 0.07)	0.01 (± 0.10)	0.01 (± 0.10)
HNSC	0.21 (± 0.05)	0.15 (± 0.05)	0.20 (± 0.04)	0.00 (± 0.03)	0.00 (± 0.03)
KIRC	0.26 (± 0.03)	0.22 (± 0.05)	0.04 (± 0.02)	0.00 (± 0.03)	0.00 (± 0.02)
LUAD	0.26 (± 0.03)	0.18 (± 0.05)	0.25 (± 0.02)	0.01 (± 0.05)	0.01 (± 0.04)
LUSC	0.28 (± 0.07)	0.08 (± 0.05)	0.27 (± 0.08)	-0.03 (± 0.1)	-0.03 (± 0.09)
OV	0.32 (± 0.03)	0.15 (± 0.02)	0.30 (± 0.02)	-0.03 (± 0.04)	-0.03 (± 0.03)
PRAD	0.27 (± 0.04)	0.15 (± 0.09)	0.25 (± 0.04)	0.01 (± 0.06)	0.01 (± 0.06)
UCEC	0.34 (± 0.03)	0.19 (± 0.03)	0.33 (± 0.03)	0.03 (± 0.05)	0.02 (± 0.04)
UCS	0.29 (± 0.07)	0.17 (± 0.07)	0.28 (± 0.06)	0.02 (± 0.08)	0.02(± 0.07)

Mean (± s.d.) ten-fold cross-validation Spearman correlations between predicted and actual gene expression changes relative to a median reference profile using the affinity regression model; nearest neighbor by (phospho)protein expression profile; and affinity regression models with randomized motif hits for each gene, randomized RPPA profiles for each tumor, and both.

Supplementary Table 2. Survival analysis for BLCA

Covariate	Inferred TFActivity				Gene expression profiles			
	P	Adj-*P*	Coef(bi)	HR[exp(bi)]	*P*	Adj-*P*	Coef(bi)	HR[exp(bi)]
IRF1	0.00	0.04	-0.83	0.11	0.16	0.32	-0.52	0.03
GATA2	0.01	0.04	0.79	-0.11	0.08	0.31	-0.44	0.05
MAX	0.01	0.04	-0.78	0.06	0.15	0.32	1.42	0.02
JUN	0.01	0.04	0.63	0.18	0.09	0.31	0.60	-0.15
NFKB1	0.00	0.04	-1.05	0.15	0.32	0.32	-0.10	0.00
TGIF1	0.00	0.04	-0.99	0.05	0.06	0.31	-1.06	0.00
TTF1	0.01	0.04	0.73	-0.09	0.32	0.32	0.07	0.00
FOXO1	0.01	0.04	-0.57	-0.04	0.07	0.31	-0.97	0.20
ATF6	0.01	0.04	1.03	-0.06	0.32	0.32	-0.12	0.00
CEBPG	0.01	0.04	-0.53	0.04	0.10	0.31	-1.15	0.03
FOXM1	0.01	0.04	1.07	-0.21	0.30	0.32	0.13	0.01
GTF2A1	0.01	0.04	0.48	0.04	0.12	0.31	0.74	0.02
ADD1	0.01	0.04	0.76	-0.11	0.30	0.32	-0.40	0.03
TCF3	0.02	0.05	0.71	0.05	0.12	0.31	-1.11	-0.01
YY1	0.02	0.05	-0.66	-0.01	0.31	0.32	0.28	0.00
FOXQ1	0.02	0.05	0.59	0.09	0.03	0.31	-0.27	-0.02
RORA	0.02	0.05	-0.70	0.03	0.08	0.31	0.54	-0.10
ARNT	0.02	0.05	-1.05	-0.01	0.31	0.32	0.20	0.01
NFE2L1	0.02	0.05	0.64	-0.11	0.11	0.31	1.46	-0.03

Cox proportional hazards regression models for TF regulators based on inferred TF activity (left) and TF mRNA values (right), considering TFs that attained significance by our empirical *P* value procedure in at least 5% of samples. We added clinical stage as a background factor for BLCA.

Supplementary Table 3. Survival analysis for KIRC

Covariate	Inferred TF Activity				Gene expression profiles			
	P	Adj-*P*	Coef (bi)	HR [exp(bi)]	*P*	Adj-*P*	Coef (bi)	HR [exp(bi)]
ETS1	2.E-10	7.E-09	1.22	-0.14	3.E-01	6.E-01	-0.63	-0.15
HMGA1	1.E-10	7.E-09	1.31	-0.23	1.E-03	1.E-02	1.17	-0.18
EBF1	7.E-10	2.E-08	-1.77	-0.18	9.E-01	9.E-01	-0.02	-0.12
SPI1	1.E-09	3.E-08	-1.34	-0.30	2.E-01	5.E-01	0.52	-0.08
BPTF	1.E-07	2.E-06	-1.31	-0.30	6.E-01	9.E-01	-0.58	-0.12
HNF1A	2.E-07	2.E-06	-1.07	-0.22	5.E-02	2.E-01	-0.60	-0.14
ESR1	3.E-07	2.E-06	-0.99	-0.26	9.E-01	9.E-01	-0.15	-0.11
NFE2L2	3.E-07	2.E-06	0.70	-0.23	7.E-01	9.E-01	-0.69	-0.14
POSTN	5.E-07	4.E-06	0.81	-0.26	9.E-01	9.E-01	0.06	-0.11
IRF2	7.E-07	5.E-06	-0.92	-0.27	6.E-01	9.E-01	1.14	-0.12
MAX	9.E-07	6.E-06	0.89	-0.18	7.E-01	9.E-01	1.03	-0.15
NFATC4	2.E-06	9.E-06	-1.00	-0.22	4.E-03	3.E-02	1.13	-0.10
VDR	2.E-06	1.E-05	1.25	-0.11	3.E-01	6.E-01	-0.49	-0.14
TCF12	2.E-06	1.E-05	0.93	-0.26	9.E-01	9.E-01	0.47	-0.12
SP3	2.E-06	1.E-05	0.94	-0.23	9.E-01	9.E-01	-0.48	-0.12
NFKB1	4.E-06	2.E-05	1.25	-0.19	4.E-01	7.E-01	-0.77	-0.15
RREB1	6.E-06	3.E-05	-0.76	-0.21	9.E-01	9.E-01	-0.10	-0.12
GTF2I	1.E-05	4.E-05	-1.02	-0.20	3.E-01	6.E-01	-0.64	-0.14
IKZF2	2.E-05	9.E-05	-0.80	-0.22	9.E-01	9.E-01	0.08	-0.12
CEBPB	2.E-05	9.E-05	0.94	-0.18	4.E-05	8.E-04	1.29	-0.25
USF1	5.E-05	2.E-04	-1.28	-0.15	5.E-05	8.E-04	2.78	-0.08
MZF1	6.E-05	2.E-04	0.69	-0.20	2.E-06	8.E-05	1.94	-0.16
EP300	7.E-05	2.E-04	-0.98	-0.23	4.E-01	7.E-01	-0.77	-0.13
FOXQ1	1.E-04	3.E-04	1.06	-0.28	9.E-01	9.E-01	0.06	-0.11
SMAD4	2.E-03	6.E-03	-0.82	-0.15	9.E-02	3.E-01	-1.92	-0.15
NR1H4	2.E-03	7.E-03	0.89	-0.18	9.E-01	9.E-01	-0.07	-0.12
STAT5B	4.E-03	1.E-02	0.59	-0.26	1.E-01	3.E-01	-1.68	-0.12
LEF1	4.E-03	1.E-02	0.94	-0.09	1.E-02	6.E-02	0.68	-0.24
MYC	5.E-03	1.E-02	-0.73	-0.18	2.E-01	5.E-01	0.58	-0.05
NFYB	6.E-03	2.E-02	-0.47	-0.17	3.E-03	2.E-02	3.14	-0.17
POU2F1	2.E-02	5.E-02	0.77	-0.19	4.E-02	1.E-01	1.42	-0.17
NFYA	2.E-02	5.E-02	0.55	-0.16	1.E-01	3.E-01	2.04	-0.09

Cox proportional hazards regression models for TF regulators based on inferred TF activity (left) and TF mRNA values (right), considering TFs that attained significance by our empirical *P* value procedure in at least 5% of samples. We added clinical stage as a background factor for KIRC.

Supplementary Table 4. Survival analysis for UCEC

Covariate	Inferred Protein Activity				Gene expression profiles			
	P	Adj-*P*	Coef (bi)	HR [exp(bi)]	*P*	Adj-*P*	Coef (bi)	HR [exp(bi)]
NFKB1	5.E-06	3.E-04	2.48	-10.28	5.E-02	5.E-02	-0.05	-14.06
FOXO1	9.E-06	3.E-04	2.04	-12.45	3.E-02	5.E-02	0.83	-13.97
SOX9	2.E-05	6.E-04	1.88	-11.82	1.E-02	4.E-02	0.99	-12.63
IKZF1	5.E-05	6.E-04	-1.63	-11.27	4.E-02	5.E-02	-0.33	-14.08
IKZF2	4.E-05	6.E-04	1.74	-12.16	4.E-02	5.E-02	0.34	-14.11
ELF1	4.E-05	6.E-04	-1.42	-13.43	4.E-02	5.E-02	0.60	-14.19
TFAP2A	7.E-05	6.E-04	1.36	-12.16	5.E-02	5.E-02	0.00	-14.06
FOXO4	7.E-05	6.E-04	-2.21	-11.42	5.E-02	5.E-02	-0.19	-14.05
TGIF1	9.E-05	7.E-04	1.62	-12.53	5.E-03	3.E-02	-2.53	-13.53
ATF6	1.E-04	9.E-04	-1.96	-12.67	2.E-03	3.E-02	2.91	-12.93
EGR1	2.E-04	1.E-03	1.48	-12.28	4.E-02	5.E-02	0.32	-14.15
FOXJ2	2.E-04	1.E-03	1.36	-12.56	4.E-02	5.E-02	-0.79	-14.08
KLF12	3.E-04	2.E-03	-1.48	-12.48	3.E-03	3.E-02	1.64	-12.86
IRF9	3.E-04	2.E-03	1.22	-12.56	5.E-02	5.E-02	0.15	-14.00
GATA2	6.E-04	3.E-03	-1.60	-12.64	3.E-02	5.E-02	0.46	-14.02
CEBPG	7.E-04	3.E-03	1.22	-12.65	2.E-02	5.E-02	1.24	-13.02
ELK1	9.E-04	3.E-03	-1.58	-11.56	4.E-03	3.E-02	3.23	-12.63
STAT5B	9.E-04	3.E-03	-0.86	-12.08	4.E-02	5.E-02	-1.14	-13.07
TCF4	9.E-04	3.E-03	-1.19	-12.37	4.E-02	5.E-02	-0.28	-13.99
ADD1	8.E-04	3.E-03	1.08	-12.08	4.E-02	5.E-02	0.60	-14.04
NR3C1	1.E-03	4.E-03	-1.17	-12.56	3.E-03	3.E-02	1.53	-13.07
ESRRA	1.E-03	4.E-03	-1.36	-12.90	4.E-02	5.E-02	-0.90	-14.04
ATF2	1.E-03	4.E-03	-1.07	-12.76	8.E-03	4.E-02	2.30	-14.23
MYB	2.E-03	4.E-03	-1.63	-11.98	2.E-02	5.E-02	-0.69	-12.64
TCF12	2.E-03	4.E-03	-1.98	-12.48	4.E-02	5.E-02	0.71	-14.12
NFE2L1	3.E-03	7.E-03	-1.21	-12.45	9.E-03	4.E-02	-3.29	-12.44
SMAD4	3.E-03	9.E-03	-1.28	-12.60	2.E-02	5.E-02	1.83	-13.25
MEF2A	4.E-03	9.E-03	1.20	-12.02	2.E-02	5.E-02	2.10	-14.32
JUN	4.E-03	9.E-03	0.92	-12.25	5.E-02	5.E-02	0.15	-14.09
SP3	4.E-03	9.E-03	0.98	-12.96	5.E-03	3.E-02	2.41	-13.16
NFE2L2	4.E-03	9.E-03	0.99	-12.38	4.E-02	5.E-02	0.58	-14.11
ATF1	4.E-03	1.E-02	0.99	-13.18	4.E-02	5.E-02	0.73	-14.17
HMGA1	5.E-03	1.E-02	1.05	-11.87	2.E-02	5.E-02	0.80	-12.75
IRF2	5.E-03	1.E-02	-1.02	-12.08	1.E-02	4.E-02	-2.93	-12.97
PBX1	5.E-03	1.E-02	-1.03	-12.40	2.E-03	3.E-02	1.90	-13.35
CEBPB	6.E-03	1.E-02	-1.27	-12.67	7.E-03	4.E-02	1.52	-12.77
ACTR1A	6.E-03	1.E-02	-0.95	-12.92	4.E-02	5.E-02	1.08	-13.88

MEIS1	6.E-03	1.E-02	-0.73	-12.18	3.E-02	5.E-02	-0.31	-13.08
MZF1	7.E-03	1.E-02	1.10	-12.15	2.E-02	5.E-02	-1.15	-12.87
SREBF1	7.E-03	1.E-02	1.11	-12.51	1.E-02	4.E-02	-1.56	-14.04
E2F1	7.E-03	1.E-02	0.79	-12.85	3.E-03	3.E-02	1.38	-12.32
RFX1	8.E-03	1.E-02	-0.69	-12.23	4.E-02	5.E-02	0.68	-13.99
PATZ1	9.E-03	1.E-02	0.77	-12.91	2.E-02	5.E-02	1.80	-13.02
STAT1	1.E-02	2.E-02	-0.99	-12.88	2.E-02	5.E-02	0.91	-12.80
ATF3	9.E-03	2.E-02	-1.13	-12.88	5.E-02	5.E-02	0.10	-14.08
MAX	1.E-02	2.E-02	-1.08	-14.12	4.E-02	5.E-02	-1.35	-14.13
MAZ	1.E-02	2.E-02	-0.81	-13.15	5.E-02	5.E-02	0.19	-14.03
NF1	2.E-02	2.E-02	0.86	-13.24	1.E-02	4.E-02	2.53	-13.21
CEBPA	2.E-02	3.E-02	-0.56	-12.99	3.E-02	5.E-02	0.54	-13.87
STAT3	2.E-02	3.E-02	-0.75	-13.44	5.E-02	5.E-02	-0.03	-14.06
stage	2.E-02	3.E-02	-14.05	1.23	2.E-02	5.E-02	-14.05	1.23
CREB1	2.E-02	3.E-02	0.68	-12.96	3.E-02	5.E-02	1.13	-14.16
GABPA	2.E-02	3.E-02	0.78	-13.97	4.E-02	5.E-02	0.65	-14.17
E4F1	3.E-02	3.E-02	-0.50	-14.23	2.E-02	5.E-02	-1.46	-13.06
STAT5A	3.E-02	3.E-02	0.61	-14.16	5.E-02	5.E-02	-0.02	-14.06
STAT6	3.E-02	4.E-02	0.36	-13.84	3.E-04	2.E-02	-1.86	-13.59
XBP1	3.E-02	4.E-02	0.52	-14.11	9.E-03	4.E-02	-1.33	-12.62
LEF1	3.E-02	4.E-02	-0.55	-13.81	2.E-03	3.E-02	-0.84	-13.54
ETS1	3.E-02	4.E-02	-0.43	-14.01	4.E-02	5.E-02	-0.28	-14.03
ARNT	3.E-02	4.E-02	0.47	-13.89	4.E-02	5.E-02	0.44	-14.02
SF1	3.E-02	4.E-02	-0.40	-14.01	4.E-02	5.E-02	1.57	-14.13
TFAP4	3.E-02	4.E-02	-0.44	-14.13	5.E-02	5.E-02	-0.04	-14.06
MYCN	4.E-02	4.E-02	0.38	-14.16	4.E-02	5.E-02	-0.17	-14.15
USF1	4.E-02	4.E-02	-0.25	-13.86	4.E-02	5.E-02	-0.96	-14.29
VDR	4.E-02	4.E-02	0.24	-13.92	4.E-02	5.E-02	0.41	-13.77
MSX1	4.E-02	4.E-02	-0.23	-14.13	3.E-02	5.E-02	-0.21	-13.97
TTF1	4.E-02	4.E-02	-0.27	-14.03	9.E-03	4.E-02	2.92	-12.95
JUND	4.E-02	4.E-02	-0.31	-13.95	4.E-02	5.E-02	0.55	-13.93
ETS2	4.E-02	4.E-02	0.21	-14.08	6.E-03	4.E-02	-1.82	-13.80
EP300	4.E-02	4.E-02	-0.26	-14.02	1.E-02	5.E-02	2.14	-13.26
FOXC1	4.E-02	4.E-02	-0.13	-14.01	3.E-02	5.E-02	-0.36	-14.03
ELF2	5.E-02	5.E-02	-0.06	-14.07	2.E-02	5.E-02	-2.79	-12.95

Cox proportional hazards regression models for TF regulators based on inferred TF activity (left) and TF mRNA values (right), considering TFs that attained significance by our empirical *P* value procedure in at least 5% of samples. We added histological subtype as a background factor for UCEC.

Supplementary Table 5. Univariate survival analysis for endometrioid carcinoma

Covariate	P-value (training-TCGA)	Coef(bi)	HR[exp(bi)]	P-value (validation)
TCF4	**0.0046**	-1.9973	0.1357	**0.0197**
IKZF2	**0.0151**	1.9923	7.3323	**0.0009**
IKZF1	0.0153	-1.6048	0.2009	0.8827
CEBPG	**0.0157**	1.5440	4.6833	**0.0051**
JUN	0.0184	1.0678	2.9090	0.5103
ELK1	0.0220	-1.5636	0.2094	0.3171
STAT5B	**0.0313**	-0.8514	0.4268	**0.0002**
HMGA1	**0.0353**	1.3982	4.0478	**0.0053**
MEIS1	**0.0399**	-0.9639	0.3814	**0.0003**

Supplementary Table 6. Transcription factors that show significant association with HPV status

TF	TCGA - TF activity		TCGA – TF mRNA		Sewell et al - TF activity		Pathway
	**P-value TF activity**	**Adj P-value TF activity**	**P-value TF mRNA**	**Adj P-value TF mRNA**	**P-value TF activity**	**Adj P-value TF activity**	
TGIF1	1.2E-06	**1.4E-04**	5.1E-01	7.6E-01	4.7E-04	**3.6E-03**	TGF-beta signaling
NFE2L2	5.0E-06	**3.0E-04**	6.5E-01	8.3E-01	2.1E-05	**6.1E-04**	Oxidative stress
ATF2	1.0E-05	**4.2E-04**	5.9E-01	8.1E-01	1.6E-03	**4.9E-03**	TGF-beta signaling
TFAP4	3.3E-05	**6.8E-04**	4.2E-02	1.6E-01	9.5E-04	**4.2E-03**	cell cycle
ADD1	2.3E-05	**6.8E-04**	3.2E-01	5.6E-01	8.1E-01	8.7E-01	
RORA	3.1E-05	**6.8E-04**	5.1E-01	7.6E-01	1.1E-04	**1.4E-03**	WNT signaling
KLF12	7.8E-05	**1.3E-03**	1.6E-02	8.4E-02	7.3E-05	**1.3E-03**	cell proliferation
GTF2I	8.7E-05	**1.3E-03**	9.6E-01	9.9E-01	1.3E-03	**4.4E-03**	cell cycle
MEIS1	1.5E-04	**1.5E-03**	3.9E-04	1.2E-02	1.5E-02	3.1E-02	cell cycle
SREBF1	1.4E-04	**1.5E-03**	1.6E-02	8.4E-02	1.3E-05	**6.1E-04**	metabolism
ATF4	1.6E-04	**1.5E-03**	4.7E-02	1.7E-01	6.0E-04	**4.0E-03**	MAPK
FOXC1	1.5E-04	**1.5E-03**	1.4E-01	3.1E-01	2.0E-03	**5.5E-03**	TGF-beta signaling
TBP	1.1E-04	**1.5E-03**	3.1E-01	5.5E-01	1.5E-04	**1.5E-03**	
JUND	2.3E-04	**2.0E-03**	2.7E-01	4.8E-01	6.7E-04	**4.2E-03**	TGF-beta signaling
LEF1	2.9E-04	**2.2E-03**	6.5E-02	2.0E-01	1.1E-03	**4.2E-03**	TGF-beta signaling
GABPA	2.9E-04	**2.2E-03**	2.1E-01	4.1E-01	2.8E-04	**2.5E-03**	
RARA	3.4E-04	**2.3E-03**	1.5E-02	8.4E-02	2.8E-04	**2.5E-03**	
NFATC4	3.2E-04	**2.3E-03**	1.5E-01	3.2E-01	2.6E-01	3.4E-01	WNT signaling
FOXO3	3.7E-04	**2.4E-03**	5.9E-01	8.1E-01	8.4E-05	**1.3E-03**	TGF-beta signaling
JUN	4.8E-04	**2.9E-03**	8.1E-01	9.0E-01	1.1E-05	**6.1E-04**	TGF-beta signaling
AHR	5.1E-04	**3.0E-03**	2.5E-03	2.7E-02	2.5E-05	**6.1E-04**	
IKZF1	5.7E-04	**3.2E-03**	1.9E-01	3.8E-01	8.5E-03	1.7E-02	
E2F1	6.5E-04	**3.3E-03**	2.7E-03	2.7E-02	4.6E-02	7.7E-02	cell cycle
SPI1	6.2E-04	**3.3E-03**	7.4E-01	8.8E-01	2.1E-01	2.9E-01	
SOX9	9.6E-04	**4.7E-03**	1.7E-01	3.5E-01	7.5E-04	**4.2E-03**	
ELF2	1.5E-03	**7.0E-03**	1.4E-05	**1.7E-03**	1.8E-03	**5.2E-03**	IL2 signaling
ELF1	1.8E-03	**7.9E-03**	4.3E-01	6.8E-01	2.5E-03	**6.5E-03**	IL2 signaling
STAT1	2.0E-03	**8.3E-03**	4.8E-01	7.5E-01	1.8E-03	**5.2E-03**	TGF-beta signaling
NF1	1.9E-03	**8.3E-03**	7.4E-01	8.8E-01	8.2E-02	1.3E-01	WNT signaling
SMAD4	2.3E-03	**9.4E-03**	1.2E-04	**7.2E-03**	1.1E-03	**4.2E-03**	TGF-beta signaling

Supplementary Table 7. Transcription factors that show significant association with uterine corpus endometrial carcinoma histological subtype

TF	TCGA - TF activity		TCGA – TF mRNA	
	P-value TF activity	Adj P-value TF activity	P-value TF mRNA	Adj P-value TF mRNA
IKZF2	4.64E-18	**5.57E-16**	9.28E-01	9.44E-01
IRF9	5.09E-17	**2.37E-15**	1.47E-03	5.19E-03
NFE2L1	6.54E-17	**2.37E-15**	1.91E-01	2.75E-01
TGIF1	7.89E-17	**2.37E-15**	2.03E-01	2.86E-01
ATF1	1.56E-16	**3.75E-15**	2.76E-02	6.49E-02
TFAP2A	2.81E-16	**5.61E-15**	2.60E-01	3.51E-01
KLF12	4.72E-16	**8.09E-15**	5.17E-03	1.55E-02
ADD1	2.33E-15	**3.49E-14**	7.16E-02	1.34E-01
NFKB1	3.42E-15	**4.23E-14**	1.51E-01	2.27E-01
FOXJ2	3.52E-15	**4.23E-14**	2.53E-01	3.48E-01
ELF1	4.20E-15	**4.59E-14**	1.04E-01	1.71E-01
ESRRA	6.53E-15	**6.53E-14**	4.95E-01	5.94E-01
FOXO1	9.54E-15	**8.81E-14**	5.60E-01	6.59E-01
FOXO4	1.20E-14	**1.03E-13**	8.83E-03	2.46E-02
NR3C1	1.61E-14	**1.29E-13**	1.40E-03	5.19E-03
ATF2	2.27E-14	**1.70E-13**	3.49E-01	4.36E-01
SOX9	5.34E-14	**3.56E-13**	1.11E-01	1.75E-01
IKZF1	5.19E-14	**3.56E-13**	1.43E-01	2.17E-01
ATF6	6.15E-14	**3.88E-13**	3.56E-02	7.63E-02
NFYB	6.50E-14	**3.90E-13**	9.51E-01	9.51E-01
CEBPB	7.92E-14	**4.52E-13**	9.93E-08	**1.32E-06**
CEBPA	1.55E-13	**8.44E-13**	4.07E-04	1.75E-03
NR1H3	4.91E-13	**2.56E-12**	6.32E-01	7.30E-01
ACTR1A	6.10E-13	**3.05E-12**	8.67E-02	1.55E-01
SP3	4.04E-12	**1.94E-11**	2.29E-02	5.73E-02
TCF3	7.16E-12	**3.30E-11**	3.53E-02	7.63E-02
EGR1	7.94E-12	**3.53E-11**	6.48E-01	7.30E-01
AHR	1.51E-11	**6.46E-11**	1.89E-03	6.31E-03
CEBPG	1.76E-11	**7.26E-11**	1.03E-04	**5.39E-04**
NFE2L2	1.94E-11	**7.77E-11**	9.08E-02	1.57E-01
E2F1	3.06E-11	**1.18E-10**	1.64E-09	**3.93E-08**
MAX	4.56E-11	**1.71E-10**	7.16E-02	1.34E-01
MEF2D	5.43E-11	**1.97E-10**	3.56E-02	7.63E-02
MTF1	5.99E-11	**2.11E-10**	3.12E-01	4.07E-01
STAT3	6.95E-11	**2.32E-10**	1.01E-01	1.69E-01
RELA	6.78E-11	**2.32E-10**	3.36E-01	4.24E-01
MYB	2.40E-10	**7.78E-10**	4.83E-08	**7.25E-07**

PAX8	4.46E-10	**1.41E-09**	1.82E-07	**1.82E-06**
MYC	6.96E-10	**2.14E-09**	1.16E-06	**9.95E-06**
E4F1	7.29E-10	**2.19E-09**	8.90E-01	9.19E-01
STAT5B	1.03E-09	**3.03E-09**	5.72E-02	1.14E-01
CREBBP	2.41E-09	**6.89E-09**	9.66E-02	1.63E-01
PATZ1	3.78E-09	**1.06E-08**	3.53E-02	7.63E-02
TCF4	4.04E-09	**1.10E-08**	1.63E-03	5.58E-03
ESR1	6.03E-09	**1.57E-08**	5.65E-14	**3.39E-12**
GATA2	6.03E-09	**1.57E-08**	9.16E-02	1.57E-01
TBP	8.39E-09	**2.14E-08**	7.65E-04	3.16E-03
POSTN	1.44E-08	**3.61E-08**	3.28E-01	4.19E-01
ELK1	2.17E-08	**5.32E-08**	1.60E-02	4.18E-02
RORA	2.69E-08	**6.45E-08**	1.75E-06	**1.40E-05**
XBP1	3.76E-08	**8.85E-08**	2.70E-06	**2.03E-05**
STAT1	7.62E-08	**1.76E-07**	1.14E-08	**2.28E-07**
HMGA1	1.58E-07	**3.51E-07**	1.39E-13	**5.54E-12**
STAT5A	1.58E-07	**3.51E-07**	6.75E-01	7.43E-01
TCF12	2.31E-07	**5.03E-07**	3.83E-03	1.18E-02
IRF2	2.87E-07	**6.14E-07**	1.29E-03	5.01E-03
PPARA	1.37E-06	**2.89E-06**	6.51E-01	7.30E-01
LEF1	1.40E-06	**2.89E-06**	2.36E-08	**4.05E-07**
TP53	1.81E-06	**3.68E-06**	1.52E-02	4.07E-02
MAZ	1.91E-06	**3.83E-06**	1.93E-01	2.75E-01
IRF1	2.80E-06	**5.51E-06**	2.75E-01	3.66E-01
ETS2	3.12E-06	**6.04E-06**	3.49E-04	1.55E-03
SPI1	3.48E-06	**6.62E-06**	1.26E-01	1.96E-01
MZF1	5.91E-06	**1.11E-05**	6.56E-03	1.87E-02
MSX1	1.03E-05	**1.90E-05**	1.43E-07	**1.59E-06**
HSF1	1.54E-05	**2.80E-05**	2.62E-04	1.21E-03
TTF1	1.92E-05	**3.43E-05**	1.07E-01	1.72E-01
HIF1A	3.34E-05	**5.90E-05**	1.07E-01	1.72E-01
ATF4	6.21E-05	**1.08E-04**	1.65E-01	2.42E-01
MECOM	8.47E-05	**1.45E-04**	9.43E-01	9.50E-01
MEF2A	1.42E-04	**2.40E-04**	6.51E-01	7.30E-01
TFCP2	1.99E-04	**3.32E-04**	8.29E-01	8.79E-01
FOXO3	2.14E-04	**3.47E-04**	5.48E-02	1.12E-01
MEIS1	2.14E-04	**3.47E-04**	6.48E-01	7.30E-01
SMAD3	3.44E-04	**5.50E-04**	6.12E-06	**4.32E-05**
ELF2	4.37E-04	**6.90E-04**	3.03E-05	**1.82E-04**
RFX1	4.68E-04	**7.30E-04**	9.84E-04	3.94E-03
RREB1	5.45E-04	**8.38E-04**	3.66E-02	7.71E-02

GABPA	6.33E-04	**9.62E-04**	7.46E-01	8.14E-01

Supplementary Table 8. **(Phospho)proteins that show significant association with uterine corpus endometrial carcinoma histological subtype**

(phospho)protein	TCGA - Inferred (phospho)protein activity		TCGA – (phospho)protein expression	
	P-value (phospho)protein activity	Adjusted P-value (phospho)protein activity	P-value (phospho)protein expression	Adjusted P-value (phospho)protein expression
eEF2	2.41E-16	**2.97E-14**	1.40E-06	**2.18E-05**
VHL	3.17E-16	**2.97E-14**	3.11E-04	2.08E-03
p38_MAPK	1.10E-15	**5.73E-14**	1.99E-04	1.49E-03
p70S6K_pT389	1.49E-15	**5.73E-14**	7.25E-04	3.66E-03
HER2	1.53E-15	**5.73E-14**	2.29E-02	5.85E-02
p53	3.13E-15	**9.75E-14**	6.98E-12	**6.52E-10**
Chk2_pT68	1.88E-13	**4.52E-12**	2.47E-09	**7.69E-08**
PCNA	1.93E-13	**4.52E-12**	4.61E-01	5.77E-01
YB-1	5.78E-13	**1.11E-11**	2.37E-05	**2.77E-04**
p27_pT198	5.94E-13	**1.11E-11**	3.63E-02	8.27E-02
Cyclin_B1	1.74E-12	**2.95E-11**	5.87E-07	**1.12E-05**
PKC-pan_BetaII_pS660	4.60E-12	**7.18E-11**	4.90E-02	1.01E-01
Akt	7.54E-12	**1.08E-10**	4.15E-03	1.58E-02
Bad_pS112	1.33E-11	**1.77E-10**	8.45E-03	2.93E-02
Notch1	5.04E-11	**6.28E-10**	9.52E-02	1.76E-01
PREX1	9.80E-11	**1.13E-09**	2.37E-04	1.64E-03
4E-BP1	1.03E-10	**1.13E-09**	2.91E-01	4.05E-01
PTEN	1.80E-10	**1.87E-09**	1.18E-06	**2.01E-05**
Annexin-1	2.29E-10	**2.04E-09**	2.71E-05	**2.98E-04**
RBM15	2.08E-10	**2.04E-09**	2.95E-01	4.05E-01
G6PD	2.18E-10	**2.04E-09**	4.68E-01	5.78E-01
PR	4.56E-10	**3.88E-09**	1.74E-04	1.36E-03
EGFR	1.06E-09	**8.60E-09**	1.38E-01	2.31E-01
Rad51	1.24E-09	**9.69E-09**	1.71E-01	2.68E-01
Shc_pY317	1.60E-09	**1.20E-08**	9.43E-01	9.68E-01
GAPDH	1.92E-09	**1.38E-08**	1.38E-02	4.02E-02
PKC-delta_pS664	3.23E-09	**2.24E-08**	1.53E-03	6.80E-03
AR	3.70E-09	**2.47E-08**	4.52E-01	5.75E-01
PI3K-p110-alpha	6.31E-09	**4.07E-08**	4.29E-13	**8.01E-11**
mTOR	7.52E-09	**4.69E-08**	3.34E-04	2.11E-03

14-3-3_zeta	9.16E-09	**5.53E-08**	1.89E-01	2.85E-01
ER-alpha_pS118	9.57E-09	**5.59E-08**	9.21E-10	**3.44E-08**
Smad1	1.19E-08	**6.54E-08**	4.42E-02	9.38E-02
Rictor_pT1135	1.16E-08	**6.54E-08**	1.94E-01	2.90E-01
Syk	1.24E-08	**6.64E-08**	1.32E-04	1.07E-03
Akt_pS473	1.44E-08	**7.51E-08**	8.46E-11	**3.95E-09**
Annexin_VII	1.75E-08	**8.86E-08**	8.58E-01	9.09E-01
E-Cadherin	4.27E-08	**2.09E-07**	3.84E-07	**8.98E-06**
HER2_pY1248	4.36E-08	**2.09E-07**	1.22E-01	2.13E-01
Acetyl-a-Tubulin-Lys40	4.54E-08	**2.12E-07**	4.55E-04	2.47E-03
FoxM1	4.94E-08	**2.15E-07**	8.96E-06	**1.29E-04**
ERK2	4.94E-08	**2.15E-07**	2.14E-03	9.02E-03
XBP1	4.83E-08	**2.15E-07**	1.60E-02	4.48E-02
IGFBP2	8.79E-08	**3.74E-07**	7.22E-02	1.42E-01
Akt_pT308	1.52E-07	**6.31E-07**	5.56E-11	**3.47E-09**
ACC_pS79	1.93E-07	**7.85E-07**	4.56E-01	5.77E-01
14-3-3_beta	2.22E-07	**8.82E-07**	4.78E-02	1.00E-01
Chk2	2.70E-07	**1.05E-06**	3.18E-05	**3.31E-04**
Raptor	3.62E-07	**1.38E-06**	3.64E-04	2.20E-03
beta-Catenin	5.99E-07	**2.24E-06**	1.86E-02	4.91E-02
Src_pY416	8.76E-07	**3.21E-06**	1.74E-02	4.74E-02
VEGFR2	1.14E-06	**4.10E-06**	4.41E-01	5.65E-01
SF2	1.20E-06	**4.25E-06**	7.27E-02	1.42E-01
Bap1-c-4	1.40E-06	**4.84E-06**	4.63E-01	5.77E-01
HER3	2.85E-06	**9.70E-06**	9.52E-02	1.76E-01
Fibronectin	3.35E-06	**1.12E-05**	1.11E-01	1.97E-01
PDK1_pS241	3.67E-06	**1.20E-05**	1.29E-03	5.90E-03
ER-alpha	4.54E-06	**1.46E-05**	5.99E-07	**1.12E-05**
FOXO3a_pS318_S321	5.32E-06	**1.69E-05**	3.14E-01	4.25E-01
p27	5.41E-06	**1.69E-05**	5.17E-01	6.27E-01
FOXO3a	5.81E-06	**1.78E-05**	2.94E-01	4.05E-01
mTOR_pS2448	6.01E-06	**1.79E-05**	5.23E-03	1.96E-02
HER3_pY1289	6.01E-06	**1.79E-05**	2.93E-02	7.32E-02
Beclin	6.56E-06	**1.92E-05**	7.67E-01	8.34E-01
MAPK_pT202_Y204	1.03E-05	**2.96E-05**	1.41E-01	2.31E-01
N-Ras	1.30E-05	**3.69E-05**	9.78E-01	9.88E-01
P-Cadherin	2.05E-05	**5.71E-05**	6.35E-01	7.24E-01
Cyclin_D1	2.30E-05	**6.32E-05**	3.43E-02	8.03E-02
PEA-15	2.58E-05	**6.98E-05**	9.57E-01	9.78E-01
Heregulin	5.14E-05	**1.37E-04**	6.48E-01	7.30E-01
Cyclin_E1	5.83E-05	**1.54E-04**	5.25E-08	**1.40E-06**

Src_pY527	7.48E-05	**1.94E-04**	5.29E-01	6.34E-01
ATM	8.34E-05	**2.14E-04**	3.80E-01	4.97E-01
Claudin-7	8.47E-05	**2.14E-04**	3.02E-02	7.33E-02
Caveolin-1	9.72E-05	**2.42E-04**	6.35E-01	7.24E-01
eIF4E	1.11E-04	**2.74E-04**	7.49E-01	8.19E-01
Caspase-7_cleavedD198	1.13E-04	**2.75E-04**	2.76E-01	3.95E-01
NDRG1_pT346	1.85E-04	**4.44E-04**	1.61E-01	2.55E-01
c-Kit	2.37E-04	**5.61E-04**	6.12E-02	1.23E-01
CD31	2.66E-04	**6.22E-04**	5.43E-01	6.47E-01
HSP70	3.03E-04	**6.99E-04**	6.42E-04	3.33E-03

Supplementary Table 9. Association of the four inferred TF activity clusters with the three mRNA clusters reported from the UCEC TCGA network.

		Inferred TF activity Clusters			
		I	II	III	IV
TCGA mRNA Clusters	Mitotic	1(1)	0	10(4)	44(4)
	Hormonal	32(8)	18(17)	11(1)	0
	Immunoreactive	32(4)	20(19)	9	5(1)

The numbers in parentheses indicate the numbers of mutant *CTBBB1* cases in the subgroups of patients of different subtypes

Supplementary Table 10. Association of the four inferred TF activity clusters with the integrated clusters identified on the basis of mutation spectrum, copy-number variation and MSI status reported from the UCEC TCGA network.

		Inferred TF activity Clusters			
		I	II	III	IV
Integrated Clusters from TCGA	*POLE*	4(1)	1(0)	7(3)	3(3)
	MSI	19(0)	8(8)	15(2)	15(2)
	CN-low	39(12)	27(26)	3(0)	3(0)
	CN-high	2(0)	1(1)	5(0)	5(0)

The numbers in parentheses indicate the numbers of mutant CTBBB1 cases in the subgroups of patients of different subtypes

Supplementary Table 11. Transcription factors that show significant association with *CTNNB1* status in TCGA UCEC study

TF	TCGA - TF activity			TCGA - TF mRNA			MDACC - TF activity		
	P-value TF activity	Adj P-value TF activity	Effect size	P-value TF mRNA	Adj P-value TF mRNA	Effect size	P-value TF activity	Adj P-value TF activity	Effect size
ATF2	6.17E-19	**7.41E-17**	0.74	7.66E-01	8.43E-01	0.01	5.97E-06	**7.96E-05**	0.33
ACTR1A	1.87E-18	**1.12E-16**	0.66	1.93E-02	5.51E-02	0.04	1.45E-08	**1.74E-06**	0.31
NFYA	1.08E-17	**4.33E-16**	0.28	3.50E-01	5.18E-01	0.02	3.03E-04	**1.82E-03**	0.09
CEBPB	1.69E-17	**5.07E-16**	0.50	4.89E-07	**8.38E-06**	-0.20	2.92E-07	**7.00E-06**	0.28
ATF1	2.65E-17	**6.37E-16**	-0.59	3.73E-01	5.32E-01	-0.02	1.96E-05	**2.35E-04**	-0.24
PATZ1	6.71E-17	**1.34E-15**	-0.73	8.51E-01	9.04E-01	0.00	1.09E-07	**4.38E-06**	-0.36
IRF9	6.61E-15	**1.13E-13**	-0.53	3.82E-03	1.39E-02	-0.11	3.84E-05	**3.84E-04**	-0.26
STAT3	1.19E-14	**1.78E-13**	0.53	4.01E-01	5.52E-01	-0.02	2.09E-06	**4.19E-05**	0.26
MSX1	1.48E-14	**1.97E-13**	0.61	1.41E-03	**5.84E-03**	0.37	2.13E-03	**7.31E-03**	0.19
MAF	7.18E-14	**8.61E-13**	-0.40	1.37E-05	**1.10E-04**	0.24	1.83E-02	4.67E-02	-0.10
DBP	1.18E-13	**1.29E-12**	-0.33	1.72E-02	5.19E-02	-0.08	1.90E-02	4.67E-02	-0.09
MYC	1.50E-13	**1.50E-12**	-0.36	5.55E-01	6.67E-01	0.03	5.24E-04	**2.51E-03**	-0.14
TCF4	2.02E-13	**1.78E-12**	0.70	1.59E-06	**2.38E-05**	0.23	6.89E-05	**6.30E-04**	0.31
XBP1	2.08E-13	**1.78E-12**	-0.51	9.04E-01	9.29E-01	0.01	1.16E-04	**8.17E-04**	-0.22
TEAD1	2.84E-13	**2.27E-12**	-0.39	4.07E-01	5.55E-01	0.03	1.60E-03	**5.99E-03**	-0.14
VDR	4.28E-13	**3.21E-12**	0.65	1.33E-04	**8.40E-04**	0.17	2.38E-03	**7.68E-03**	0.19
SF1	3.59E-12	**2.53E-11**	-0.53	4.12E-01	5.55E-01	-0.01	3.04E-06	**5.21E-05**	-0.25
STAT5A	5.86E-12	**3.91E-11**	-0.44	4.81E-01	6.22E-01	-0.02	7.35E-05	**6.30E-04**	-0.19
MYCN	1.04E-11	**6.59E-11**	0.45	1.30E-01	2.68E-01	0.15	3.27E-05	**3.56E-04**	0.23
EP300	3.54E-11	**2.12E-10**	-0.37	4.94E-01	6.30E-01	-0.02	7.72E-04	**3.31E-03**	-0.13
TCF3	4.13E-11	**2.36E-10**	-0.34	1.51E-03	**6.02E-03**	0.08	8.70E-08	**4.38E-06**	-0.21
PPARA	6.76E-11	**3.69E-10**	-0.34	9.18E-01	9.34E-01	0.00	5.56E-06	**7.96E-05**	-0.21
SMAD4	1.10E-10	**5.76E-10**	-0.46	1.99E-01	3.63E-01	0.03	3.97E-04	**1.98E-03**	-0.21
E4F1	5.07E-10	**2.53E-09**	0.48	2.83E-01	4.60E-01	-0.04	5.29E-03	1.55E-02	0.15
CEBPA	6.90E-10	**3.31E-09**	0.48	5.13E-06	**5.59E-05**	-0.25	8.29E-03	2.37E-02	0.20
ETS2	2.02E-09	**9.33E-09**	0.48	1.27E-11	**7.61E-10**	0.26	6.81E-04	**3.03E-03**	0.21
NF1	2.19E-09	**9.72E-09**	-0.40	4.61E-03	1.54E-02	0.08	2.66E-04	**1.68E-03**	-0.19
NR3C1	2.77E-09	**1.14E-08**	0.48	1.40E-01	2.85E-01	-0.08	2.08E-07	**6.23E-06**	0.27
JUN	2.69E-09	**1.14E-08**	0.58	1.56E-01	3.08E-01	0.06	4.03E-02	9.13E-02	0.19
TGIF1	3.11E-09	**1.24E-08**	-0.41	4.71E-06	**5.59E-05**	0.15	1.04E-03	**4.29E-03**	-0.14
TP53	3.96E-09	**1.53E-08**	0.30	4.66E-04	**2.54E-03**	0.10	1.19E-03	**4.75E-03**	0.13
SP1	1.51E-08	**5.67E-08**	0.25	5.61E-01	6.67E-01	0.01	9.69E-05	**7.27E-04**	0.18
TFAP4	1.62E-07	**5.88E-07**	-0.35	2.53E-02	6.91E-02	-0.05	2.08E-02	4.99E-02	-0.15
IKZF2	2.17E-07	**7.65E-07**	-0.35	1.94E-01	3.58E-01	-0.05	8.25E-05	**6.60E-04**	-0.15
TFAP2A	5.46E-07	**1.87E-06**	-0.39	5.57E-01	6.67E-01	-0.06	1.24E-03	**4.81E-03**	-0.27
ESRRA	8.49E-07	**2.83E-06**	0.29	5.83E-02	1.49E-01	-0.05	2.12E-03	**7.31E-03**	0.11

FOXC1	2.60E-06	**8.42E-06**	-0.35	2.24E-01	3.95E-01	-0.12	2.12E-03	**7.31E-03**	-0.18
E2F1	3.21E-06	**1.01E-05**	-0.49	4.90E-04	**2.56E-03**	-0.19	4.08E-03	1.22E-02	-0.25
TEF	3.86E-06	**1.19E-05**	0.24	5.03E-03	1.63E-02	0.09	2.73E-01	4.00E-01	0.04
FOXQ1	4.04E-06	**1.21E-05**	-0.21	7.86E-06	**6.73E-05**	0.44	7.58E-01	8.33E-01	-0.02
JUND	4.39E-06	**1.29E-05**	-0.27	7.64E-03	2.41E-02	-0.10	4.93E-01	6.15E-01	-0.04
GATA2	6.12E-06	**1.75E-05**	0.24	8.82E-01	9.26E-01	0.01	3.36E-04	**1.83E-03**	0.11
MAX	8.30E-06	**2.32E-05**	0.29	3.35E-01	5.18E-01	-0.02	3.75E-04	**1.96E-03**	0.19
ELF2	9.50E-06	**2.59E-05**	0.31	3.00E-03	1.13E-02	0.04	2.97E-01	4.30E-01	0.06
PAX8	1.89E-05	**5.05E-05**	0.21	1.07E-01	2.33E-01	-0.12	2.39E-03	**7.68E-03**	0.13
FOXA2	2.17E-05	**5.66E-05**	0.23	2.59E-07	**5.17E-06**	0.60	4.20E-02	9.23E-02	0.11
SOX9	2.35E-05	**6.00E-05**	-0.29	8.29E-02	1.91E-01	0.15	4.38E-02	9.23E-02	-0.11
HSF1	9.89E-05	**2.47E-04**	0.21	5.70E-04	**2.85E-03**	-0.08	2.35E-01	3.72E-01	0.06
KLF12	1.21E-04	**2.96E-04**	0.30	6.00E-06	**6.00E-05**	0.24	5.49E-04	**2.54E-03**	0.22
NFE2L1	1.61E-04	**3.86E-04**	0.21	9.53E-01	9.53E-01	0.00	2.45E-04	**1.63E-03**	0.14
TTF1	2.52E-04	**5.92E-04**	0.23	2.20E-01	3.94E-01	0.03	1.13E-02	3.15E-02	0.13
MEF2A	5.09E-04	**1.15E-03**	0.24	7.34E-01	8.16E-01	0.01	2.43E-03	**7.68E-03**	0.18
HIF1A	5.08E-04	**1.15E-03**	-0.14	9.06E-01	9.29E-01	0.00	3.84E-01	5.18E-01	-0.03
NFATC4	5.38E-04	**1.20E-03**	-0.18	1.38E-03	**5.84E-03**	0.15	1.87E-01	3.21E-01	-0.07
ZEB1	6.79E-04	**1.48E-03**	-0.21	7.26E-06	**6.70E-05**	0.25	2.63E-01	4.00E-01	-0.06
RELA	8.56E-04	**1.83E-03**	-0.16	5.50E-02	1.44E-01	-0.03	1.90E-01	3.22E-01	-0.05
RUNX1	8.79E-04	**1.85E-03**	0.11	1.06E-04	**7.50E-04**	0.18	1.90E-02	4.67E-02	0.06
TFCP2	1.53E-03	**3.13E-03**	-0.17	7.19E-01	8.07E-01	0.01	1.48E-02	3.93E-02	-0.12
STAT6	1.54E-03	**3.13E-03**	-0.28	3.83E-01	5.34E-01	-0.03	3.63E-01	5.01E-01	-0.07
REST	2.00E-03	**3.99E-03**	0.12	2.28E-01	3.96E-01	-0.06	5.13E-01	6.22E-01	0.02
TCF12	2.15E-03	**4.10E-03**	0.18	9.02E-09	**3.61E-07**	0.18	3.40E-03	1.05E-02	0.14
GTF2I	2.15E-03	**4.10E-03**	-0.11	4.53E-03	1.54E-02	0.07	1.91E-02	4.67E-02	-0.08
FOXO1	2.09E-03	**4.10E-03**	-0.17	5.16E-01	6.39E-01	0.02	6.35E-02	1.27E-01	-0.06
USF1	3.63E-03	**6.80E-03**	-0.28	7.88E-02	1.85E-01	-0.04	2.33E-01	3.72E-01	-0.08
MEF2D	4.77E-03	**8.81E-03**	0.13	3.01E-01	4.82E-01	-0.03	3.23E-04	**1.83E-03**	0.14

Supplementary Table 12. Comparison of association analysis with SILAC-based phosphoproteomic analysis using isogenic knock-in breast cell lines harboring mutations of PIK3CA[2]

	TFs associated with *PIK3CA* mutation in BRCA	TFs not associated with *PIK3CA* mutation in BRCA
TF protein abundance changed between MCF10A and $MCF10A^{mutE545K}$/ $MCF10A^{mutH1047R}$	ADD1, FOXO3, HMGA1, HSF1, JUND, NF1, POU2F1, STAT3	SP1, MAX, JUN

Supplementary Table 13. Comparison of association analysis with protein microarray-based AKT1 kinase assay[2]

	TFs associated with *PIK3CA* mutation in BRCA	TFs not associated with *PIK3CA* mutation in BRCA
AKT substrate	ATF6, ETS1, SOX9, TEAD1	SMAD3, STAT6

Supplementary Table 14. **Summary of datasets used in the study**

**Dataset**	Summary
TF binding sites	Transcription factor binding site defined in the TRANSFAC for each gene from MSigDB
TCGA bladder urothelial carcinoma (BLCA)	115 tumor samples – RNA-seq, RPPA, MAF, copy profiles by SNP array, clinical data
TCGA breast cancer (BRCA)	368 tumor samples – RNA-seq, RPPA, MAF, copy profiles by SNP array, clinical data
TCGA colorectal adenocarcinoma (COADREAD)	150 tumor samples – RNA-seq, RPPA, MAF, copy profiles by SNP array, clinical data
TCGA glioblastoma multiforme (GBM)	58 tumor samples – RNA-seq, RPPA, MAF, copy profiles by SNP array, clinical data
TCGA head and neck squamous carcinoma (HNSC)	194 tumor samples – RNA-seq, RPPA, MAF, copy profiles by SNP array, clinical data
TCGA kidney renal cell clear carcinoma (KIRC)	376 tumor samples – RNA-seq, RPPA, MAF, copy profiles by SNP array, clinical data
TCGA lung adenocarcinoma (LUAD)	216 tumor samples – RNA-seq, RPPA, MAF, copy profiles by SNP array, clinical data
TCGA lung squamous cell carcinoma (LUSC)	106 tumor samples – RNA-seq, RPPA, MAF, copy profiles by SNP array, clinical data
TCGA ovarian carcinoma (OV)	164 tumor samples – RNA-seq, RPPA, MAF, copy profiles by SNP array, clinical data
TCGA prostate cancer (PRAD)	159 tumor samples – RNA-seq, RPPA, MAF, copy profiles by SNP array, clinical data
TCGA uterine corpus endometrial carcinoma (UCEC)	183 tumor samples – RNA-seq, RPPA, MAF, copy profiles by SNP array, clinical data
TCGA uterine carcinosarcoma (UCS)	47 tumor samples – RNA-seq, RPPA, MAF, copy profiles by SNP array, clinical data
Sewell et al. - Head and neck cancer	42 tumor samples – RPPA, clinical data
MDACC Endometrioid carcinoma	178 tumor samples – RPPA, mutation, clinical data
Bergen Endometrioid carcinoma	209 tumor samples – RPPA, clinical data

Supplementary Table 15. Primer sequences used for RT-qPCR analysis

Gene	Primer sequence
ACTR3	5'-CATTCCTGTGGCTGAAGGGT-3'<br>5'-ATCGCTGCATGTGGTGTGTA-3'
FOXP4	5'-GACCCTGTGTGAAGACCTGG-3'<br>5'-GTCAGGGGTTTCCAGGATGG-3'
DDX27	5'-TTGGGGAAGGACATCTGTGC-3'<br>5'-CGGATCCGGATGAACTCCTG-3'
PAPLN	5'-AGGTCATCTGTGCCATTGGG-3'<br>5'-TGTAGAAGCCACTGCCCTTG-3'
PSMB4	5'-GACATGCTGGGATCCTACGG-3'<br>5'-CTTTTTCGGTGACAGTGGCG-3'
WNK1	5'-CTTTTTCGGTGACAGTGGCG-3'<br>5'-CTTGGCTGTTCACTGTTGCC-3'
CDK1	5'-ACAGGTCAAGTGGTAGCCATG-3'<br>5'-GGAGTGCCCAAAGCTCTGAA-3'
CAMKK1	5'-CAGGAAGCTATCTGGAGGCG-3'<br>5'-AAGTACTCGAGGCCCAGGAT-3'
TNFSF10	5-CCTCAGAGAGTAGCAGCTCACA-3'<br>5'-CAGAGCCTTTTCATTCTTGGA-3'
ACTB	5'-CGTCTTCCCCTCCATCGT-3'<br>5'-GAAGGTGTGGTGCCAGATTT-3'
APC	5'-CATTTCCAAGAAGAGGGTTTGT-3'<br>5'-GATCAGCAAGAAGCAATGACC-3'
FBXW11	5'-GGCTGCCGTCAATGTAGTAGA-3'<br>5'-GTGCTCGTGCTCCAGACTT-3'
PPP2R5E	5'-GTGTGTATCTAGCCCCCATTTT-3'<br>5'-AAACTCATGATGTATTCATTATTCCAA-3'
WNT10B	5'-ATGCGAATCCACAACAACAG-3'<br>5'-TCCAGCATGTCTTGAACTGG-3'

Supplementary Table 16. Primer sequences used for ChIP-qPCR analysis

Gene	Primer sequence
ACTR3	5'-TTCGACTCCTGGTCATTTCC-3'<br>5'-CCAAGCACCGGTAATTCAGT-3'
WNK1	5'-GCATGCCCGATTATTCTCTC-3'<br>5'-GCTCCTGGTTCTGCAGGTAG-3'
PAPLN	5'-TGGAGACACTGCTCCTGTCA-3'<br>5'-TGCCCCGATTCTGAATTTAT-3'
FOXP4	5'-GCACAGATGACGAGGACAGA-3'<br>5'-CCTTCTAGGCGCATTCACTC-3'
PSMB4	5'-TCGCCACCTAGGATTACTGC-3'<br>5'-ACGGAATGCGGTAAAACTGT-3'
TNFSF10	5'-AAAGAAAATCCCTCCCCTCTT-3'<br>5-CACTCACCTCAAGCCCATTT-3'
RUNX1	5'-CCTGTGTGATGATGGGTTGTA-3'<br>5'-AAACCGAGGGAGTGTTGTG-3'
ACTB	5'-AGACCTTCAACACCCCAGCC-3'<br>5'-GTCACGCACGATTTCCCGCT-3'
APC	5'-CCCTAGAACCAAATCCAGCA-3'<br>5'-AACATGAGTGGGGTCTCCTG-3'
FBXW11	5'-ATTGCGTGTGTGTGGTTTGT-3'<br>5'-GCCACGACTGCACAGAATAA-3'
PPP2R5E	5'-GGGATGGGAAAGGGATGTAT-3'<br>5'-GTCTGGTGCCACCTTTTTGT-3'
WNT10B	5'-TGGGATGTGTAGCCTTCTCC-3'<br>5'-CCCAGCCAAAAGGAGTATGA-3'

Supplementary References

1 Bindea, G. *et al.* ClueGO: a Cytoscape plug-in to decipher functionally grouped gene ontology and pathway annotation networks. *Bioinformatics* **25**, 1091-1093, doi:10.1093/bioinformatics/btp101 (2009).

2 Wu, X. *et al.* Activation of diverse signalling pathways by oncogenic PIK3CA mutations. *Nat Commun* **5**, 4961, doi:10.1038/ncomms5961 (2014).
