# Supplementary material for: Pancancer modelling predicts the context-specific impact of somatic mutations on transcriptional programs
Source: Nat Commun. 2017 Jan 31;8:14249. doi: 10.1038/ncomms14249 (PMC5290314; doi:10.1038/ncomms14249)
Supplement: Peer Review File [file ncomms14249-s3.pdf]

Editorial Note: this manuscript has been previously reviewed at another journal that is not operating a transparent peer review scheme. This document only contains reviewer comments and rebuttal letters for versions considered at Nature Communications.
